# Microbiome in the hair follicle of androgenetic alopecia patients

**DOI:** 10.1371/journal.pone.0216330

**Published:** 2019-05-03

**Authors:** Bryan Siu-Yin Ho, Eliza Xin Pei Ho, Collins Wenhan Chu, Srinivas Ramasamy, Mei Bigliardi-Qi, Paola Florez de Sessions, Paul Lorenz Bigliardi

**Affiliations:** 1 Experimental Dermatology Group, Institute of Medical Biology, A*STAR (Agency for Science, Technology and Research), Singapore, Singapore; 2 GERMS Platform for microbial genomics, Genome Institute of Singapore, A*STAR (Agency for Science, Technology and Research), Singapore, Singapore; 3 YLL School of Medicine, National University of Singapore and National University Hospital System NUHS, Singapore, Singapore; Kyungpook National University, REPUBLIC OF KOREA

## Abstract

Androgenetic alopecia is the most common form of hair loss in males. It is a multifactorial condition involving genetic predisposition and hormonal changes. The role of microflora during hair loss remains to be understood. We therefore analyzed the microbiome of hair follicles from hair loss patients and the healthy. Hair follicles were extracted from occipital and vertex region of hair loss patients and healthy volunteers and further dissected into middle and lower compartments. The microbiome was then characterized by 16S rRNA sequencing. Distinct microbial population were found in the middle and lower compartment of hair follicles. Middle hair compartment was predominated by *Burkholderia spp*. and less diverse; while higher bacterial diversity was observed in the lower hair portion. Occipital and vertex hair follicles did not show significant differences. In hair loss patients, miniaturized vertex hair houses elevated *Propionibacterium acnes* in the middle and lower compartments while non-miniaturized hair of other regions were comparable to the healthy. Increased abundance of *P*. *acnes* in miniaturized hair follicles could be associated to elevated immune response gene expression in the hair follicle.

## Introduction

Androgenetic alopecia (AGA) features progressive miniaturization of scalp hair, forming a distinct patterned baldness in males [1]. It is the most common form of hair loss in men and is caused by genetic predisposition with elevated androgen activity. Blood-circulating testosterone is metabolized into a more potent form, dihydrotestosterone which acts on the dermal papilla of the hair, inhibiting the duration of hair growth. Hair miniaturization is often accompanied with destruction of the erector muscle and sebaceous gland hyperplasia [2], resulting in an oily surface and often scalp inflammation in form of seborrheic dermatitis [3]. Concomitantly, presence of lymphocyte infiltration, activated T cells in the balding scalp and degranulation of follicular mast cells suggests micro-inflammation or immune response as a cause or consequence of the hair miniaturization process [4, 5]. Micro-inflammation has been speculated to be caused by UV radiation, microbial presence, *IL-6* and androgen signaling activity [6].

Human skin and hair play host to a vast diversity of microorganisms and the establishment of an equilibrium is crucial for health and disease state of skin and hair. Microbiome on human skin surface has been extensively studied as reviewed previously [7, 8]; while less work has covered those in the dermis and hair follicle [9, 10]. By culturing bacterial colonies, it was found that hair follicles house 25% of cutaneous microbial population [11]. Sequencing studies revealed existence of microorganisms down to within the dermis, while hair follicles and eccrine glands house distinct compartmentalized microbial communities. Therefore there is much potential in studying the hair microbiome including the hair structures.

The skin microbiome comprises of commensal microbes and opportunistic pathogens, constantly interacting with the host, eliciting and evading host immune responses. Involvement of microbial activity in skin and hair disorders have been shown in a few instances. *Staphylococcus aureus* is commonly found in skin lesions [12], and its over-representation has been implicated in atopic dermatitis, chronic diabetic wounds and psoriasis [13–15]. Fungal invasion in the hair follicle bulge results in irreversible hair loss and scarring for example in Tinea Capitis. Presence of yeasts, such as *Malassezia* species is associated with increased hair shedding [16], dandruff formation [17] and as well one of the causes for aggravation of atopic dermatitis (as reviewed in [18]). Prevalence of *Propionibacterium acnes* is associated with the pathogenesis of acnes vulgaris [19], and has been reported in cases of hair casts and alopecia [20]. The role of microorganisms in AGA remains unknown. We report the first study characterizing microbiome in the middle and lower hair compartment in healthy and AGA patients to understand the possible link with AGA progression.

## Results

### Follicular biopsy structures from patient and controls

**Fig 1:**
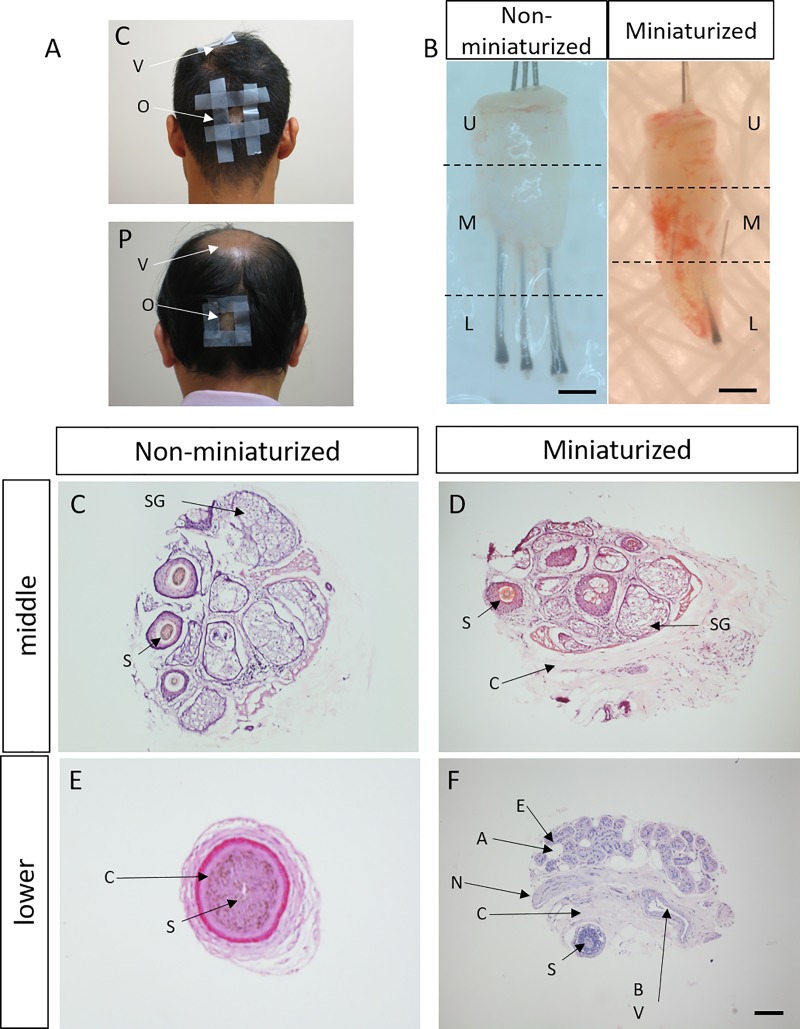
Morphology and histology of FUE used in sequencing study. (A) Representative image of sampling sites of occipital (o) and vertex (v) regions in patients (p) and controls (c). (B) Miniaturized and non-miniaturized hair follicles in AGA patients. Dotted lines represent dissection site of follicular units into upper (U), middle (M) and lower (L) portions. Scale: 0.5 cm. Representative histological image of (C-D) middle portion and (E-F) lower portion of non-miniaturized and miniaturized samples. A: adipose tissue, BV: blood vessel, C: connective tissue, E: eccrine gland, N: nerve, S: Hair shaft, Scale: 100μm.

Using 16S shotgun sequencing, we determined the microbiome in the middle and lower portions of occipital and vertex hair follicles from 20 patients and 10 controls. Healthy hair samples were harvested from occipital and vertex regions of healthy volunteers and in non-balding occipital area of patients by 1mm punches (Fig 1A). Samples taken from patients’ balding vertex regions displayed signs of “miniaturization”, with an altered morphology of a shortened and thinned hair shaft measuring less than 3cm in length (Fig 1B and S1 Fig) [21]. Therefore hair follicles shorter than 3.2cm were classified as miniaturized in this study. Dissection of a follicular unit was performed to obtain microbiome from the lower, middle and upper follicular portions. In non-miniaturized samples, the lower portion contains the hair bulb and small amounts of surrounding subcutaneous and dermal tissues; while miniaturized samples yielded greater biomass including surrounding tissues of blood vessels, nerve endings and sweat glands due to the biopsy sampling method. The middle portion biopsies contained the hair bulge, adipose tissues and connective tissues of the dermis (Fig 1C–1F).

### Microbiome profile from sampling population

**Fig 2:**
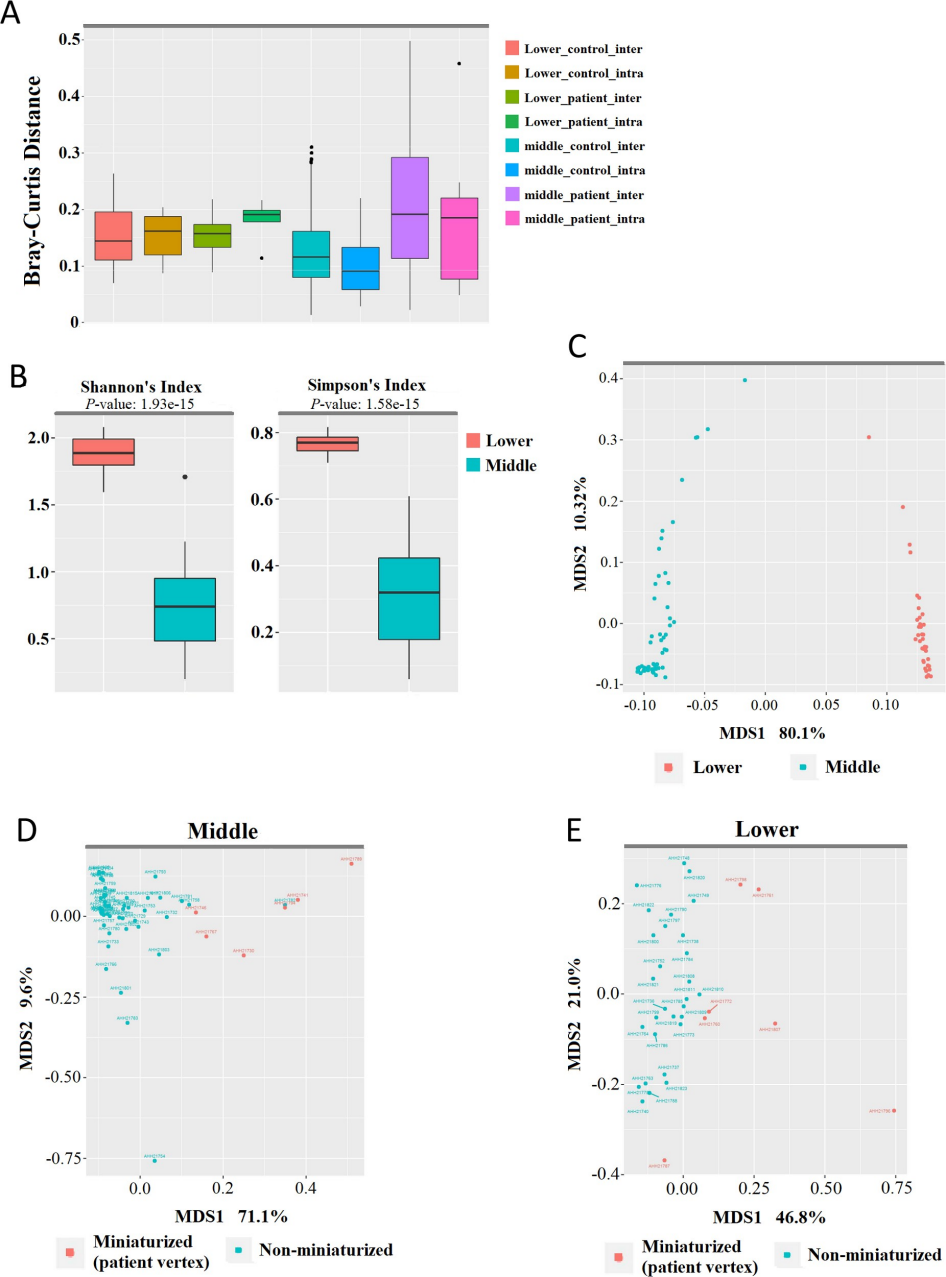
Microbiome profiles of FUE lower and middle portions from AGA patients and healthy controls. (A) Bray-Curtis distances depicting inter and intra-sample dissimilarity of samples from the occipital and vertex regions. (B) Species richness in samples from the lower and middle portions as depicted by Shannon and Simpson’s alpha diversity indices (Mann-Whitney U test, p < 0.005). (C-E). Unweighted UniFrac Principal Coordinate Analyses (PCoA) of all samples, (C) color-coded by lower and middle portion of sample; (D) samples from the middle portion, color-coded by miniaturization status; (E) and samples from the lower portion, color-coded by miniaturization status.

Skin microbiome can vary significantly in different body sites within an individual and across individuals [10, 22]. Using Bray-Curtis dissimilarity analysis, we found relatively low level of dissimilarity in the microbial profile inter and intra-individually, implicating low variation within our patient and control groups, and across hair follicles in the occipital and vertex regions (Fig 2A). Using Shannon’s and Simpson’s indices, alpha diversity was significantly greater in samples from the lower portion than samples from the middle portion (p < 0.005; Fig 2B). Analyses for phylogenetic distances with unweighted Unifrac revealed unique and separate clusters between follicular samples from the lower and middle portions, suggesting distinct microbial populations in the two (Fig 2C).

To observe for microbiota differences in patient and control hair, we performed PCoA on the middle and lower portion samples respectively. In samples from the middle portion, a majority of non-miniaturized samples formed a tight, distinct cluster from miniaturized samples (Fig 2D), indicating a consistent and uniformed colonization in the middle follicular portion regardless of its vertex or occipital origin. Samples from the lower portion display a higher degree of variation between patients and healthy controls, while miniaturized samples consistently clustered independently from non-miniaturized samples (Fig 2E), indicating differences between their respective microbial communities (Fig 2D and 2E). Consistently, classifying samples according to their origin showed close clustering of control occipital, vertex with patient occipital samples while a number of patient vertex samples as outliers (S2A Fig). However, classification by patient age and AGA severity yielded no distinct clustering pattern (S2B and S2C Fig).

**Fig 3:**
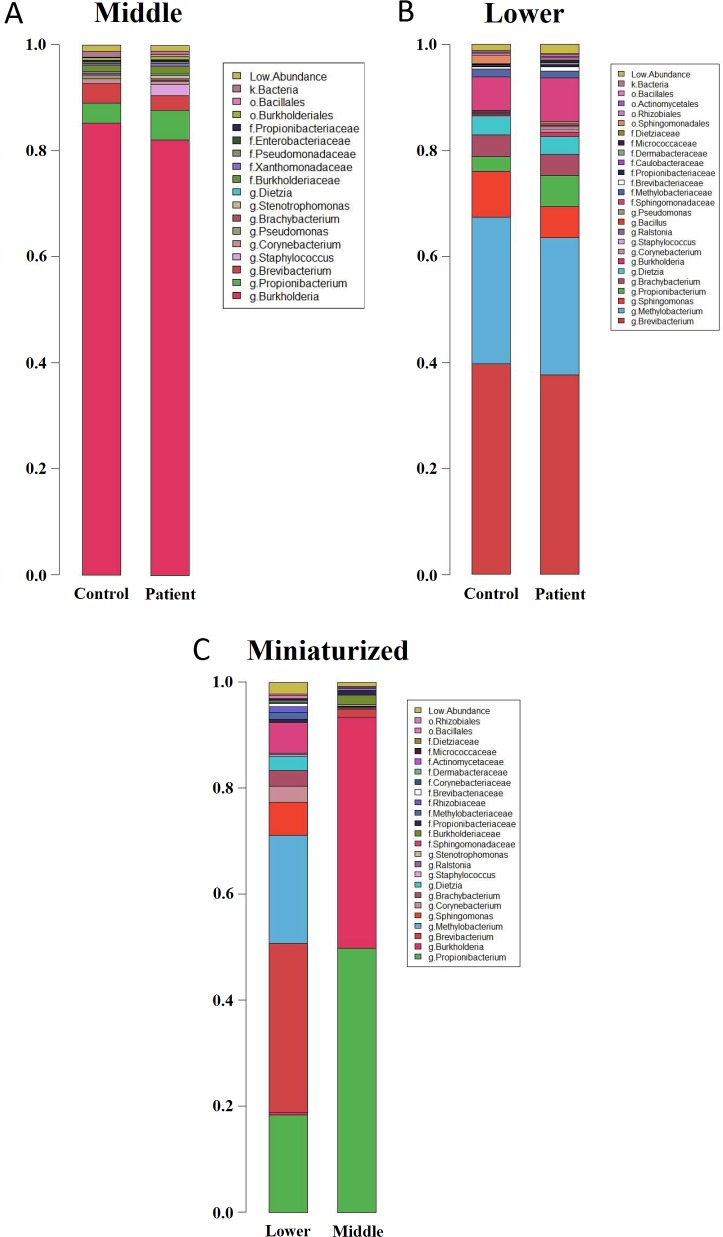
Taxonomic relative abundance across samples. Stacked barplot depicting mean relative abundances in patient and control samples obtained from (A) the middle portion and (B) lower portion of non-miniaturized samples. (C) Relative abundance of lower and middle portions from miniaturized patient vertex samples. Relative abundances are presented in the respective taxonomic ranks–g: Genera; f: Family; o: Order; k: Kingdom.

Members of the microbial community in middle, lower and miniaturized samples were similar. However, relative abundances of each member, and their respective colonization genera, differed between each group and individual (Fig 3, S1 Table). Middle portion of non-miniaturized patients and controls were predominantly colonized by *Burkholderia spp*. (68.1–87.9%) (Fig 3A, Table 1A) Within the *Burkholderia* genera, *B*. *contaminans* (GenBank ID: CP013390.1) and *B*. *cepacia* (GenBank ID: AB211225.1) were the main species identified, along with *B*. *cenocepacia*, *B*. *kururiensis* and they were mutually exclusive in each hair follicle, and we did not find obvious correlation with AGA severity, region or hair follicle compartment for the distribution of different species (S2 Table). A more diverse microbiome resides in the lower portion with predominant genera *Brevibacterium* (34.5–41.0%), *Methylobacterium komagatae* (20.7–25.5%), *Sphingomonas* (3.5–6.6%) (Fig 3B, Table 1B).

**Table 1:** Representative bacterial genera abundance in middle and lower sample portions from patients and controls.

**A**				
Middle portion	CO	CV	PO	PV
g:Burkholderia	82.49%	87.89%	79.26%	68.08%
s:Propionibacterium_acnes	4.59%	2.80%	11.29%	18.89%
g:Brevibacterium	3.70%	3.56%	2.35%	2.44%
f:Burkholderiaceae	1.43%	0.98%	1.60%	1.65%
k:Bacteria	1.37%	0.82%	0.62%	0.34%
g:Corynebacterium	1.32%	0.35%	0.88%	0.17%
g:Pseudomonas	0.95%	0.36%	0.03%	0.31%
o:Burkholderiales	0.76%	0.08%	0.34%	0.79%
g:Brachybacterium	0.43%	0.34%	0.26%	0.24%
g:Dietzia	0.33%	0.32%	0.20%	0.25%
**B**				
Middle portion	CO	CV	PO	PV
g:Burkholderia	82.49%	87.89%	79.26%	68.08%
s:Propionibacterium_acnes	4.59%	2.80%	11.29%	18.89%
g:Brevibacterium	3.70%	3.56%	2.35%	2.44%
f:Burkholderiaceae	1.43%	0.98%	1.60%	1.65%
k:Bacteria	1.37%	0.82%	0.62%	0.34%
g:Corynebacterium	1.32%	0.35%	0.88%	0.17%
g:Pseudomonas	0.95%	0.36%	0.03%	0.31%
o:Burkholderiales	0.76%	0.08%	0.34%	0.79%
g:Brachybacterium	0.43%	0.34%	0.26%	0.24%
g:Dietzia	0.33%	0.32%	0.20%	0.25%

Relative abundance of highly prevalent genera in (A) middle and (B) lower portions from Control Occipital (CO), Control Vertex (CV), Patient Occipital (PO) and Patient Vertex (PV) regions. Values are presented as mean across samples. Taxonomic order is represented as f: family, g: genre and s: species.

**Fig 4:**
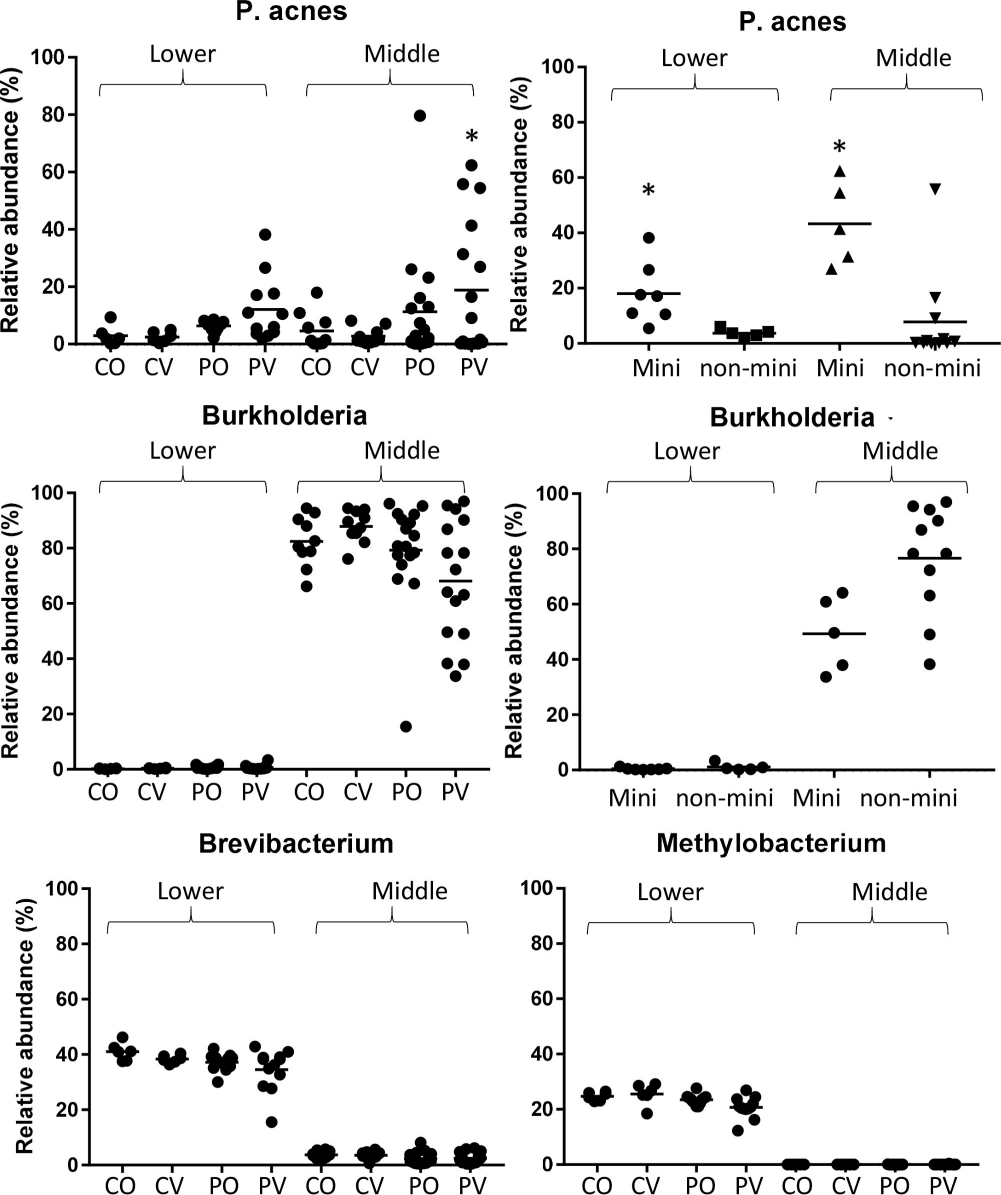
Representative bacterial abundance in hair samples. (A) *P*. *acnes* and (B) *Burkholderia spp*. abundance in hair samples from different regions of origin and miniaturization. (C) *Brevibacterium* and (D) *Methylobacterium* abundance in hair samples from different regions of origin.

The distinct balding pattern in AGA is formed by progressive follicular miniaturization and subsequent balding on the vertex, while hair follicles on the occipital region remains typical. To investigate the association of microbiota with phenotype, PERMANOVA pairwise comparisons were performed between the occipital and vertex regions on patients and controls (Table 2). Overall, relative abundances of various genera in patient vertex (PV) were significantly different from control occipital (CO) and control vertex (CV) samples in middle and lower portions (P-value < 0.05) (Table 2A). We repeated PERMANOVA with the miniaturized samples removed, which resulted in no significant difference in the comparisons (Table 2B), suggesting that the change in microbiota is mainly associated with hair miniaturization. Comparison between the microbiota in miniaturized and non-miniaturized patient vertex samples showed that, in the middle portion, *P*. *acnes* abundance was increased with decreased *Burkholderia* spp. abundance; while *M*. *komagatae*, *Sphingomonadaceae* and *Brevibacterium* were decreased in the lower portion (Table 3A and 3B, Figs 3 and 4). In addition, PO lower portion was significantly different from CO; we found that *P*. *acnes* abundance increased with significance while *Brevibacterium* abundance decreased (Table 3C, Figs 3 and 4).

**Table 2:** Pairwise comparisons for PERMANOVA between samples across different compartments, regions and AGA occurrence.

**A**	Miniaturized included			F.Model	R2	p.value
middle	patient vertex	vs	patient occipital	1.654	0.051	0.191
	patient vertex	vs	control vertex	4.752	0.165	0.016*
	patient vertex	vs	control occipital	3.183	0.117	0.046*
	patient occipital	vs	control occipital	0.995	0.038	0.379
	patient occipital	vs	control vertex	1.897	0.071	0.123
	control occipital	vs	control vertex	1.212	0.063	0.262
lower	patient vertex	vs	patient occipital	1.749	0.074	0.126
	patient vertex	vs	control vertex	2.844	0.151	0.033*
	patient vertex	vs	control occipital	2.660	0.143	0.043*
	patient occipital	vs	control occipital	2.367	0.129	0.045*
	patient occipital	vs	control vertex	2.114	0.117	0.058
	control occipital	vs	control vertex	0.825	0.076	0.563
**B**	Miniaturized removed			F.Model	R2	p.value
middle	patient vertex	vs	patient occipital	0.818	0.032	0.512
	patient vertex	vs	control occipital	0.699	0.035	0.668
	patient vertex	vs	control vertex	1.513	0.074	0.147
	patient occipital	vs	control occipital	2.162	0.083	0.112
	patient occipital	vs	control vertex	0.863	0.035	0.443
	control occipital	vs	control vertex	1.212	0.063	0.258
lower	patient vertex	vs	patient occipital	1.014	0.063	0.409
	patient vertex	vs	control occipital	1.660	0.156	0.116
	patient vertex	vs	control vertex	1.250	0.122	0.296
	patient occipital	vs	control occipital	2.367	0.129	0.06
	patient occipital	vs	control vertex	2.114	0.117	0.039*
	control occipital	vs	control vertex	0.825	0.076	0.568

Pairwise comparisons between (A) Middle and lower portion of all CO, CV, PO and PV samples. (B) Middle and lower portion PV samples versus other samples with miniaturized samples removed. P-value < 0.05 is significant.

**Table 3:** Representative comparisons for bacteria abundance between middle portion hair samples.

A					
mini vs non-mini (mid)	mini	non-mini	mini (stdev)	non-mini (stdev)	t-test
s:Propionibacterium_acnes	43.30%	7.79%	14.99%	16.75%	0.001*
g:Propionibacterium	0.47%	0.08%	0.29%	0.17%	0.004*
g:Burkholderia	49.26%	76.64%	13.48%	19.51%	0.014*
f:Propionibacteriaceae	0.92%	0.29%	0.56%	0.51%	0.042*
B					
mini vs non-mini (low)	mini	non-mini	mini (stdev)	non-mini (stdev)	t-test
s:Bifidobacterium_breve	0.00%	0.02%	0.00%	0.01%	0.015*
s:Methylobacterium_komagatae	18.67%	23.59%	1.22%	1.03%	0.015*
s:Propionibacterium_acnes	18.08%	3.74%	4.22%	0.70%	0.018*
f:Sphingomonadaceae	5.77%	10.09%	1.41%	1.51%	0.067
g:Brevibacterium	31.79%	38.30%	3.39%	0.99%	0.148
C					
CO vs PO (low)	CO	PO	CO (stdev)	PO (stdev)	t-test
s:Propionibacterium_acnes	2.96%	6.38%	3.40%	1.84%	0.013*
g:Cellulomonas	0.03%	0.00%	0.04%	0.01%	0.014*
g:Brevibacterium	41.01%	37.18%	3.21%	3.11%	0.027*
f:Rhizobiaceae	0.03%	0.00%	0.05%	0.01%	0.032*
o:Sphingomonadales	0.49%	0.10%	0.58%	0.11%	0.036*
f:Brevibacteriaceae	0.34%	0.87%	0.08%	0.60%	0.049*

Taxonomic order represented as f: family, g: genre and s: species. Bacteria abundance comparisons between miniaturized and non-miniaturized in (A) middle portion and (B) lower portion. (C) Comparisons between lower portion PO and CO. Data presented as mean and standard deviation across samples, P-value < 0.05 is significant.

**Fig 5:**
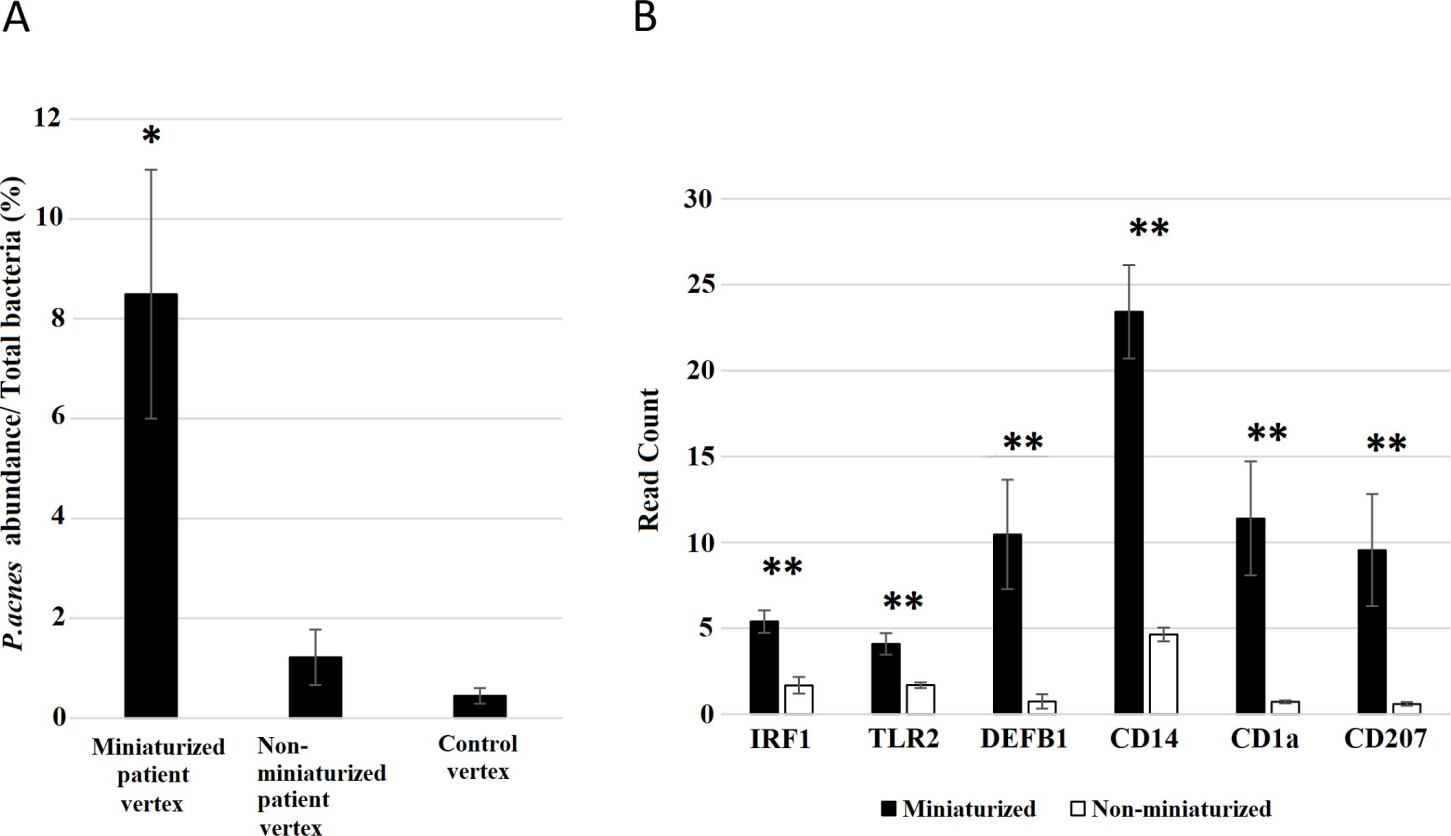
Expression of immune response genes across miniaturized and non-miniaturized hair follicles. Bar graphs depicting read counts of *TLR2*, *IRF1*, *DEFB1*, *CD1A* and *CD14* in patient matched miniaturized and non-miniaturized vertex samples, data is presented as mean ± SEM. *P-value < 0.05.

It has been reported that *P*. *acnes* and residential microflora of the hair and skin can elicit innate immune responses through toll like receptor 2 (*TLR2*) and up-regulation of anti-microbial peptides including β-defensin (*DEFB1*) [23, 24]. Through RNA sequencing of lower portion subset samples, several genes in antigen presentation and Th-1, Th-2 inflammatory pathways were found to be elevated in miniaturized hair follicles [25, 26]. Hence, we analyzed transcriptomic expression for genes involved in microbial response, the expression level of genes involved in responses to microbes such as Toll Like Receptor-2 (*TLR2*), *DEFB1*, Interferon regulatory factor 1 (*IRF1*), monocyte marker CD14, and Langerhans cell marker CD1a/CD207 were increased in miniaturized compared to non-miniaturized hair follicles [27–29] (Fig 5). These changes suggest that elevated immune responses and immune cell infiltration corresponded with increased *P*. *acnes* abundance.

## Discussion

Microbial community on the skin surface has been well characterized while less is understood about the hair follicle micro-environment. In literature, microbiota from plucked hair samples or entire hair follicles from skin sections has been analyzed [9, 10]. These studies provide an insight regarding the follicular microbiota profile in an Asian male cohort in Singapore (n = 30). Concordant with the literature, a diverse microbial community and formation of bacterial biofilms can be detected in the dermis and dermal sections of the hair follicle [10, 30]. Bacterial abundance in the middle portion may be due to the close proximity to the skin surface with rich microflora. Higher bacterial diversity in the lower portion is unexpected but may play a more important role in hair pathogenesis due to the dense vascular network around the hair papilla and therefore more potential interactions with the host immune system. It may implicate a complex interaction with the host immune response. Interestingly, unlike previous reports, we did not observe significant inter-individual variation of microbiota in our study [10, 31]. We also report a slightly different bacterial population from other studies, such differences may originate from the method of sample collection; for the hair follicle is extracted under sterile conditions with a biopsy punch of the hair follicle with adjacent tissue (blood vessels, nerve fibers, connective tissue and sebaceous glands) while others utilized laser-capture microscopy or plucked hair.

Multiple steps from sample collection to result analysis are integral in studies of the skin microbiome [32]. During sample collection, patient scalps have undergone disinfection prior to follicular unit extraction, thereby removing potential contamination by bacteria on the skin surface. Additionally, the choice of primer may influence sequencing results. The difference in the representation of *Propionibacterium* and *Staphylococcus* with primers targeting the hypervariable V1-V3 region and the V3-V4 region has been reported [33]. Other studies have suggested that the V3-V4 region can accurately represent the skin microbiome [34, 35]. We employed primers amplifying the V3-V6 regions on the 16S rRNA to improve taxonomic resolution with longer amplicons and expect biases toward specific genera to be minimal [36]. The impact of reagent contamination on microbiome analysis has been noted to distort the distributions and frequencies of identified bacterial species [33, 37]. To address this issue, we included template free controls in the sequencing analysis. It yielded read counts less than the cutoff for subsequent analysis, thus ensuring the quality of study outcomes.

Concordant with studies using scalp and plucked hair samples, members from the Burkholderia genera were found to be highly abundant in follicular samples from the middle portion [9, 10]. *Burkholderia cepacia* and *Burkholderia contaminans* have been isolated from patients with cystic fibrosis and pneumonia, however, it was not known to be associated with skin-related disorders [38, 39]. Dominant species on the skin surface such as *Propionibacterium*, *Corynebacterium* and *Staphylococcus* occurred in this study at a low frequency, which may reflect a preference for inhabiting the skin surface over deeper layers within the skin. The skin microbiome has demonstrated topographic variability by skin physiology of sampling sites including moisture, temperature and pH; while remaining relatively stable over time [31, 40–42]. The occipital and vertex scalp may present different skin micro-environment to the microbiome as they originate from different embryonic developmental plates and are exposed to varying degrees of solar irradiation. AGA patients typically feature a patterned baldness whereby lower hair density, hair thinning and yellow dots depicting enlarged sebaceous glands in response to androgen are evident in the vertex scalp area [43, 44]. Despite the changes, the microbiome in AGA patients did not differ significantly between different sites on the scalp; nor was it significantly unique according to individual age and AGA severity. Rather, distinct microbial profiles from miniaturized hair samples were observed in this study. The data suggests that changes specific to hair miniaturization can cause disruptions to the microbiome. In addition, the lower portions of non-miniaturized samples contain clean hair bulbs while miniaturized samples would include surrounding tissues including sweat glands and blood vessels which may house significantly different microbial populations. However, increased abundance of *P*. *acnes* could be observed in the middle portion of miniaturized and non-miniaturized samples where they shared similar histology with sebaceous glands and adipose tissues. Further study has to be performed to investigate specific changes in miniaturized hair associated with *P*. *acnes* abundance.

It has long been speculated that *P*. *acnes* is involved in AGA pathogenesis [6, 45, 46]. In our study we report for the first time such striking increase in abundance in miniaturized hair. *P*. *acnes* is a common opportunistic pathogen found on the skin surface, and is prevalent in the hair follicle and the associated pilosebaceous unit [47, 48]. *P*. *acnes* predominance is also identified in non-lesional scalp of patients with seborrheic dermatitis [49]; providing further support for the development in sebaceous gland hyperplasia in AGA may attract the proliferation of *P*. *acnes*; for lipids and fatty acids are its main nutrient sources [50]. Alternatively, as in the case of acnes vulgaris, another testosterone metabolism-related disease, altered sebum composition with an increase in peroxidized squalene has been suggested to result in *P*. *acnes* proliferation [51–53], and such changes may occur in AGA. *P*. *acnes* induces inflammatory responses and is known as one of the causative factors for *acnes vulgaris*. Virulence in the hair follicle is demonstrated to cause hair casts and hair loss as a consequence [20]. Additionally, it is interesting to note the increase in *P*. *acnes* abundance in occipital samples, suggesting the possibility that increased sebum on scalp environment may result in changes in the microbiome over the scalp. Additionally, phylogenetic analysis revealed that *P*. *acnes* comprises of four distinct lineages; each displaying differences in inducing inflammatory responses and virulence determinants [54, 55]. Further characterization to the strain level will be essential to decipher its role in AGA pathogenesis.

Evidence of micro-inflammation such as perifollicular inflammatory infiltration, prostaglandin and cytokine elevation have been observed in hair follicles of AGA patients and is suggested to cause hair miniaturization [6, 56, 57]. A combination of environmental factors including UV radiation, allergen exposure and porphyrin production have been considered to elicit inflammation [46, 58], whereby inflammatory factors are believed to hinder hair growth. Our data on the presence of *P*. *acnes* adds to the contributing factors for micro-inflammation in hair loss. Recent studies investigating microbiome-host immune interactions suggest that the microbiome induces inflammatory cytokine production in host gut and moderates T lymphocyte function in mouse skin [59, 60]. The presence of Langerhans cells also indicate induction of the innate immune response as they are majorly involved in antigen presentation. These evidence indicate that the microbiome may play a more important and complex role than previously imagined. Interestingly, reports of effective AGA treatments by anti-microbial solutions and Ketoconazole [61, 62] has been suggestive of the involvement of microorganisms in AGA besides its anti-androgen properties. In addition to the genetic causes of AGA, *P*. *acnes* may act as an environmental factor for AGA pathogenesis and presents a novel candidate in treating AGA. Future studies on the impact of changes in other bacterial species and the imbalance are equally important in deciphering the pathogenesis of AGA. Since hair follicles can be compartmentalized into the bulge, matrix and dermal papilla, it will be essential to visualize the distribution of hair follicle microbiome with higher clarity to the compartments. Further information will aid understanding the association between microbiome and hair conditions.

## Conclusion

We reported distinct microbial population in the middle and lower portion of the hair follicle. *Burkholderia* genera predominates the middle portion while higher microbial diversity was observed in the lower portion. In AGA patients, miniaturized patient vertex hair houses elevated *P*. *acnes* while hair from other regions were comparable. This is the first study characterizing the microbiome in AGA and provides new insight into the condition.

## Methods

### Sample collection, DNA extraction and sequencing

Twenty AGA patients and 10 healthy volunteers were recruited in the study previously described [25]. Ethics approval and consent to participate were obtained through the National Healthcare Group Review Board (NHG-DSRB “2012/00488 Transcriptome and genome analysis of human scalp biopsies of androgenetic alopecia before and after topical laser treatment”). All methods were carried out in accordance with the approved protocol. After thorough local disinfection with 70% isopropanol, follicular unit extractions were performed in the vertex and occipital areas with a punch of 1.1mm diameter. The extracted follicular units were imaged using a dissection microscope (Zeiss) with mounted digital camera and then divided into 3 parts (hair papilla, middle piece with sebaceous gland and upper piece with ostium and epidermis) and immediately snap frozen in liquid nitrogen.

To assess any alterations of the microbiome population in the hair follicle, DNA was extracted from the lower (hair bulb) and middle (with sebaceous glands) piece of the follicular unit. Follicular unit sample were subjected to bead beating (MP Biomedicals) and gDNA extraction using AllPrep RNA/DNA extraction kit following the manufacturer’s instructions (Qiagen). 16S rRNA PCR was performed on samples and duplicate template-free controls using primers spanning V3-V6 regions with PCR conditions described previously [36, 63]. DNA sequencing libraries were constructed with QIAseq FX DNA library kit (96) (Qiagen). DNA libraries were paired-end sequenced on the Illumina HiSeq Rapid (2x76bp). Following demultiplexing (using Illumina bcl2fastq 2.17.1.14 software) and removal of reads that failed Illumina’s purity filters (PF = 0), reads were converted to FASTQ. 16S rRNA sequencing of the samples yielded an average of 452,193 read counts, while 4 samples and template-free control libraries which yielded less than 50,000 read counts were subsequently discarded. Trimming of reads was done by removing trailing bases with quality score ≤ 2; read pairs with reads shorter than 60bp were also removed.

#### 16S rRNA amplicon sequence reconstruction

To reconstruct 16S amplicon sequences, we utilized the processing methods described in Ong et al [18). Trimmed reads were input into EMIRGE (GIT version 98787b5). EMIRGE performs template-guided “assembly” based on a modified SILVA SSU (version 102) database, and utilises an expectation-maximization algorithm for iteration, alignment, and classification of reads to candidate 16S sequences [64]. Iterative mapping of paired-end reads also prevents chimeric sequences from mapping. This reconstruction methodology has been compared against RTAX and modQIIME, and was able to robustly produce highly concordant estimates of taxonomic OTU abundance [18].

We applied EMIRGE to the top (in terms of average quality) 500,000 reads in each sample; we had previously found this number to be robust enough to accurately reflect the 16S composition in each sample [18]. Sequences with relative abundance below 0.1% were removed. Finally, the reconstructed amplicon sequences were searched using BLAST against the Greengenes 16S rRNA database [65] BLAST hits were sorted in consecutive order, smallest E-value, highest bit score, highest percent identity, and longest alignment length; only the top hit after this sorting was used for classification. Percentage identities for phylum, family, and genus levels were 75%, 86.5%, and 94.5% respectively.

#### Abundance determination of microbial community

Abundance estimates were assigned to reconstructed sequences using EMIRGE, for generation of abundance profile of OTUs for each sample. A data matrix containing relative abundances, with each sample as a row and each genus as a column, was used to generate the relative abundance barplots and alpha diversity boxplots using the R software, version 3.4.0. The Principal Coordinate Analyses (PCoA) with Bray–Curtis distance was applied using the “vegan” package in R.

### Sample processing and histology

Dissected follicular units were fixed in 4% paraformaldehyde. Samples were dehydrated and embedded in paraffin for sectioning at 5μm thick. Hematoxylin and eosin staining was performed on hair sections and imaged by Zeiss AxioImager (Zeiss).

## Supporting information

S1 Fig Length of hair samples. Length of hair follicle samples for A. middle piece and B. lower piece used in the analysis. Miniaturized hair lower than 3.2cm are classified miniaturized in this study.
(PDF)

S2 Fig Clustering of hair samples. PCoA plot of middle (left) and lower (right) hair samples labeled according to (A) Region (grouped into control occipital, vertex and patient occipital, vertex) (B) AGA severity (grouped into Norwood Hamilton scale 3–4; 5–6 and healthy) (C) Age (control 20–40; 40–60 and patient 20–40; 40–60). Samples were marked with sample number.
(PDF)

S1 Table Members of the microbial community with corresponding relative abundance in each sample. (PDF)

S2 Table Predominant *Burkholderia* species in hair samples. Predominant *Burkholderia* species in middle and lower piece of vertex and occipital hair from patient and healthy controls.
(PDF)

## Abbreviations

PV: Patient vertex
PO: patient occipital
CV: control vertex
CO: control occipital
AGA: Androgenetic alopecia
PCoA: Principal Coordinate analysis
