# Supplementary material for: Microbiome in the hair follicle of androgenetic alopecia patients
Source: PLoS One. 2019 May 3;14(5):e0216330. doi: 10.1371/journal.pone.0216330 (PMC6499469; doi:10.1371/journal.pone.0216330)
Supplement: S1 Fig — Length of hair follicle samples for A. middle piece and B. lower piece used in the analysis. Miniaturized hair lower than 3.2cm are classified miniaturized in this study. (PDF) [file pone.0216330.s001.pdf]

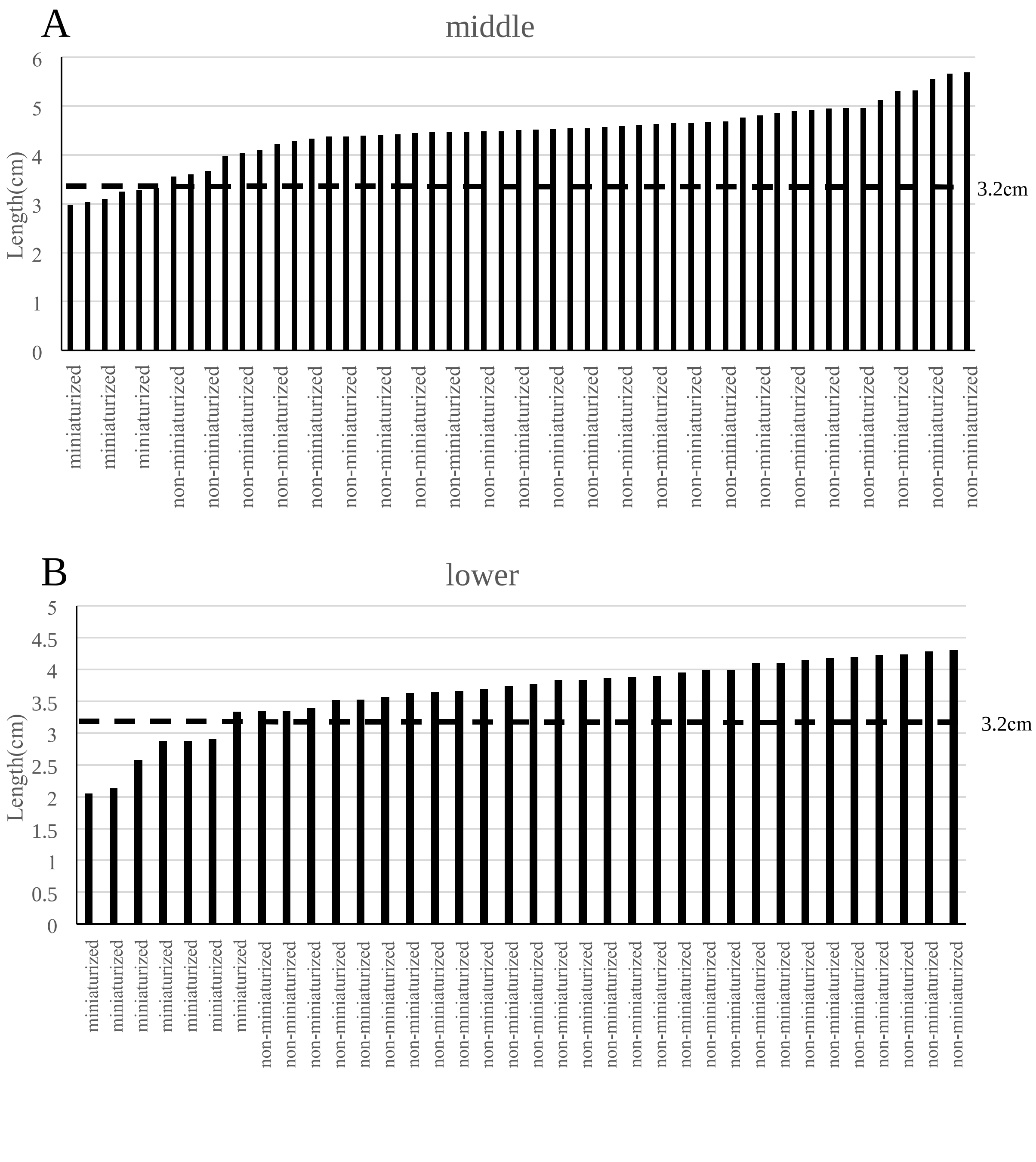

S1 Fig. Length of hair samples. Length of hair follicle samples for A. middle piece and B. lower piece used in the analysis. Miniaturized hair lower than 3.2cm are classified miniaturized in this study.
