# Supplementary material for: Microbiome in the hair follicle of androgenetic alopecia patients
Source: PLoS One. 2019 May 3;14(5):e0216330. doi: 10.1371/journal.pone.0216330 (PMC6499469; doi:10.1371/journal.pone.0216330)
Supplement: S2 Fig — PCoA plot of middle (left) and lower (right) hair samples labeled according to (A) Region (grouped into control occipital, vertex and patient occipital, vertex) (B) AGA severity (grouped into Norwood Hamilton scale 3–4; 5–6 and healthy) (C) Age (control 20–40; 40–60 and patient 20–40; 40–60). Samples were marked with sample number. (PDF) [file pone.0216330.s002.pdf]

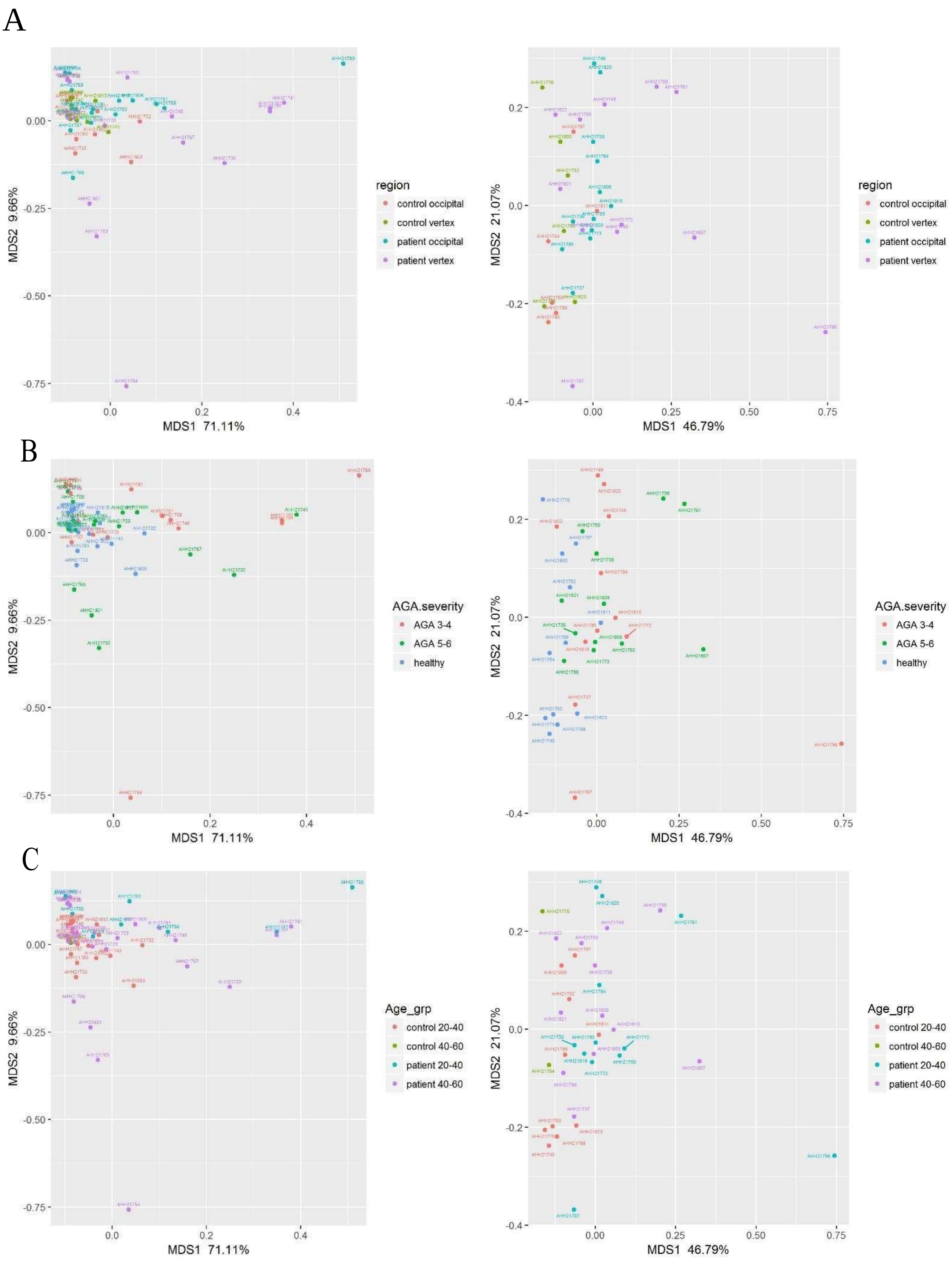

S2 Fig. Clustering of hair samples. PCoA plot of middle (left) and lower (right) hair samples labeled according to (A) Region (grouped into control occipital, vertex and patient occipital, vertex) (B) AGA severity (grouped into Norwood Hamilton scale 3-4; 5-6 and healthy) (C) Age (control 20-40; 40-60 and patient 20-40; 40-60). Samples were marked with sample number.
