# Supplementary material for: Microbiome in the hair follicle of androgenetic alopecia patients
Source: PLoS One. 2019 May 3;14(5):e0216330. doi: 10.1371/journal.pone.0216330 (PMC6499469; doi:10.1371/journal.pone.0216330)
Supplement: S1 Table — (PDF) [file pone.0216330.s003.pdf]

bac_name	AHH21787	AHH21760	AHH21772	AHH21811	AHH21810	AHH21808	AHH21784	AHH21809	AHH21820
region	lower mini	lower	lower	lower	lower	lower	lower	lower	lower
c:Alphapro	0	0	0.000368	0	0	0	0	0	0
c:BD1-5	0	0	0	0	0	0	0	0	0
c:TM7-3	0	0	0	0	0	0	0	0	0
f:Acetobac	0	0	0	0	0	0	0	0	0
f:Acidobact	0	0	0	0	0	0	0	0	0
f:Actinomy	0	0	0	0	0	0	0	0	0
f:Actinosyn	0	0	0	0	0	0	0	0	0
f:Aerococca	0	0	0	0	0	0	0	0	0
f:Alcaligena	0	0	0	0	0	0	0	0	0
f:Bacillacea	0	0	0	0	0	0.000249	0	0	0
f:Beijerinck	0	0	0	0	0	0	0	0	0
f:Beutenbe	0	0	0	0	0	0	0	0	0
f:Bifidobac	0	0.000619	0	0	0	0	0	0	0.000137
f:Bradyrhiz	0	0	0	0.002528	0.001238	0	0.000438	0.00091	0.00207
f:Brevibact	0.002987	0.002497	0.014343	0.004241	0.010896	0.010371	0.00603	0.003377	0.008632
f:Brucellace	0	0	0	0	0	0	0	0	0
f:Burkholde	0	0	0	0.00022	0	0.000147	0	0	0.000233
f:Caulobact	0.001427	0	0.002518	0.001068	0.001264	0.001592	0.001746	0.004614	0.000794
f:Cellulomo	0	0	0	0	0	0	0	0	0
f:Clostridia	0	0	0	0	0	0	0	0	0
f:Comamon	0.003277	0	0.002631	0.000794	0.000182	0.002289	0.001465	0.000993	0.000387
f:Conexibac	0	0	0	0	0	0	0	0	0
f:Coryneba	0	0.000807	0.001542	0	0.00056	0	0.000415	0.000449	0.000101
f:Dermabac	0	0.001124	0.001985	0.000817	0.001846	0.0016	0.002428	0.004219	0.00427
f:Dermacoc	0	0	0	0	0.00073	0	0	0.000698	0.000437
f:Dermatop	0	0	0	0	0	0	0	0	0
f:Dietziacea	0.002988	0.003853	0.000455	0.001538	0.001101	0.000442	0.002702	0.001619	0.000609
f:Enterobac	0.000659	0	0	0.00078	0	0	0.00065	0	0.000326
f:Erythroba	0	0	0	0	0	0	0	0	0.00038
f:Flavobact	0	0	0	0.001791	0	0	0	0	0
f:Frankiace	0.000768	0	0	0	0	0	0	0	0
f:Gemellac	0	0	0	0	0	0	0.000786	0	0
f:Gordonia	0	0	0.001218	0	0.000621	0.000581	0.000985	0	0.000233
f:Halomona	0	0	0	0	0	0	0	0	0
f:Hyphomic	0	0	0	0	0	0	0	0	0
f:Intraspora	0	0	0.000271	0.000204	0	0	0.000551	0.001019	0.000532
f:Kineospor	0	0	0	0	0	0	0	0	0
f:Ktedonob	0	0	0	0	0	0	0	0	0
f:Lactobaci	0	0	0	0	0	0	0	0	0
f:Leuconos	0	0	0	0	0	0	0	0	0
f:Methylob	0.015033	0.007293	0.034446	0.002948	0.025028	0.018365	0.013037	0.019206	0.010468
f:Methyloc			0.001411	0	0	0	0	0	0
f:Microbac			0	0	0	0	0.000748	0.000354	0.000348
f:Micrococc			0.004648	0.000217	0.001005	0.001965	0.001969	0.005142	5.01E-05
	0	0							
	0	0							
	0	0							
	0	0	0	0		0	0	0	0

	0	0	0	0	0			0	0
	0	0	0	0	0			0	0
	0	0	0						
	0	0							
	0	0							
f:Micromon					0				
f:Moraxella						0	0		
f:Mycobact						0	0		
f:Neisseriac				0	0	0	0	0	0
f:Nocardiac			0.001212	0	0	0	0	0	0
f:Nocardioi			0	0	0.000797	0	0	0.000926	0.001281
f:Oxalobac	0.001013	0.000197	0.001027	0.000276	0.000233	0.00036	0	0.000122	0.003461
f:Phyllobac	0	0	0	0	0	0	0	0	0
f:Planococc	0	0	0	0	0.000181	0	0	0	0
f:Promicrom	0	0	0	0	0	0.000432	0	0	0
f:Propionib	0	0.008082	0.001276	0.000309	0.021182	0.000164	0.001091	0	0.001107
f:Pseudom	0	0	0.000676	0	0	0.001259	0	0	0
f:Pseudono	0	0	0	0	0	0	0	0.000109	0
f:Rarobacte	0	0	0	0	0	0	0	0	0
f:Rhizobiac	0.075267	0	0	0.000516	0	0	0	0	0
f:Rhodobac	0	0	0	0	0	0	0	0	0
f:Rhodocyc	0	0	0	0	0	0	0	0	0
f:Rhodospi	0	0	0	0	0	0.000455	0	0	0
f:Sanguibac	0	0	0	0	0	0	0	0	0
f:Shewanel	0	0	0	0	0	0	0	0	0
f:Sinobacte	0	0	0.000113	0	0.000165	0	0.001659	0	0.000679
f:Sphingob	0	0	0	0	0	0	0	0	0
f:Sphingom	0.011913	0.069171	0.06647	0.065007	0.071096	0.068171	0.089284	0.056755	0.144502
f:Staphyloc	0	0.000288	0.000765	0	0.001746	0	0	0	0
f:Streptoco	0	0	0	0	0	0	0	0.00081	0.000485
f:Streptom	0	0	0	0	0	0	0	0	0
f:Syntropha	0	0	0	0	0	0	0	0	0
f:Tissierella	0	0	0	0	0	0	0.00026	0	0
f:Tsukamur	0	0	0	0	0	0	0	0	0
f:Weeksella	0	0	0	0	0	0	0	0	0
f:Williamsia	0	0	0	0	0	0	0	0	0
f:Xanthoba	0	0	0	0	0	0	0	0	0.000133
f:Xanthomo	0	0	0	0	0	0	0	0	0
f:Xenococc	0	0	0	0	0	0	0	0	0
g:Achromo	0	0	0	0	0	0	0	0	0
g:Acidisom	0	0	0	0	0	0	0	0	0.000139
g:Acidocell	0	0	0	0	0	0	0.000432	0	0
g:Acinetob	0	0	0.000741	0	0.000544	0.000905	0	0.00074	0.001808
g:Actinoba	0	0	0	0	0	0	0	0	0
g:Actinomy	0	0	0	0	0	0.000554	0	0.000884	0
g:Afipia	0	0.000975	0	0	0.000827	0.00046	0.001325	0	0.000884
g:Agrobact	0	0	0.000708	0.001509	0	0	0	0	0
g:Anaeroco	0	0	0	0	0	0.000445	0.000607	0	0

		0	0						0
g:Arthroba	0	0.002951	0	0	0	0	0	0	0
g:Bacillus	0	0	0	0	0	0	0	0	0
g:Bifidobac	0	0	0.000539	0.000045	0	0	0	0	0.000148
g:Bosea	0	0	0	0	0	0	0	0	0
g:Brachyba	0.053314	0.02306	0.037099	0.042045	0.036114	0.04124	0.042477	0.041999	0.032463
g:Bradyrhiz	0.005313			0.000317	0.000234	0.001249	0	0.000504	
g:Brevibact	0.428641	0.38872	0.36226	0.375643	0.36494	0.351552	0.391763	0.385734	0.300577
g:Brevundi	0	0	0	0	0	0	0	0	0
g:Burkhold	0.002488	0.003085	0.00462	0.000768	0.009101	0.01732	0.004339	0.001629	0.001196
g:Candidat	0	0	0	0.000205	0	0	0	0	0
g:Capnocyt	0	0	0	0	0	0	0	0	0
g:Cardioba	0	0	0	0	0	0	0	0	0
g:Caulobac	0	0	0.00054	0	0	0	0	0	0
g:Cellulomo	0	0	0	0.000479	0	0	0	0	0.000184
g:Cellvibrio	0	0	0	0	0	0	0	0	0
g:Chryseob	0	0	0	0	0	0	0	0	0
g:Clavibact	0	0	0	0	0	0	0	0	0
g:Comamo	0	0	0	0.001112	0	0	0	0	0.001964
g:Coryneba	0.003986	0.004079	0.012813	0	0.010399	0.002455	0.014237	0.004768	0.000408
g:Couchiop	0	0	0	0	0	0	0	0	0
g:Cryocola	0	0	0	0	0	0.000697	0	0	0
g:Cupriavid	0	0	0	0	0	0	0	0	0
g:Deinococ	0	0	0	0	0	0.000186	0	0	0
g:Dermaba	0	0	0	0	0	0	0	0	0
g:Dermaco	0	0.001201	0	0.000608	0	0.000747	0	0.000684	0
g:Devosia	0	0	0	0	0	0	0	0	0
g:Dietzia	0.041428	0.035475	0.029371	0.031913	0.033941	0.031057	0.041682	0.029959	0.034474
g:Dyella	0	0	0	0	0	0	0	0	0
g:Dysgonom	0	0	0	0	0	0	0	0	0
g:Edaphoba	0	0	0	0	0	0	0	0	0
g:Enhydrob	0	0	0	0.000319	0	0	0	0	0.001002
g:Facklamia	0	0	0	0	0	0	0	0	0
g:Finegoldi	0	0	0	0	0	0	0	0	0
g:Fusobact	0	0	0	0	0	0	0	0	0
g:Gardnere	0	0.0003	0	0	0	0	0	0	0
g:Gemella	0	0	0	0	0.000482	0	0	0	0
g:Geobacill	0	0	0	0	0	0	0	0	0
g:Gordonia	0	0	0.001602	0	0	0.001403	0	0	0.001444
g:Granulica	0	0	0	0	0	0	0	0	0
g:Haemoph	0	0	0	0	0	0	0	0	0
g:Hydrogen	0	0	0	0	0	0	0	0	0
g:Janibacte	0	0	0.000823	0	0	0	0	0	0
g:Janthinob	0	0	0	0	0	0	0	0	0
g:Kaistobac	0	0	0	0	0	0	0	0	0.001675
g:Kitasatos	0	0	0	0	0	0	0	0	0
g:Klebsiella	0	0	0	0	0	0	0	0	0.000978
g:Kocuria	0	0	0	0	0	0	0	0	0
	0	0							
	0	0							
	0	0							
	0	0	0	0		0	0	0	0

	0	0	0	0	0			0	0
	0	0	0	0	0			0	0
	0	0	0						
	0	0							
	0	0							
g:Kytococc	0	0	0	0	0	0	0.000306	0	0
g:Labrys			0	0	0	0	0	0	0
g:Lautropia			0	0	0.000171	0	0	0	0
g:Leptothri			0	0	0	0	0	0	0
g:Leptotric					0.000715				

	0	0	0	0	0			0	0
	0	0	0	0	0			0	0
	0	0	0						
	0	0							
	0	0							
	0								
	0								
	0								
g:Leucobac						0.00058	0.000696		
g:Leuconos						0	0		
g:Luteibact				0	0	0	0	0	0
g:Marinoba			0.000845	0	0	0	0	0	0
g:Mesorhiz			0	0	0	0	0	0	0
g:Methylob		0.021245	0.003147	0.028031	0.000158	0.020741	0.025293	0.013769	0.028374
g:Microbac		0	0	0.00058	0.000928	0	0	0	0
g:Microbisp		0	0	0	0	0	0	0	0
g:Micrococ	0	0.001805	0	0	0	0	0	0	0
g:Microlun	0	0	0	0	0	0	0	0	0
g:Mycobac	0	0	0	0	0	0	0.000418	0	0.000402
g:Mycoplas	0	0	0	0	0	0	0	0	0
g:Myroides	0	0	0.001424	0	0	0	0	0	0
g:N09	0	0	0	0	0	0	0	0	0
g:Neisseria	0	0	0	0	0	0	0	0	0
g:Nesteren	0	0	0	0	0	0	0.000306	0.000398	0
g:Nocardio	0	0	0	0	0	0	0	0	7.01E-05
g:Novispiril	0	0	0	0	0	0	0	0	0
g:Novosphi	0	0	0	0	0	0	0	0	0
g:Oceanob	0	0	0	0	0	0	0	0	0
g:Ochrobac	0	0	0	0	0	0	0	0	0
g:Paenibac	0	0	0	0.000472	0.000578	0.00049	0.000609	0	0.000388
g:Pandorae	0	0	0	0	0	0	0	0	0
g:Paracocc	0	0	0	0.000561	0	0.000176	0	0	0
g:Parvimon	0	0	0	0	0	0	0	0	0
g:Patulibac	0	0	0	0	0	0	0	0	0
g:Pelomon	0	0	0	0	0	0	0	0	0
g:Peptonip	0	0	0	0	0	0	0	0	0
g:Phycicocc	0	0	0	0	0	0	0	0.000981	0
g:Pigmenti	0	0	0	0.000129	0	0	0	0	0
g:Pleomorp	0	0	0	0	0	0	0	0	0
g:Prevotell	0	0.001055	0	0	0	0	0	0	0
g:Propionib	0	0	0.008755	0.000753	0.001678	0.001868	0	0.00136	0.001121
g:Propioniv	0	0	0	0	0	0	0	0	0

	0	0	0	0	0			0	0
	0	0	0	0	0			0	0
	0	0	0						
	0	0							
	0	0							
	0								
	0								
	0								
g:Pseudom	0	0	0	0	0.000682	0	0	0	0
g:Pseudono	0	0	0	0	0	0	0	0	0
g:Ralstonia	0.003085	0.005372	0.005276	0.003137	0.003142	0.00258	0	0.001898	0.001964
g:Rarobact	0	0	0	0	0	0	0.000952	0	0.000497
g:Rathayiba	0	0	0.000318	0.000242	0.000311	0	0	0	0
g:Rhodococ	0	0	0	0	0	0.000868	0.000972	0	0
g:Roseomo	0	0	0	0	0	0	0	0	0
g:Rothia	0	0	0	0	0	0	0	0	0
g:Rubellimi	0	0	0	0	0	0	0	0.000133	0
g:Schlegele	0	0	0	0	0	0	0	0	0
g:Sediminib	0	0	0	0	0	0	0	0	0
g:Serinicoc	0	0	0	0	0	0	0	0	0
g:Sphingom	0.066556	0.005909	0.075535	0.011124	0.007672	0.00977	0.009998	0.081244	0.03855
g:Sphingop						0	0		
g:Staphyloc		0.005085	0.002049	0.000722	0.002204	0.001471	0.004315	0.001009	
g:Stenotrop		0	0	0	0	0	0	0	0
g:Streptoco		0.002099	0	0	0	0	0	0	0.000576
g:Streptom		0	0	0	0.000212	0	0	0	0
g:Tepidimo		0	0	0	0	0	0	0	0
g:Terracocc		0	0	0	0	0	0	0	0
g:Thermus		0	0	0	0	0	0	0	0
g:Tsukamu	0	0	0	0	0	0	0	0	0
g:Veillonell	0	0	0	0	0	0	0	0	0
g:Virgibacil	0	0	0	0	0	0	0	0	0
g:Wautersi	0	0	0	0	0.000127	0	0	0	0
g:Yonghapa	0	0	0.000776	0	0	0	0	0	0.000703
g:Zhihengli	0	0	0	0	0	0	0	0	0
k:Bacteria	0	0	0.002919	0	0.000253	0.005643	0.013404	0	0.001337
o:Acidimicr	0	0	0	0	0	0	0	0	0
o:Actinomy	0	0.000538	0.001829	0.001009	0.002103	0.014941	0	0.000498	0.000798
o:Bacillales	0	0.012709	0	0	0	0	0.006688	0	0
o:Bacteroid	0	0	0	0	0	0	0	0	0
o:Burkhold	0	0	0	0	0	0	0	0	0
o:Caulobac	0	0	0	0	0	0	0	0	0
							0		
							0		
							0		
	0	0	0	0	0	0	0		0

	0	0	0	0	0			0	0
	0								0
	0								
	0								
	0								
	0								
	0								
	0								
o:Clostridia	0	0	0	0	0	0	0	0	0
o:CW040	0	0	0	0	0	0	0	0	0
o:Enteroba	0	0	0	0	0	0	0	0	0
o:Flavobac	0	0.000858	0	0	0	0	0	0	0
o:Gemellal	0	0	0	0	0.000306	0	0	0	0
o:JG30-KF-	0	0	0	0	0	0	0	0	0
o:Lactobac	0	0	0	0	0	0	0	0	0
o:Methylop	0	0	0	0	0	0	0	0	0
o:MLE1-12	0	0.00084	0	0	0	0	0	0	0.000792
o:mle1-48	0	0	0	0	0	0	0	0	0
o:Myxococ	0	0	0	0	0	0	0	0	0
o:Pseudom	0	0	0	0	0	0	0	0	0
o:RB41	0	0	0	0	0	0	0	0	0
o:Rhizobial	0	0	0	0.014076	0.011624	0	0	0.013735	0.011977
o:Rhodosp	0	0	0	0	0	0	0	0	0
o:Rickettsia	0	0	0	0	0	0	0	0	0
o:Sphingom	0	0.001455	0	0.0041	0.001062	0.001843	0.001443	0.003134	0
o:Streptoph	0	0	0	0	0	0	0	0	0.000498
p:Acidobac	0	0	0	0	0	0	0	0	0
p:FBP	0	0	0	0	0	0	0	0	0
p:SR1	0	0	0	0	0	0	0	0	0
s:Acinetoba	0	0	0	0	0	0	0	0	0
s:Acinetoba			0	0	0	0	0	0	0
s:Acinetoba			0	0	0	0	0	0	0
s:Anoxybac			0	0	0	0	0	0	0
s:Bacillus_f					0				
s:Bacillus_h						0	0		
s:Bacillus_s						0	0		
s:Bifidobac				0.000233	0.000505	0.000121	0	0.000207	0.000324
s:Bifidobac			0	0.000174	0	0.000175	0	0.000269	0
s:Brachyba			0	0	0	0	0	0	0
s:Bradyrhiz		0	0	0	0	0	0	0	0
	0	0							
	0	0							
	0	0							
	0	0	0	0		0	0	0	0

	0	0	0	0	0			0	0
	0	0	0	0	0			0	0
	0	0	0						
	0	0							
	0	0							
	0								
	0								
	0								
s:Brevibact		0	0	0	0	0	0	0.000874	0
s:Brevibact		0	0	0	0	0	0	0	0
s:Brevundim	0	0	0	0	0	0.000239	0	0	0
s:Coryneba	0	0	0.000486	0	0	0	0	0	0
s:Couchiop	0	0	0	0	0	0	0	0	0
s:Enterococ	0	0	0	0	0	0.000454	0	0	0
s:Haemoph	0	0	0	0	0	0	0	0	0
s:Janthinob	0	0	0	0	0	0	0	0	0
s:Lactobaci	0	0	0	0	0	0	0	0	0
s:Lactobaci	0	0	0	0	0	0	0	0	0
s:Luteibact	0	0	0	0	0	0	0	0	0
s:Macrococ	0	0	0	0	0	0	0	0	0
s:Methylob	0	0	0	0	0	0	0	0	0
s:Methylob	0.013336	0	0	0	0	0	0	0	0
s:Methylob	0.162277	0.204607	0.200551	0.242958	0.210575	0.228384	0.210667	0.236017	0.276206
s:Methylob	0	0	0	0	0.0061	0.009213	0	0	0
s:Microbac	0	0	0.001124	0	0	0	0	0	0
s:Microbac	0	0	0	0	0	0	0	0	0
s:Micrococ	0	0	0	0	0	0	0	0	0
s:Neisseria	0	0	0	0	0	0	0	0	0
s:Paracoccu	0	0	0	0	0	0	0	0	0
s:Prevotella	0	0	0	0	0	0	0	0	0
s:Propionib	0.054465	0.109294	0.105789	0.093643	0.086435	0.082031	0.078259	0.076252	0.07011
s:Propionib	0	0	0	0	0	0	0.001165	0	0
s:Pseudom	0	0	0	0	0	0	0	0	0
s:Pseudom	0	0	0	0	0	0	0	0	0
s:Rickettsia	0	0	0	0	0	0	0	0	0
s:Roseomo	0	0	0	0	0	0	0	0	0
s:Rothia_ae	0	0	0	0	0	0	0	0	0
s:Rothia_de	0	0	0	0	0	0	0	0	0
s:Rothia_m	0	0	0	0	0	0	0	0	0
s:Shinella_g	0	0	0	0	0	0	0	0	0
s:Sphingob	0	0	0	0	0	0	0	0	0
s:Sphingom	0.04978	0.073353	0	0.059838	0.066	0.059701	0.021402	0	0.003159
s:Sphingom	0	0	0	0	0	0	0	0	0
s:Sphingom	0	0	0	0	0	0	0	0	0
s:Staphyloc	0	0	0	0	0	0	0	0	0
							0		
							0		
							0		
	0	0	0	0	0	0	0		0

	0	0	0	0	0			0	0
	0								0
	0								
	0								
	0								
	0								
	0								
	0								
s:Staphyloc	0	0	0.000687	0	0	0		0	0.001152
s:Stenotrop	0	0	0	0	0	0		0	0
s:Streptoco	0	0	0	0	0	0		0	0
s:Variovora								0	

	0	0							
	0	0							
	0	0							
	0	0	0	0		0	0	0	0

s:Veillonell 0 0 0 0 0 0 0 0 0 s:Virgibacil 0 0 0 0 0 0 0 0 0

0	0			0	0	0	0	0	
0	0			0	0	0	0	0	
				0		0	0		
						0	0		
						0	0		
							0		
							0		
							0		

AHH21737	AHH21748	AHH21738	AHH21749	AHH21773	AHH21785	AHH21752	AHH21823	AHH21736	AHH21750
lower	lower	lower	lower	lower	lower	lower	lower	lower	lower
0	0	0	0	0	0	0	0	0	0
0	0	0	0	0	0	0	0	0	0
0	0	0	0	0	0	0	0	0	0
0	0	0	0	0	0	0	0	0	0.000424
0	0	0	0	0	0	0	0	0	0.000165
0	0	0	0	0	0	0	0	0	0
0	0	0	0	0	0	0	0	0	0
0	0	0	0	0	0	0	0	0.000659	0
0	0	0	0	0	0	0	0	0	0
0	0	0.018646	0.000245	0	0	0	0	0	0.001301
0	0	0	0	0	0	0	0	0	0
0	0	0.001118	0	0	0	0	0	0.00098	0
0	0	0	0	0	0	0	0	0	0.000302
0.00034	0	0.001312	0.001598	0	0.000788	0	0	0.001404	0.002332
0.009214	0.022302	0.005602	0.011769	0.004111	0.002707	0.00114	0.002464	0.017258	0.009823
0	0	0	0	0	0	0	0	0	0
0.00026	0	0	0	0	0	0	0	0	0
0.003958	0.001235	0.000919	0.001252	0.00192	0.000829	0.003364	0.003391	0.000945	0.000248
0	0	0	0	0	0	0	0	0	0
0	0	0	0	0	0	0	0	0	0
0.00174	0	0	0.001413	0.000637	0.002195	0	0	0	0.00212
0	0	0	0	0	0	0	0	0	0.00038
0	0	0.001265	0.001909	0.002241	0.010049	0	0	0	0.000283
0.000717	0	0	0.001478	0.002273	0.001593	0	0	0.001643	0.007594
0.000454	0	0	0	0	0	0	0	0	0.000271
0	0	0	0	0	0	0	0	0	0
0.009795	0	0.000605	0.002358	0.001635	0.001478	0	0.000843	0.001424	0.000589
0.000681	0	0.00091	0.000334	0	0	0	0	0	0.000574
0	0	0	0	0	0	0	0	0	0.000156
0	0	0	0	0	0	0	0	0	0
0	0	0	0	0	0	0	0	0	0
0	0	0	0	0.000797	0	0	0	0	0
0	0.001324	0	0.000457	0	0	0	0	0	0.000702
0	0	0	0	0	0	0	0	0	0
0	0	0	0	0	0	0	0	0	0
0	0.001702	0	0.001139	0.000284	0	0	0	0	0.000441
0	0	0	0	0	0	0	0	0	0
0	0	0	0	0	0	0	0	0	0.000982

0	0	0	0	0	0	0	0	0	0
0	0	0	0	0	0	0	0	0	0
0.005261	0	0.003702	0.009213	0.021445	0.017149	0.026568	0.016456	0.011859	0.012433
	0	0	0	0	0	0		0.000466	0
	0	0	0.001995	0.001277	0	0		0	0
	0.00115	0.005296	0.000773	0.001343	0.001169	0.001109		0.001276	0.000196
								0.000221	
		0.000425	0.00043						0
		0	0						0
0	0	0	0		0			0	0
0	0	0	0	0.000592	0.000655			0	0
0	0	0	0	0	0.000225			0	0
0	0	0	0	0.000564	0	0		0	0.000848
0	0	0	0	0	0	0		0	0
0	0	0	0	0	0	0		0.000947	0
0	0	0	0	0	0	0	0	0	0
0.000519	0.00139	0	0.001951	0.001996	0.00265	0	0.001735	0.000884	0.001028
0	0	0.000919	0	0	0	0	0	0	0
0	0	0	0	0	0	0	0	0	0
0	0	0	0	0	0	0	0	0	0
0	0	0	0	0	0.000349	0	0	0	0
0	0.001257	0	0.000442	0	0	0	0	0.000506	0
0	0	0.0005	0	0	0	0	0	0	0
0	0	0	0	0	0	0	0	0	0
0	0	0	0	0	0	0	0	0	0
0	0	0	0	0	0	0	0	0	0
0	0.000666	0.000482	0.00016	0.00049	0.000562	0	0	0	0
0	0	0	0	0	0	0	0	0	0
0.002749	0.163207	0.077222	0.13113	0.043436	0.064943	0.086835	0.056649	0.065305	0.116853
0	0	0	0	0	0	0	0	0	0.00106
0	0	0	0	0	0	0	0	0	0
0	0	0	0	0	0	0	0	0.00038	0
0	0	0	0	0	0	0	0	0	0
0	0	0	0	0	0.000562	0	0	0	0
0	0	0	0	0	0	0	0	0	0
0	0	0	0	0.000163	0	0	0	0	0
0	0	0	0	0	0	0	0	0	0
0	0	0	0	0	0	0	0	0	0
0	0	0	0	0	0	0	0	0	0
0	0	0	0	0	0	0	0	0	0
0	0	0	0	0	0	0	0	0	0
0	0	0	0	0	0	0	0	0	0
0	0	0.000453	0	0	0	0	0	0	0.000871
0.000632	0	0.00105	0.000895	0	0.000595	0	0	0.000693	0.000279
0							0		
0							0		
0							0		
0	0	0	0	0	0	0	0		0

0	0			0	0	0	0	0		
0	0			0	0	0	0	0		
				0		0	0			
						0	0			
						0	0			
							0			
							0			
							0			
0	0	0	0	0	0	0	0	0	0	
0	0	0	0	0.0007	0	0.000607	0	0	0	
0	0.000835	0	0.000805	0	0	0	0	0	0 0	0
		0	0	0	0.002283	0	0	0.003827	0	
0	0	0	0	0	0	0	0	0	0	
0	0	0	0	0	0	0	0.00098	0	0	
0	0	0.002368	0	0	0	0	0	0	0.000466	
0	0	0	0	0	0	0	0.000941	0	0	
0	0	0	0	0	0	0	0	0	0	
0.052155	0.029624	0.040769	0.036356	0.039746	0.03885	0.050532	0.039922	0.041968	0.031093	

0				0	0	0	0		
	0.000456	0.000485	0.000759					0.000722	0.000604
0.388162	0.357621	0.344501	0.348477	0.371729	0.395981	0.394026	0.363913	0.387579	0.390779
0	0	0	0	0	0	0	0	0	0
0.002214	0.004706	0.010546	0.005866	0.004666	0.003615	0.003193	0.003251	0.004329	0.002816
0	0	0	0	0	0	0	0	0	0
0	0	0	0	0	0	0	0	0	0
0	0	0	0	0	0	0	0	0	0
0	0	0	0	0	0	0	0	0	0
0	0	0	0	0	0	0	0	0	0
0	0	0	0	0	0	0	0	0	0
0	0	0	0.000484	0	0	0	0	0	0.000493
0	0	0	0	0	0	0	0	0	0
0	0	0	0	0	0	0	0	0	0
0	0.00523	0.001516	0.00695	0.026637	0.006928	0.001295	0.000443	0.001833	0.001996
0	0	0	0	0	0	0	0	0	0
0	0	0	0	0	0	0	0	0	0
0	0	0	0.00038	0	0	0	0	0	0
0	0	0	0	0	0	0	0	0	0
0	0	0	0	0	0	0	0	0	0
0	0	0	0.000415	0.000952	0	0	0	0	0.001198
0	0	0	0	0	0	0	0	0	0
0.036464	0.034779	0.035813	0.032829	0.039164	0.028347	0.03651	0.035088	0.029932	0.036746
0	0	0	0	0	0	0	0	0	0
0	0	0	0	0	0	0	0	0	0

0 0 0 0 0 0 0 0 0 0.002437 0 0

0 0 0 0.001683 0 0 0 0

0	0	0	0	0	0	0	0	0	0
0	0	0	0	0	0	0	0	0	0
0	0	0	0	0	0	0	0	0	0
0	0	0	0	0	0	0	0	0	0
0	0	0	0	0	0	0	0	0	0
0	0	0	0	0	0	0	0	0	0
0	0.000739	0	0.000601	0	0.00092	0.000755	0	0.00068	0.002312
0	0	0	0	0	0	0	0	0	0
0	0	0	0	0	0	0	0	0	0
0	0	0	0	0	0	0	0	0	0
0	0	0	0	0	0	0	0	0	0
0	0	0	0	0	0	0	0	0	0
0	0	0	0	0	0.000563	0	0	0	0
0	0	0	0	0	0	0	0	0	0
0	0	0	0	0	0	0	0	0	0
0.001447	0	0	0.000727	0.001551	0	0	0	0	0
0	0	0	0	0	0	0	0	0.000903	0
	0	0	0	0	0	0		0	0
0							0		
0							0		
0							0		
0	0	0	0	0	0	0	0		0

0	0	0	0	0	0	0	0	0	
0	0			0	0	0	0	0	
				0		0	0		
						0	0		
						0	0		
							0		
							0		
							0		
	0	0	0	0	0	0		0	0
	0	0	0	0	0	0		0	0
								0	
									0.000318
		0	0						0
0	0	0	0		0			0	0
0	0	0	0	0	0			0	0
0	0	0	0	0	0			0	0
0.029368	0.024197	0.030062	0.030719	0.035495	0.022263	0.003636		0.029916	0.014216
0	0	0	0.000688	0	0.000742	0		0	0
0	0	0	0	0	0	0		0	0
0	0	0	0	0	0	0	0	0	0
0	0	0	0	0	0	0	0	0	0
0	0	0	0.000757	0	0	0	0	0	0
0	0	0	0	0	0	0	0	0	0
0	0	0	0	0	0	0	0	0	0
0	0	0	0	0	0	0	0	0	0
0	0	0	0	0	0	0	0	0	0
0	0	0	0	0	0	0	0	0	0
0	0	0	0	0	0	0	0	0	0
0	0	0	0	0	0	0	0	0	0
0	0	0	0	0	0	0.002374	0	0	0
0	0	0	0	0	0	0	0	0	0
0	0	0	0	0	0	0	0	0	0
0	0	0.001208	0	0	0.000739	0.001216	0	0	0.000954
0	0	0	0	0	0	0	0	0	0
0	0	0	0	0	0	0	0	0	0
0	0.000545	0	0	0	0	0	0	0	0
0	0	0	0	0	0	0	0	0	0
0	0	0	0	0	0	0	0	0	0
0	0	0	0.000929	0	0	0	0	0	0
0	0	0	0	0	0	0	0	0	0
0	0	0	0	0	0	0	0	0	0.000318
0	0	0	0	0	0	0	0	0	0
0	0	0	0	0	0	0	0	0	0
0.00098	0.005055	0	0.022861	0.002839	0.002393	0	0	0.000499	0.00117
0	0	0	0	0	0	0	0	0	0

0	0	0	0	0	0	0	0	0	
						0	0		
						0	0		
						0	0		
						0	0		
							0		
							0		
							0		
0.001686	0	0.053355	0	0	0	0	0	0.001809	0.003123
0	0	0	0	0	0	0	0	0	0
0.002132	0	0.003018	0.002524	0.006024	0.004451	0.003179	0.002738	0.001652	0.005285
0	0	0	0	0	0	0	0	0	0
0	0	0.000681	0.000258	0	0	0.001773	0	0.0008	0
0	0	0	0	0.000419	0	0	0	0	0
0	0	0	0	0	0	0	0	0	0
0.000346	0	0	0	0	0	0	0	0	0
0	0	0	0	0	0	0	0	0	0
0	0.00094	0	0	0	0	0	0	0	0
0	0	0	0	0	0	0	0	0	0
0	0	0	0.000567	0	0	0	0	0	0
0.120932	0.004878	0.006741	0.034982	0.079299	0.008986				
0.045249	0.120211	0.085193	0.009227	0					
0.001567	0.013436	0.001343	0.009471	0.005058	0.006405			0.007	0.004503
0	0	0	0	0	0			0	0
0	0.000552	0	0.001653	0	0			0	0
0	0	0	0	0	0			0	0
0	0	0	0	0	0	0		0	0
0	0	0	0	0	0	0		0	0
0	0	0	0.000522	0	0	0		0.000349	0
0	0	0	0	0	0	0	0	0	0
0	0	0	0	0	0	0	0	0	0
0	0	0	0	0	0	0	0	0	0
0	0	0	0	0	0	0	0	0	0
0	0	0	0	0	0	0	0	0	0
0	0	0	0	0	0	0	0	0	0
0.000258	0	0	0	0	0.005657	0	0.002131	0	0.00056
0	0	0.000437	0	0	0	0	0	0	0.003333
0	0	0.001122	0	0.002711	0.013636	0	0	0.000205	0
0.001668	0.017115	0	0.000602	0	0.017659	0	0	0.003109	0.003822
0	0	0	0	0	0	0	0	0	0
0.001709	0	0	0	0	0	0	0	0	0
0	0	0	0	0	0	0	0	0	0
0	0	0	0	0	0	0	0	0	0
0	0	0	0	0	0	0	0	0	0
0	0	0	0	0	0	0	0	0	0
0							0		
0							0		
0							0		
0	0		0	0	0	0	0		

0	0	0	0	0	0	0	0	0	
0	0			0	0	0	0	0	
				0		0	0		
						0	0		
						0	0		
							0		
							0		
							0		
0	0	0	0	0	0	0	0	0	0
0	0	0	0	0	0	0	0	0	0
0	0	0	0	0	0	0	0	0	0
0	0	0	0	0	0	0	0	0	0
0	0	0	0	0	0	0	0	0	0
0	0	0	0.000763	0	0.000539	0	0	0	0.000238
0	0	0	0	0.000537	0	0	0	0	0
0	0	0	0	0	0	0	0	0.000425	0
0	0	0.015466	0	0	0	0	0	0	0
0	0	0	0	0	0	0	0	0	0
0.002109	0.000677	0	0.001448	0.00066	0	0.000809	0	0	0.011391
0	0	0	0	0	0	0	0	0	0
0	0	0	0	0	0	0	0	0.000374	0
0.000537	0	0.00273	0	0	0.000233	0	0.121216	0	0.002048
0	0	0	0	0	0	0	0	0	0
0	0	0	0	0	0	0	0	0	0
0	0	0	0	0	0	0	0	0	0
0	0	0	0	0	0	0	0	0	0
0	0	0	0.001341	0	0	0	0	0	0
	0	0	0	0	0	0		0	0
	0	0	0	0	0	0		0	0
	0	0	0	0	0	0		0	0
		0.004507						0	0.036937

0	0	0	0	0	0	0	0	0	
0	0			0	0	0	0	0	
				0		0	0		
						0	0		
						0	0		
							0		
							0		
							0		
									0
		0	0						0
0.000409	0.000278	0.000313	0.000172		0.000583			0.000406	0.000282
0	0	0.000437	0.000341	0.000487	0			0.000237	0
0	0	0	0	0	0.000957			0	0
0	0	0	0	0	0	0		0	0
0	0	0.001715	0	0	0	0		0	0
0	0	0	0.001535	0	0	0		0	0
0	0	0	0	0	0	0	0	0	0
0	0.000315	0	0	0	0	0	0.00075	0	0
0	0	0	0	0	0	0	0	0	0
0	0	0	0	0	0	0	0	0	0
0	0	0	0	0	0	0	0	0	0
0	0	0	0	0	0	0	0	0	0
0	0	0	0	0	0	0	0	0	0
0	0	0	0	0	0	0	0	0	0
0	0	0	0	0	0	0	0	0	0
0	0	0	0	0	0	0	0	0	0
0	0	0	0	0	0	0	0	0.005428	0
0	0	0	0	0	0	0	0	0	0
0.245785	0.235343	0.2222	0.220583	0.240315	0.222182	0.285608	0.184685	0.242183	0.208779
0	0	0	0	0	0	0	0	0	0
0	0	0	0	0	0.001097	0	0	0	0
0	0	0	0	0	0	0	0	0	0
0	0	0	0	0	0.000586	0	0	0	0
0	0	0	0	0	0	0	0	0	0
0	0	0	0	0	0	0	0	0	0
0	0	0	0	0	0	0	0	0	0
0.067606	0.0675	0.065243	0.060399	0.055806	0.052346	0.049394	0.042193	0.041792	0.041748
0	0	0	0	0	0	0	0	0	0
0	0	0	0	0	0	0	0	0	0
0	0	0	0	0	0	0	0	0	0
0	0	0	0	0	0	0	0	0	0
0	0	0	0.000464	0	0	0	0	0	0
0.000538	0	0	0	0	0	0	0	0	0
0	0.000947	0	0	0	0	0	0	0	0
0	0	0	0	0	0	0	0	0	0
0	0	0	0	0	0	0	0	0	0
0	0	0	0	0	0	0	0	0	0
0.00036	0	0.032997	0	0	0.050499	0.000829	0	0	0.018554
		0	0						
0	0	0	0	0	0	0	0	0	0

0.005248	0	0	0.000824	0	0.000376	0	0	0	0
0	0	0	0	0	0	0	0	0	0
0	0	0	0	0	0	0	0	0	0
0	0	0	0	0	0	0	0	0	0
0	0	0	0	0	0	0	0	0	0
0	0			0	0	0	0	0	0
0	0	0	0	0	0	0	0	0	0
0	0	0	0	0	0	0	0	0	0

		0	0						
0	0	0	0	0	0	0	0	0	0

AHH21797	AHH21819	AHH21822	AHH21799	AHH21786	AHH21740	AHH21763	AHH21821	AHH21775	AHH21800
lower	lower	lower	lower	lower	lower	lower	lower	lower	lower
0	0	0	0	0	0	0	0	0	0
0	0	0	0	0	0	0	0	0	0
0	0	0	0	0	0	0	0	0	0
0	0	0	0	0	0	0	0	0	0
0	0	0	0	0	0	0	0	0	0
0	0	0	0.001763	0.000487	0.001563	0	0	0	0
0	0	0	0	0	0	0	0.014409	0	0
0	0	0	0	0	0	0	0	0	0
0	0	0	0	0	0	0	0	0	0
0	0	0	0	0	0	0	0.001345	0	0
0	0	0	0	0	0	0	0	0	0
0	0	0	0	0	0	0	0	0	0
0.00039	0.0002	0	0	0	0	0.000568	0	0	0
0.000778	0.000324	0.002125	0.001097	0	0	0	0	0.002654	0.000383
0.003668	0.003124	0.004093	0.010343	0.003826	0.002848	0.003933	0.001527	0.002295	0.004865
0	0	0	0.000721	0	0	0	0	0	0
0	0	0	0	0	0	0	0	0	0
0	0.001222	0.004931	0.004867	0.001418	0.000691	0	0.002328	0.001686	0.002406
0	0	0	0	0	0	0	0	0	0
0	0	0	0	0.000584	0	0	0	0	0
0.001693	0.001743	0	0.001585	0	0.002157	0	0	0	0.000735
0	0	0	0	0	0	0	0	0	0
0.001313	0.001189	0.000191	0	0.000492	0.000325	0	0	0	0
0.000085	0.010236	0.011578	0.000368	0	0.000649	0	0.000788	0	0.000174
0	0	0	0	0	0	0	0.000351	0	0.001055
0	0.000491	0	0	0	0	0	0	0	0
0.000547	0.009483	0	0.000391	0.000813	0.002185	0	0.000381	0.001179	0.000505
0	0	0.000411	0	0	0.000846	0	0	0	0
0	0	0	0	0	0	0.000594	0	0	0
0	0	0	0	0	0	0	0	0	0
0	0	0	0	0	0.000232	0	0	0	0
0	0	0	0	0	0	0	0	0	0
0	0	0	0.001479	0	0	0	0	0	0.000284
0	0	0	0	0	0	0	0	0	0
0	0	0	0	0	0	0	0	0	0
0	0	0	0	0.004221	0	0	0	0	0
0	0	0	0	0	0	0	0	0	0
0	0	0	0	0	0	0	0	0	0
0	0	0	0	0	0	0	0	0	0
0	0	0	0	0	0	0	0	0	0

0.01824	0.009262	0.00906	0.019215	0.006188	0.005296	0.023243	0.020239	0.026833	0.017367
0	0	0	0	0	0	0	0	0	0
0.000845	0	0.000179	0.000565	0	0	0	0.00022	0	0
0.001443	0.002026			0.005101	0.000603	0.004613	0.002326	0.000757	0.005442

		0	0						
0	0	0	0	0	0	0	0	0	0

0	0	0		0	0	0	0	0	0
0	0	0		0	0	0	0	0	0
		0							0
									0
			0.000723						
			0						
0	0		0	0	0	0	0	0	
0.000809	0	0	0	0	0	0	0.000341	0	
0	0	0	0	0	0	0	0	0	0.000396
0	0.000591	0.001334	0.000466	0	0	0	0.000976	0	0.000111
0	0	0	0	0	0	0	0	0	0
0.003044	0	0.000214	0	0	0	0	0	0	0
0	0	0	0	0	0	0	0	0	0
0.003478	0.021942	0.001123	0.000498	0	0	0.000595	0.000979	0.000501	0.003153
0	0	0	0	0	0	0	0	0	0
0	0	0	0	0	0	0	0	0	0

0 0.000193 0 0 0 0 0 0 0 0 0 0

0 0 0 0 0.000403 0 0 0

0	0	0	0	0	0	0	0	0	0
0	0	0	0	0	0	0	0	0	0
0	0	0	0	0	0	0	0	0	0
0	0	0	0	0	0	0	0	0	0
0	0	0	0	0	0	0	0	0	0
0	0	0	0	0	0.00019	0	0.003057	0.001221	0
0	0	0	0	0	0	0	0	0	0
0.10254	0.048441	0.12115	0.055977	0.061097	0.006247	0.023834	0.086719	0.005649	0.115829
0.000648	0	0	0	0	0	0	0	0	0
0	0	0	0	0	0	0	0	0	0
0	0	0	0	0	0.000742	0	0	0	0
0	0	0	0	0	0	0	0	0	0
0	0	0	0	0	0	0	0	0	0
0	0	0	0	0	0	0	0	0	0
0	0	0	0	0	0	0	0	0	0
0	0	0	0	0	0	0	0	0	0
0	0	0	0	0	0.000349	0	0	0	0
0	0	0	0	0	0	0	0	0	0
0	0	0	0	0	0	0	0	0	0
0	0	0	0	0	0	0	0	0	0
0	0	0	0	0	0	0	0	0	0
0	0	0	0	0	0	0	0	0	0
0	0	0.000802	0	0	0.000548	0	0	0	0.001733
0	0	0	0	0	0	0	0	0	0
0.000715	0.001306	0	0	0.001203	0	0	0	0	0.000895
0	0.000161	0	0	0	0	0	0.001407	0	0
0.000906	0	0.000637	0	0	0.005633	0	0	0	0

0		0	0	0			0	0	
0 0 0 0.002808 0 0 0 0 0 0.000497 0 0 0 0 0 0 0 0 0									
0.007456	0.005318	0	0	0	0	0	0	0	0
0	0	0	0	0	0	0	0	0	0
0	0	0	0	0	0	0	0	0	0
0.032784	0.042099	0.03848	0.039167	0.042662	0.036848	0.046408	0.039225	0.043536	0.03854
	0.000282				0.001522	0.001467			0.001262
0.377211	0.382703	0.409621	0.4037	0.421372	0.409713	0.411315	0.383297	0.380347	0.386542
0	0	0	0	0	0	0	0	0	0
0.003927	0.033749	0.009744	0.001611	0.018039	0.001722	0.002148	0.002136	0.004872	0.002603
0	0	0	0	0	0	0	0	0	0
0	0	0	0	0	0	0	0	0	0
0	0	0	0	0	0	0	0	0	0
0	0	0	0	0	0	0	0	0	0
0	0	0	0	0	0	0	0	0	0
0	0	0	0	0	0	0	0	0	0
0	0	0	0	0	0	0	0	0	0
0	0	0	0	0	0	0	0	0	0
0	0	0	0	0	0	0	0	0	0
0.008259	0.006575	0.001598	0.005939	0.00122	0.004216	0	0.002799	0.000736	0
0	0	0	0	0	0	0	0	0	0
0	0	0	0.000926	0	0	0	0.000561	0	0
0	0	0	0	0	0	0	0	0	0
0	0	0	0	0	0	0	0	0	0
0	0	0	0	0	0	0	0	0	0
0	0	0.001215	0.00176	0.000512	0	0	0	0	0
0	0	0	0	0	0	0	0	0	0
0.036284	0.034605	0.039154	0.036261	0.0385	0.040478	0.036685	0.02642	0.029081	0.035589
0	0	0	0	0	0	0	0.000276	0	0
0	0	0	0	0	0	0	0	0	0
0	0	0	0	0	0	0	0	0	0
0.000663	0	0	0.001065	0	0	0	0	0	0
0	0	0	0	0	0	0	0	0	0
0	0	0	0	0	0	0	0	0	0
0	0	0	0	0	0	0	0	0	0
0	0	0	0	0	0	0	0	0	0
0	0	0.000647	0	0	0	0	0	0	0
0	0	0	0	0	0	0	0	0	0
0	0	0	0.000778	0.000547	0.000667	0.000819	0.0014	0.000393	0.000528
0	0	0	0	0	0	0	0	0	0
0	0	0	0	0	0	0	0	0	0
0	0	0	0	0	0	0	0	0	0
0	0	0	0	0	0	0.004324	0.001476	0.002273	0
0	0	0	0	0	0	0	0	0	0
		0	0						
0	0	0	0	0	0	0	0	0	0

0	0	0	0	0	0	0	0	0	0
0	0	0	0	0	0	0	0	0	0
		0							0
									0
0	0	0	0	0	0	0	0	0	0
0	0	0	0	0	0	0	0	0	0
0	0	0	0	0	0	0	0	0	0
0	0	0	0	0	0.000866	0	0	0	0
0	0	0	0	0	0	0	0	0	0
0	0	0	0	0	0	0	0	0	0
0	0	0	0	0	0	0	0	0	0
0	0			0	0	0	0	0	0
0	0		0	0	0	0	0	0	
0	0.00114	0	0	0	0	0	0	0	
0	0	0	0	0	0	0	0	0	0
0.044966	0.007638	0.036235	0.035731	0.007364	0.036044	0.008497	0.030518	0.044146	0.025772
0	0	0	0	0	0.000327	0	0	0	0
0	0	0	0	0	0.000561	0	0	0	0
0	0	0	0	0	0	0	0	0	0
0	0	0	0	0	0	0	0	0	0
0	0	0	0	0	0	0	0	0	0
0	0	0	0	0	0	0	0	0	0
0	0	0	0	0	0	0	0	0	0
0	0	0	0	0	0	0	0	0	0
0	0	0	0	0	0	0	0	0	0
0	0	0	0	0	0	0	0	0	0
0	0	0	0	0	0	0	0	0	0
0	0	0	0	0	0	0	0	0	0
0	0	0	0	0	0	0	0	0	0
0	0	0	0	0	0	0	0	0	0
0	0	0	0	0	0	0	0	0	0
0.001633	0	0.00074	0	0	0.000761	0	0	0	0
0	0	0	0	0.000193	0	0	0	0	0
0	0	0.00093	0	0	0	0	0	0	0
0	0	0	0	0	0	0	0	0	0
0	0	0	0	0	0	0	0	0	0
0	0	0	0	0	0	0	0	0	0
0	0	0	0	0	0	0	0	0	0
0	0	0	0	0	0	0	0	0	0
0	0	0	0	0	0	0	0	0	0
0	0	0	0	0	0	0	0	0	0
0	0	0	0	0	0	0	0	0	0
0.00546	0.006747	0	0.00063	0.00208	0.001311	0.003201	0	0	0
0	0	0	0	0	0	0	0	0	0
0	0	0.000877	0	0	0	0	0	0	0

0		0	0	0			0	0	
0	0	0	0	0	0	0	0	0	0
0.001734	0.001364	0.001252	0.005976	0.004366	0.00151	0.006341	0.004124	0.002251	0.002816
0	0.000192	0	0.002126	0	0	0	0	0	0.000534
0	0	0	0	0.001183	0	0	0	0	0.000695
0	0	0	0	0	0	0	0	0	0.000196
0	0	0	0	0	0	0	0	0	0
0	0	0	0	0	0	0	0	0	0
0	0	0	0	0.000494	0	0	0	0	0
0	0	0	0	0	0	0	0	0	0
0	0	0	0	0	0	0	0	0	0
0	0	0	0	0.00066	0	0	0	0	0
0.033016	0.071045	0.003308	0.007151	0.088068	0.134792	0.124457	0.010946	0.138962	0.00226

		0	0						
0	0	0	0	0	0	0	0	0	0

0	0	0	0	0	0	0	0	0	0
			0		0		0	0	
0.000411	0.005294	0.005364		0.00324		0.001426			0.006481
0	0	0	0	0	0	0	0	0	0
0	0	0	0	0	0	0	0	0	0
0	0	0	0	0	0	0	0	0	0
0	0	0	0	0	0	0	0	0	0
0	0	0	0	0	0	0	0	0	0
0	0	0	0	0	0	0	0	0	0
0	0	0	0	0	0	0	0	0	0
0	0	0	0	0	0	0	0	0	0
0	0	0	0	0	0	0	0	0	0
0	0	0	0	0	0	0	0	0	0
0	0	0	0	0	0	0	0	0	0
0	0	0	0	0	0	0	0	0	0
0	0.000604	0	0	0	0.014111	0	0	0.000953	0
0	0	0	0	0	0	0	0	0	0
0.001707	0.001288	0.012314	0.001628	0.000592	0	0.00266	0.000312	0	0.001445
0.003333	0	0.003698	0	0	0	0	0	0	0
0	0	0	0	0	0	0	0	0	0
0	0	0.002021	0	0	0	0	0	0	0
0	0	0	0	0	0	0	0	0	0
0	0	0	0	0	0	0	0	0	0
0	0	0	0	0	0	0	0	0	0
0	0	0	0	0	0	0	0	0	0
0	0	0	0	0	0.001067	0	0.002407	0	0
0	0	0	0	0	0	0	0	0	0
0	0	0	0	0	0	0	0	0	0
0	0	0	0	0	0	0	0	0	0
0	0	0	0	0	0	0	0	0	0
0	0	0	0	0	0	0	0.000784	0	0
0	0	0	0	0	0	0	0	0	0
0	0	0	0	0	0	0	0.001067	0	0
0	0	0	0	0	0.003844	0	0	0	0
0	0	0	0	0	0	0	0	0	0
0	0.001065	0.003055	0.001185	0.0129	0	0	0	0	0.012468
0	0	0	0	0	0	0	0	0	0
0	0	0	0	0	0	0	0	0	0
0.001636	0.001529	0	0.002184	0.001353	0.016352	0.003709	0.001537	0.003383	0.003525
0	0	0	0	0	0	0	0	0	0
0	0	0	0	0	0	0	0	0	0
0	0	0	0	0	0	0	0	0	0
0	0	0	0	0	0	0	0	0	0
0	0	0	0	0	0	0	0	0	0
0	0	0	0	0	0	0	0.002028	0	0
		0	0						
0	0	0	0	0	0	0	0	0	0

0	0	0	0	0	0	0	0	0	0
0	0.000432			0	0	0	0	0	0
0.000577	0.000304		0.000315	0.000687	0.000436	0.000445	0.000343	0.000718	
0	0	0.000606	0	0	0.00009	0	0	0	
0	0	0	0	0.001274	0.000639	0	0	0	0
0	0.000493	0	0	0	0	0	0	0	0 0 0
		0	0	0	0	0	0.002835	0	0
0	0	0	0	0	0	0	0	0	0
0	0	0	0	0	0	0	0	0	0
0	0	0	0	0	0	0	0	0	0
0	0	0	0	0	0	0	0	0	0
0	0	0	0	0	0	0	0	0	0
0	0	0	0	0	0	0	0	0	0
0	0	0	0	0	0	0	0	0	0
0	0	0	0	0	0	0	0	0	0
0	0	0	0	0	0	0	0	0	0
0	0	0	0	0	0	0	0	0	0
0	0	0	0	0	0	0	0	0	0
0	0.0015	0	0	0	0	0	0	0	0
0	0	0	0	0	0	0	0	0	0
0.259757	0.244803	0.236942	0.252379	0.244772	0.231243	0.264756	0.268551	0.290837	0.266929
0	0	0	0	0	0.007698	0.003315	0	0	0
0	0	0	0	0	0	0	0	0	0
0	0	0	0	0	0	0	0	0	0
0	0	0	0	0	0	0	0	0	0
0	0	0	0	0	0.000679	0	0	0	0
0	0	0	0	0	0	0	0	0	0
0	0	0	0	0	0	0	0	0	0
0.037545	0.036936	0.029215	0.02834	0.021911	0.020458	0.01985	0.018518	0.014738	0.011566
0	0	0	0	0	0	0	0	0	0
0	0	0	0	0	0	0	0	0	0
0	0	0	0	0	0	0	0	0	0
0	0	0.000167	0	0	0	0	0	0	0
0	0	0	0	0	0.000412	0	0	0	0
0	0	0	0	0	0	0	0	0	0
0	0	0.000274	0	0	0.000528	0	0	0	0
0	0	0	0	0	0	0	0	0	0
0	0	0	0	0	0	0	0	0	0
0	0	0	0	0	0	0	0	0	0
0	0.000359	0	0.068281	0.000586	0	0.000394	0.061045		0.044914
0	0	0.004716	0	0	0		0		0
0	0	0	0	0	0		0		0
0	0	0	0	0	0		0		0
0	0		0	0	0		0		0

0	0	0	0	0	0	0	0	0	0
0	0	0	0	0	0	0	0	0	0
		0							0
									0
0	0		0	0	0		0		0
	0		0	0	0		0		0
0	0	0	0	0	0	0	0	0	0
0	0	0	0	0	0	0	0	0	0

								0	
						0		0	
						0		0	
						0		0	
		0				0		0	
		0				0		0	
0		0				0		0	
0	0	0	0	0	0	0	0	0	0

AHH21764	AHH21776	AHH21788	AHH21803	AHH21729	AHH21743	AHH21731	AHH21790	AHH21815	AHH21802
lower	lower	lower	middle	middle	middle	middle	middle	middle	middle
0	0	0	0	0	0	0	0	0	0
0	0	0	0	0	0.000096	0	0	0	0
0	0	0	0	0	0.000473	0	0	0	0
0	0	0.000614	0	0	0	0	0	0	0
0	0	0	0	0	0	0	0	0	0
0	0	0	0	0	0.000072	0	0	0.000018	0
0	0	0	0	0	0	0	0	0.000333	0
0	0	0	0	0	0.000144	0	0	0	0
0	0	0	0	0	0	0	0	0	0
0	0	0	0	0	0	0	0	0	0
0.001213	0	0	0	0	0	0	0	0	0
0	0	0	0	0	0	0	0	0	0
0	0	0	0	0	0	0.00099	0	0	0
0.003414	0	0	0	0.0024	0	0	0	0	0
0.003564	0	0.002174	0.001279	0.001232	0.002698	0.000838	0.002226	0.000497	0.001348
0	0	0	0	0	0	0	0	0	0
0	0	0.000233	0.032487	0.016869	0.016516	0.024215	0.002762	0.015585	0.017231
0.002142	0.003947	0.000689	0	0.000207	0	0	0	0	0.000119
0	0	0	0	0	0	0	0	0	0
0	0	0	0	0	0	0	0	0	0
0	0	0.001053	0	0.00025	0.000232	0	0	0	0.000688
0	0	0	0	0	0	0	0	0	0
0	0	0	0.011847	0.00035	0.013576	0	0	0.001566	4.73E-05
0.000659	0.000838	0.004059	0.001011	0.001544	0.000212	0	0	0.001231	0.000308
0.000588	0	0	0	0	0	0	0	0	0.000117
0	0	0	0	0	0	0	0	0	0
0.000177		0	0.000701	0.00214	0	0.00044	0	0.000618	0.000312
0.000274	0	0	0	0.00153	0.005057	0.002403		0.003381	0.002128
								0.001123	0.002771
0	0	0	0	0	0	0	0	0	0
0	0	0	0	0	0	0	0	0	0
0	0	0	0	0	0	0	0	0	0
0	0	0	0	0	0.000245	0	0	0	0
0	0	0	0.000109	0	0.000434	0	0	0	7.84E-05
0	0	0	0	0	0	0	0	0	0
0	0	0	0	0	0	0	0	0	0
0	0	0	0	0	0	0	0	0	9.55E-05
0	0	0	0	0	0	0	0	0	0
						0		0	
						0		0	
						0		0	
						0		0	
		0				0		0	
		0				0		0	
0		0				0		0	
0	0	0	0	0	0	0	0	0	0

0	0		0	0	0	0	0	0	0
0	0	0	0	0	0		0		0
0	0	0	0	0	0		0		0
0	0	0	0	0	0		0		0
0.009801	0.012305	0.001806	0	0	0		0		0
0	0		0	0	0		0		0
0.000772	0		0	0.00036	0		0		0.000551
	0.000875		0.001764	0.000794	0.000703		0.000487		5.63E-05
					0.000183				0.000557
0				0.000807	0				0
0		0		0	0.001005	0			0
0		0		0	0	0			0
0		0		0	0	0			0
0	0	0.002017		0.000589	0.000496	0		0.000807	0.000138
0	0	0		0	0	0		0	0
0	0	0		0	0.000115	0	0.00006	0	0
0	0	0	0	0	0	0	0	0	0
0.000921	0.002405	0.000913	0.005652	0.000395	0.003517	0	0.00211	0.002013	0.001838
0	0	0	0	0	0	0	0	0	0
0	0	0	0	0	0	0	0	0	0
0	0	0	0	0	0	0	0	0	3.82E-05
0.001173	0	0	0	0	0	0	0	0	0
0	0.012021	0	0	0	0	0	0	0	0
0	0	0	0	0	0	0	0	0	0
0	0	0	0	0	0	0	0	0	2.61E-05
0	0	0	0	0	0	0	0	0	0
0	0	0	0	0	0	0	0	0	0
0	0.000918	0.000198	0	0	0	0	0	0	0
0	0	0	0	0	0	0	0	0	0
0.05892	0.142132	0.047183	0	0	0	0	0	0	0.00033
0	0	0.003021	0	0.000141	0	0	0	0.000572	0
0	0	0	0	0	0.018177	0	0	0	0
0	0	0	0	0	0.000466	0	0	0	0
0	0	0	0	0	0	0	0	0	0
0	0	0	0.000012	0	0	0	0	0	0
0	0	0	0	0	0	0	0	0	0
0	0	0	0	0	0.000076	0	0	0	0
0	0	0	0	0	0	0	0	0	0
0	0	0	0	0	0	0	0	0	0
0	0	0	0	0	0	0	0	0	6.74E-05
0	0	0	0	0	0	0	0	0	0
0	0	0	0	0.000144	0	0	0	0	0
			0	0		0	0	0	
			0	0		0	0	0	
			0	0		0	0	0	
			0	0		0	0	0	
		0	0	0		0	0	0	
0	0	0	0	0	0	0	0	0	0
0	0	0	0						
0	0	0	0	0	0	0	0	0	0

0	0	0	0	0		0	0	0	
	0	0	0			0	0	0	
	0		0				0	0	
	0		0				0	0	
	0		0				0	0	
			0				0		
			0				0		
			0						
0	0	0	0	0	0	0	0	0	0
0	0	0	0	0	0	0	0	0	0
0	0.001299	0	0	0.000275	0	0	0	0	0.000122
0	0	0	0	0	0	0	0	0	0
0	0	0	0.000061	0	0.000116	0	0	0	2.72E-05
0.000768	0	0	0	0	0	0	0	0	0
0.001176	0	0	0	0.000327	0	0	0	0	0.000209
0	0	0	0	0	0.000128	0	0	0	0
0	0	0	0	0	0	0	0	0	0
0	0	0	0	0	0	0	0	0	0
0.000408	0	0	0	0	0	0.000231	0	0	0
0	0	0	0	0	0	0	0	0	0
0.045007	0.049914	0.01914	0.00236	0.004126	0.005308	0.005563	0.004995	0.003114	0.004369
		0.000698							
0.424942	0.37382	0.461689	0.024228	0.057841	0.047029	0.050554	0.040661	0.032514	0.034226
0	0	0	0	0	0	0	0	0	0
0.003484	0.006005	0.002517	0.662074	0.78306	0.761027	0.826174	0.845747	0.854407	0.786169
0	0	0	0	0	0	0	0	0	0
0	0	0	0	0	0.00008	0	0	0	0
0	0	0	0	0	0.00013	0	0	0	0
0	0	0	0	0	0	0	0	0	0
0.000905	0	0.000467	0	0	0	0	0	0	0
0	0	0	0	0	0	0	0	0	0
0	0	0	0	0	0	0	0	0	0
0	0	0	0	0	0	0	0	0	0
0	0	0	0	0	0	0	0	0	0
0	0	0.000834	0.124798	0.000519	0.010952	0.000424	0.000647	0.008196	0.001529
0	0	0	0	0	0	0	0	0	0
0	0	0	0	0	0	0	0	0	0
0	0	0	0	0	0	0	0	0	0
0	0	0	0	0	0	0	0	0	0
0	0	0	0	0	0	0	0	0	0
0	0	0	0	0.000307	0	0	0	0	0.000667
0	0	0	0	0.000111	0	0	0	0	0
0.037779	0.03863	0.037009	0.001186	0.004463	0.004278	0.004026	0.002826	0.002683	0.003231
0	0	0	0	0	0	0	0	0	0
0	0	0	0	0	0	0	0	0	0
0	0	0	0	0	0	0	0	0	0
0	0	0	0.000151	0.000242	0.00031	0	0	0.000142	0.005084
0	0	0	0	0	0	0	0	0	0
0	0	0	0	0	0	0	0	0	0

0	0		0	0	0	0	0	0	0
0	0	0	0	0	0.000281	0	0	0	0
0	0	0	0	0	0	0	0	0	0
0	0	0	0	0	0.000446	0	0	0	0
0	0	0	0	0	0	0	0	0	0
0	0	0.000231	0	0	0	0	0	0	0
0	0	0	0	0	0.000558	0	0	0	0
0	0	0	0	0	0.000252	0	0	0	0
0	0	0	0	0	0	0	0	0	0
0	0	0	0	0	0	0	0	0	0
0	0	0	0	0	0	0.000968	0	0	0

0 0 0 0 0 0 0 0 0 0 0 0 0 0 0.000111

0	0	0			0				0
0	0	0			0.000672				0.000134
0	0	0			0				0
0	0				0				0
0	0				0.00064				0
	0								0

			0	0		0	0	0	
			0	0		0	0	0	
			0	0		0	0	0	
			0	0		0	0	0	
		0	0	0		0	0	0	

0 0 0 0 0 0 0 0 0 0

0 0 0 0

0	0	0	0	0	0	0	0	0	0

0	0	0	0	0	0	0	0	0	0
	0	0	0	0		0	0	0	
	0		0	0			0	0	
	0		0	0			0	0	
	0		0	0			0	0	
			0	0			0		
			0				0		
			0						
0					0				0
0		0			0	0			0
0		0			0	0			0
0		0			0	0			0
0.030143	0.035997	0.001818			0	0.000526		0	5.63E-05
0.000431	0	0		0.00036	0	0		0	0
0	0	0		0	0	0	0	0	0
0	0	0	0	0.000222	0	0	0	0	0
0	0	0	0	0	0	0	0	0	0
0	0	0	0	0	0	0	0	0	0
0	0	0	0	0.000037	0	0	0	0	0
0	0	0	0	0	0	0	0	0	0
0	0	0	0	0	0	0	0	0	0
0	0	0	0	0	0.000503	0	0	0	0
0	0	0	0	0	0	0	0	0	0
0	0	0	0	0	0	0	0	0	0
0	0	0	0	0	0	0	0	0	0.000221
0.00068	0	0	0	0.000295	0	0	0	0	0
0	0	0	0	0	0	0	0	0	0
0	0	0	0	0.000257	0	0	0	0	0
0	0	0.000632	0	0	0	0	0	0	0
0	0	0	0.000261	0	0.001075	0.000758	0	0	0
0	0	0	0	0	0	0	0	0	0.00058
0	0	0	0	0	0	0	0	0	0

0	0	0	0	0	0	0	0	0	2.82E-05	0	0
			0	0	0	0	0	0	0.000267		0

0	0	0	0.000032	0.000073	0	0	0	0	2.01E-05
0	0	0	0	0	0	0	0	0	0
0	0	0	0	0	0	0	0	0	0
			0	0			0	0	
			0	0			0	0	
			0	0			0	0	
			0	0			0	0	
			0	0			0	0	
			0	0			0	0	
			0	0	0		0	0	
			0	0	0		0	0	

0	0	0	0	0	0	0	0	0	0
	0		0	0		0			
	0		0	0					
	0		0	0					
	0		0	0					
			0	0					
			0						
			0						
0	0	0	0	0	0	0	0	0	0
0	0	0	0	0	0	0	0	0	0
0	0	0	0.002241	0.001093	0.007773	0.001326	0	0.001627	0.000911
0	0	0	0	0	0	0	0	0.000196	0
0	0	0	0	0.00103	0	0	0	0.000442	0.000156
0	0	0	0	0	0	0	0	0	0
0.002813	0	0	0.000319	0.00047	0.000421	0	0.00039	0.000615	0.000395
0.002316	0.000861	0	0	0	0	0	0	0	0
0.000459	0	0	0	0	0	0	0	0	0
0	0	0			0.000492	0			0
0	0	0			0	0			0
0	0	0			0	0			0
0	0	0			0	0			0
0	0	0			0	0			0
0	0	0			0	0			0
0	0	0				0			0
0.078678	0.017008	0.01448				0.000535			0.000487
0		0.007392			0.001477		0.00066	0.000265	0.000378
0		0			0	0.000804	0	0	0
0		0			0.001778	0	0	0	0
0		0			0	0	0	0	0
0	0	0			0	0	0	0	0.000124
0	0	0		0	0	0	0	0	0
0	0	0		0	0	0	0	0	0
0	0	0	0	0	0	0	0	0	0
0	0	0	0	0	0.000079	0	0	0	0
0	0	0	0	0	0	0	0	0	0
0	0	0	0	0	0	0	0	0	0
0	0	0	0	0	0	0	0	0	0
			0	0		0	0	0	
			0	0		0	0	0	
			0	0		0	0	0	
			0	0		0	0	0	
		0	0	0		0	0	0	
		0	0	0	0	0	0 0	0	0	0
0	0	0	0						
0	0	0	0	0	0	0	0	0	0

	0	0	0	0	0	0	0	0	0
	0	0	0	0		0	0	0	
	0		0	0			0	0	
	0		0	0			0	0	
	0		0	0			0	0	
			0	0			0		
			0				0		
			0						
0	0	0	0	0	0	0	0	0	0
0.003031	0.000617	0.012789	0.002611	0.000312	0.000205	0	0.00072	0	0.051308
0	0	0	0	0	0	0	0	0	0
0.001494	0.00849	0.000882	0.001517	0.000273	0	0	0	0.000197	0.000155
0	0	0.001887	0	0	0.005999	0	0.017797	0	6.03E-05
0	0	0	0	0	0	0	0	0	0
0	0	0.004041	0.010842	0.021579	0.001494	0	0.001226	0	0.023401

0 0 0 0 0 0 0 0 0 0.000338 0 0

0 0 0 0.000058 0 0 0 0

0	0	0	0	0	0.000304	0	0	0	0
0	0	0	0	0	0	0	0	0	0
0	0	0	0	0	0	0	0	0	0
0	0	0	0	0	0	0	0	0	0

0 0 0 0 0 0 0 0 0 2.01E-05 0 0

0.002176 0 0 0 0 0 0 0

0	0	0	0	0	0	0	0	0	0
0	0	0	0	0	0	0	0	0	0
0	0	0	0	0	0	0	0	0	0
0	0	0	0	0	0	0	0	0	0
0	0	0	0	0	0	0	0	0.000088	0
0	0	0	0	0	0	0	0	0	0

0.010884 0 0.009244 0 0 0 0 0 0 0 0 0

0 0 0 0 0 0 0 2.82E-05

0	0	0	0	0	0	0	0	0	0
0.00359	0.036521	0	0	0	0	0	0	0	0
0	0	0	0	0	0	0	0	0.000064	0
0	0	0			0				0
0	0	0			0				0

0 0 0 0.000092 0 0 0 0 0.000326

0	0				0				0
							0	0	
							0	0	
							0	0	
							0	0	
			0				0	0	

0 0 0 0 0 0 0

0	0	0	0		0	0	0	0	0

0	0	0	0	0	0	0	0	0	0
	0		0	0		0			
	0		0	0					
	0		0	0					
	0		0	0					
			0	0					
			0						
			0						
0	0				0				0
	0								0
0.006704									
0					0				0
0		0.000323			0	0.00176			0
0		0			0	0			0
0		0			0	0			0
0	0	0			0	0		0	0
0	0	0.000782		0.000195	0	0		0	0
0	0	0		0	0	0.000713	0	0	0
0	0	0	0	0	0	0	0	0	0
0	0	0	0	0	0.000102	0	0	0	0
0	0	0	0	0	0.0001	0	0	0	0
0	0	0	0	0	0	0	0	0	0
0	0	0	0	0	0.000424	0	0	0	0
0	0	0	0	0	0	0	0	0	0
0	0	0	0	0	0	0	0	0	0
0	0	0	0	0	0	0	0	0	0
0	0	0	0	0	0	0	0	0	0
0	0	0	0	0	0	0	0	0	0
0	0	0	0	0	0	0	0	0	6.54E-05
0	0	0	0	0	0	0	0	0	0
0.255456	0.251534	0.228695	0	0	0	0	0	0	7.74E-05
			0	0		0	0	0	
			0	0		0	0	0	
			0	0		0	0	0	
			0	0		0	0	0	
		0	0	0		0	0	0	

0 0 0 0 0 0 0 0 0 0

0 0 0 0

0	0	0	0	0	0	0	0	0	0

	0	0	0	0	0	0	0	0	0
	0	0	0	0		0	0	0	
	0		0	0			0	0	
	0		0	0			0	0	
	0		0	0			0	0	
			0	0			0		
			0				0		
			0						
0	0	0	0	0	0	0	0	0	0
0	0	0	0	0	0	0	0	0	0.00016
0	0	0	0	0	0	0	0	0	0
0	0	0	0	0	0	0	0	0	0
0	0	0	0	0	0	0	0	0	0
0	0	0	0	0	0	0	0	0	0
0	0	0	0	0	0	0	0	0	0
0.003644	0.003365	0.002278	0.109092	0.091219	0.081777	0.076214	0.07394	0.071124	0.057994
0	0	0.0003	0	0	0.000537	0	0	0	0
0	0	0	0	0	0	0	0	0	0
0	0	0	0	0	0	0	0	0	0
0	0	0	0	0	0	0	0	0	0
0	0	0	0	0	0	0	0	0	0.000156
0	0	0	0	0	0.000289	0	0	0	0
0	0	0	0	0	0	0	0	0	0
0	0	0	0	0	0.000286	0	0	0	0
0	0	0	0	0	0	0	0	0	0
0	0	0	0	0	0	0	0	0	0
0.000785	0.000501	0.125031	0	0	0	0			0
0	0	0	0	0	0	0			0
0	0	0	0	0	0	0			0
0	0	0	0.000395	0	0	0			0
0.001078	0	0		0	0	0			0
0	0	0		0	0	0			0
	0	0		0	0.000246	0			0
				0.000175					
							0	0	
							0	0	
							0	0	
							0	0	
			0				0	0	
			0	0	0 0	0	0 0		
0	0	0	0		0	0	0	0	0

0 0 0 0 0 0 0 0 0 0
0 0 0 0 0 0 0 0 0 0

AHH21769 AHH21791 AHH21770 AHH21792 AHH21742 AHH21768 AHH21801 AHH21755 AHH21745 AHH21780 middle middle middle middle middle middle middle middle middle middle

AHH21769 middle	AHH21791 middle	AHH21770 middle	AHH21792 middle	AHH21742 middle	AHH21768 middle	AHH21801 middle	AHH21755 middle	AHH21745 middle	AHH21780 middle
0	0	0	0	0	0	0	0	0	0
0	0	0	0	0	0	0	0	0.000091	0
0	0	0	0	0	0	0	0	0	0
0	0	0	0	0	0	0	0	0	0
0	0	0	0	0	0	0	0	0	0
0	0	0	0	0	0	0	0	0	0
0	0	0	0	0	0	0	0	0	0
0	0	0	0	0	0	0	0	0	0
0	0	0	0	0	0	0	0	0	8.51E-05
0	0	0	0	0	0	0	0	0	0
0	0	0	0	0	0	0	0	0	0
0	0	0	0	0	0	0	0	0	0
0	0	0	0	0	0	0	0	0	0
0.000159	0	0.001353	0.000525	0	0	0	0	0	0.000473
0.00038	0	0.000732	0.000936	0.000298	0.000772	0.002711	0	0.001303	0.00152
0.000123	0	0	0	0	0	0	0	0	0
0.018672	0.012658	0.031618	0.008146	0.018552	0.006125	0.026853	0.004555	0.007188	0.010788
0.000131	0	0	0.001453	0	0	0	0	0	0.002108
0	0	0	0	0	0	0	0	0	0
0	0	0	0	0	0	0	0	0	0
0	0.000606	0	0.000223	0.000514	0.000357	0.000317	0	0	0.00048
0	0	0	0	0	0	0	0	0	0

0 0.000437 0.00497 0.000043 0 0.000184 0.000377 0 0 0.000113 0 0.00079 0.000101 0 0.000102 0 0.000937 0 0.001941 0

0	0	0	0	0	0	0	0	0	0
0	0	0	0	0	0	0	0	0	0
0.000787	0.000358	0.001094	0.000108	0.000339	0	0.000169	0	0	0
0.001004	0.003959	0.002379	0.002567	0.002758	0.00145	0.004372	0.001928	0.002179	0.005385
0	0	0	0	0	0	0	0	0	0
0.000027	0.000248	0	0.000144	0	0	0	0	0.000086	0.003809
0	0	0	0	0	0	0	0	0	0
0	0	0	0	0	0	0	0	0	0
0	0	0	0.000109	0	0	0.000062	0	0	0
0	0	0	0	0	0	0	0	0	0
0	0	0	0	0	0	0	0	0	0
0	0	0	0	0	0	0	0	0	0
0	0	0	0	0	0	0	0	0	0
0	0	0	0	0		0		0	0
0	0	0	0	0		0		0	0
					0		0		
					0		0		
					0		0		
					0		0		
					0		0		
					0		0		
0					0		0	0	
0	0	0	0	0	0	0	0	0	0

			0		0		0		
			0		0		0		
			0		0		0		
			0		0		0		
			0		0		0		
							0		
							0		
							0		
0	0	0	0	0		0		0	0
0	0	0	0	0		0		0	0
0	0	0	0	0		0		0	0
0.00031	0	0	0.000658	0		0		0.000391	0
	0.002345	0.002021	0.001223	0.000134		0.000272			0.000354
0.000131	0.00111	0.000089		9.51E-05		0.000213		0.000128	0.000488
0	0	0		0		0		0	0
0	0	0		0		0.000143		0	0
0	0	0		0		0		0	0
0	0	0		0		0		0	0
0.00075	0	0.000245	0.000546	0	0.001058	0.000157		0.000209	0.000572
0	0	0	0	0	0	0		0	0
0	0	0	0	0	0	0		0	0
0	0	0	0	0	0	0	0	0	0
0.003224	0.000991	0.003	0	0	0.000391	0.003584	0	0	0.000253
0.000703	0.010878	0	0.010901	0	0.000349	0.000452	0	0.002438	0.016322
0	0	0	0	0	0	0	0	0.000116	0
0	0	0	0	0	0	0	0	0	0
0	0	0	0.000307	0	0	0	0	0	0
0	0	0	0	0	0	0	0	0	0
0	0	0	0	0	0	0	0	0	0
0	0	0	0	0	0	0	0	0	0

0 0 0 0.000328 0 0 0 0 0 0 0 0 0

0 0 0 0 0 0 0.00026

0	0	0	0	0	0	0	0	0	0
0	0	0	0	0	0	0	0	0	0
0	0	0	0	0	0	0.000092	0	0.000534	0
0	0	0	0	0	0	0.001454	0	0.000184	0
0	0	0	0	0	0	0.000107	0	0	0
0	0	0	0	0	0	0	0	0	0
0.00002	0	0	0	0	0	0	0	0	0
0	0	0	0	0	0	0	0	0	0
0	0	0	0	0	0	0	0	0	0
0	0	0	0	0	0	0	0	0	0
0	0	0	0	0	0	0	0	0	0
0	0	0	0	0	0	0	0	0	0
0.003534	0.001984	0.004093	0.001915	0	0.000182	0	0	0.002497	0.009965

0 0.00011 0 0 0 0 0 0 0 0 0 0 0 0

0 0 0 0 0 0.000433

0	0	0	0	0	0	0	0	0	0
0	0	0	0	0	0	0	0	0	0

0	0	0	0	0	0	0	0	0	0
0	0	0	0	0	0	0	0	0	0
0	0	0	0	0	0	0	0	0	0
0	0	0	0	9.91E-05	0	0	0	0	0
0	0	0	0	0	0	0	0	0	0
0	0	0	0	0	0	0	0	0	0
0	0	0	0	0	0	0	0	0	0
0	0	0	0	0	0	0	0	0.0001	0
0	0	0	0	0	0	0	0	0	0
0	0	0	0	0	0	0	0	0	0
0.000954	0.005727	0.001626	0.003496	0.006148	0.002731	0.004559	0.005646	0.002925	0.004195

					0		0		
					0		0		
					0		0		
					0		0		
					0		0		
					0		0		
0					0		0	0	
0	0	0	0	0	0	0	0	0	0

0	0		0	0		0	0	0	0
		0.00066			0.000454				
0.01144	0.055881	0.014716	0.042675	0.037424	0.026588	0.047891	0.037011	0.043735	0.039167
0	0	0	0	0	0	0	0	0	0
0.805926	0.820981	0.775505	0.854517	0.9036	0.933819	0.722881	0.928819	0.909741	0.805499
0	0	0	0	0	0	0	0	0	0
0	0	0	0	0	0	0	0	0	0
0	0	0	0	0	0	0	0	0	0
0	0	0	0	0	0	0	0	0	0
0	0	0	0	0	0	0	0	0	0
0	0	0	0	0	0	0	0	0	0
0	0	0	0	0	0	0	0	0	0.000169
0	0	0	0	0	0	0	0	0	0
0	0	0	0	0	0	0	0	0	0
0.00018	0.011136	0.10106	0.000141	0.000318	0.000455	0.000829	0.001691	0.000611	0.000676
0	0	0	0	0	0	0	0	0	0
0	0	0	0	0	0	0	0	0	0
0	0	0	0	0	0	0	0	0	0
0	0	0	0	0	0	0	0	0	0
0	0	0	0	0	0	0	0	0	0
0	0.000562	0	0	0	0	0.000069	0	0	0
0	0	0	0	0	0	0	0	0	0
0.001084	0.004562	0	0.004163	0.003967	0.002944	0.004318	0.004055	0.003157	0.004175
0	0	0	0	0	0	0	0	0	0
0	0	0	0	0	0	0	0	0	0
0	0	0	0	0	0	0	0	0	0
0.000122	0.000422	0.000083	0.000053	0.000124	0.000194	0.000161	0	0.000113	0.000145
0	0	0	0	0	0	0	0	0	0
0	0.000175	0	0	0	0	0	0	0	0
0	0	0	0	0	0	0	0	0	0
0	0	0	0	0	0	0	0	0	0
0	0	0	0	0	0	0	0	0	0
0	0	0	0	0	0	0	0	0	0
0	0	0	0	0	0	0.000052	0	0	0
0	0	0	0	0	0	0	0	0	0
0	0	0	0	0	0	0	0	0	0
0	0	0	0	0	0	0	0	0	0
0	0			0	0		0		0
0	0			0	0		0		0
0	0			0	0		0		0
0	0	0	0	0	0		0		0
0	0	0	0	0	0		0		0
0	0	0	0	0	0		0		0
0	0	0	0	0	0		0	0	0
0	0	0	0	0	0	0	0	0	0

0	0	0	0	0	0	0	0	0	0
							0 0	0 0	0
				0					0
				0					0
									0
0	0	0	0	0	0	0	0	0	0
0.00014	0	0	0	0	0	0	0	0	0
0	0	0	0	0	0	0	0	0	0
		0	0			0.000062		0	
		0	0			0		0	
		0.00011	0			0		0	
						0		0	
						0		0	
						0		0	
						0			
	0	0				0.000027		0	
0	0	0				0		0	
0	0	0				0		0	
0	0	0				0		0	
0	0	0.000142	0	0.000198	0	0.000152		0	
0	0	0	0.000685	0	0	0		0.000364	0.002029
0	0	0	0	0	0	0		0	0
0	0	0	0	0	0	0	0	0	0
0	0	0	0	0	0	0	0	0	0
0	0	0.000134	0	0	0	0	0	0	0
0	0	0	0	0	0	0	0	0	0
0	0	0	0	0	0	0	0	0	0
0	0	0	0	0	0	0	0	0	0
0	0	0	0	0	0	0	0	0	0
0	0	0	0	0	0	0	0	0	0
0	0	0	0	0	0	0	0	0	0
0	0	0	0	0	0	0	0	0	0
0	0	0	0	0	0	0	0	0	0
0	0	0	0	0	0	0	0	0	0
0	0	0	0	0	0	0	0	0	0
0	0	0	0	0	0	0	0	0	0
0	0	0.000213	0.000236	0	0	0	0	0	0
0	0			0	0		0		0
0	0			0	0		0		0
0	0			0	0		0		0
0	0	0	0	0	0		0		0
0	0	0	0	0	0		0		0
0	0	0	0	0	0		0		0
0	0	0	0	0	0		0	0	0
0	0	0	0	0	0	0	0	0	0

0	0	0	0	0	0	0	0	0	0
0			0	0	0		0		0
			0	0	0		0		0
			0	0	0		0		0
						0 0	0	0	0 0 0
							0		
							0		
0.000721	0	0.000495	0	0	0	0	0	0	0
0	0	0	0	0	0	0	0	0	0
0	0	0	0	0	0	0	0	0	0
0	0	0.000337	0	0	0	0	0	0	0
0	0	0	0	0	0	0	0	0	0
0	0	0	0.000266	0	0.000206	0	0	0	0
0	0	0	0	0	0	0.000203	0	0	0
0	0.001699	0	0	0	0	0	0	0.000437	0
0	0	0	0	0	0.000046	0	0	0	0
0	0.003361	0.000973	0	0	0	0	0	0	0
0	0	0	0	0	0	0	0	0	0
0.000569	0.007785	0	0.010492	0	0	0.000411	0	0.002098	0.051176
0	0	0	0	0	0	0	0	0	0
0	0.002162	0.001733	0.000523	0.001678	0.000322	0.000234	0	0.001067	0.002571
0	0	0	0	0	0	0	0	0	0
0	0	0	0	0	0	0	0	0	0
		0	0			0		0	
		0	0			0		0	
		0	0.000291			0		0	
						0		0	
						0		0	
						0.000036		0	
						0		0	
						0.000197		0.000774	
	0.002681	0.000534	0.000467		0.000512	0.051206	0.001877	0.00126	
0.007491	0	0.006206	0		0	0	0	0	
0	0	0	0		0	0.000424	0	0	
0	0			0	0		0		0
0	0			0	0		0		0
0	0			0	0		0		0
0	0	0	0	0	0		0		0
0	0	0	0	0	0		0		0
0	0	0	0	0	0		0		0
0	0	0	0	0	0		0		0
0	0	0	0	0	0		0		0

0	0	0	0	0	0	0	0	0	0
							0 0	0 0	0
				0					0
				0					0
									0
0	0	0	0		0	0.000074	0	0	
0	0.000455	0	0	0	0	0	0	0	
0	0	0	0	0	0	0	0	0	0
0	0	0	0	0	0	0	0	0	0
0	0	0	0	0	0	0	0	0	0
0	0	0	0	0	0	0	0	0	0
0	0	0	0	0	0	0	0	0	0
0	0	0	0	0	0	0	0	0	0
0	0	0	0	0	0	0	0	0	0
0	0	0	0	0	0	0	0	0	0
0.062628	0	0	0.016125	0	0.003144	0.020167	0	0	0.009309
0	0	0	0	0	0	0	0	0	0
0	0	0	0	0	0.000403	0.000246	0	0	0.000325
0	0	0	0	0	0	0.006883	0	0	0
0	0	0	0	0	0	0	0	0	0
0.012238	0	0.000283	0.000392	0.002712	0	0.078569	0	0	0.000156
0	0	0	0	0	0	0	0	0	0
0	0	0	0	0	0	0	0	0	0
0	0	0	0	0	0	0	0	0	0
0	0	0	0	0	0	0.000018	0	0	0
0	0	0	0.001897	0	0	0	0	0	0
0	0	0	0	0	0	0	0	0	0
0	0	0	0	0	0	0	0	0	0
0	0	0	0	0	0	0	0	0	0
0	0	0	0	0	0	0	0	0	0
0	0	0	0	0	0	0	0	0	0
0	0	0	0	0	0	0	0	0	0
0	0	0	0	0	0	0	0	0	0
0	0	0	0.007032	0	0	0	0	0	0.01157
0	0	0	0	0	0	0	0	0	0
0	0	0	0.00027	0	0	0.00006	0	0	0.000632
0	0	0	0	0	0	0	0	0	0
0	0			0	0		0		0
0	0			0	0		0		0
0	0			0	0		0		0
0	0	0	0	0	0		0		0
0	0	0	0	0	0		0		0
0	0	0	0	0	0		0		0
0	0	0	0	0	0		0	0	0
0	0	0	0	0	0	0	0	0	0

0	0	0	0	0	0	0	0	0	0
0			0	0	0		0		0
			0	0	0		0		0
			0	0	0		0		0
						0 0	0 0	0 0	0
							0		
							0		
0	0	0	0	0	0	0	0	0	0
0	0	0	0.000101	0	0	0	0	0	0
0	0.000518	0	0	0	0	0.00002	0	0	0
		0	0			0		0	
		0	0			0		0	
		0	0			0		0	
						0		0	
						0		0	
						0.000146		0	
						0			

0	0			0	0		0		0
0	0			0	0		0		0
0	0			0	0		0		0
0	0	0	0	0	0		0		0
0	0	0	0	0	0		0		0
0	0	0	0	0	0		0		0
0	0	0	0	0	0		0		0
0	0	0	0	0	0		0		0

0	0	0	0	0	0	0	0	0	0
0			0	0	0		0		0
			0	0	0		0		0
			0	0	0		0		0
					0	0	0	0	0 0 0
							0		
							0		
	0	0				0		0	
0	0	0				0		0	
0	0	0				0		0	
0	0	0				0		0	
0	0	0	0	0	0.000403	0		0	
0	0	0	0	0	0	0.000285		0	0
0	0	0	0	0	0	0		0	0
0	0	0	0	0	0	0	0	0	0
0	0	0	0	0	0	0	0	0	0
0	0	0	0	0	0	0	0	0	0
0	0	0	0	0	0	0	0	0	0
0	0	0	0	0	0	0	0	0	0
0	0	0	0.000227	0	0	0	0	0	0
0	0	0	0	0	0	0	0	0	0
0	0	0	0	0	0	0.000085	0	0	0
0	0	0	0	0	0	0	0	0	0
0	0	0	0	0	0	0	0	0	0
0	0	0	0	0	0	0	0	0	0
0	0	0	0	0	0	0	0	0	0
0	0	0	0	0	0	0	0	0	0
0	0	0	0	0	0	0	0	0	0
0	0	0	0	0	0	0	0	0	0
0	0	0	0	0	0	0	0	0	0
0	0	0	0	0	0	0	0	0	0
0	0	0	0	0	0	0	0	0	0
0	0	0	0	0	0	0	0	0.000201	0
0	0	0	0	0	0	0	0	0	0
0.050771	0.041695	0.029692	0.025655	0.020936	0.01691	0.016468	0.014418	0.013172	0.012002
0	0	0	0	0	0	0.000946	0	0	0
0	0	0	0	0	0	0	0	0	0
0	0.002699	0	0	0	0	0	0	0.000783	0
0	0	0	0	0	0	0	0	0	0
0.000029	0	0.000072	0.000052	0	0	0.000045	0	0	0
0	0.000167	0	0	0	0	0	0	0	0
0	0	0	0	0	0	0	0	0	0
0	0	0	0	0	0	0	0	0	0
0	0	0	0	0	0	0	0	0	0
0	0.000437	0	0.000112	0	0	0	0	0	0.001902
	0			0	0		0		
	0			0	0		0		
	0			0	0		0		
	0		0	0	0		0		0
	0		0	0	0		0		0
	0		0	0	0		0		0
0	0	0	0	0	0		0	0	0
0	0	0	0	0	0	0	0	0	0

0		0	0			0		0	0
0		0	0			0		0	0
0		0	0			0		0	0.000246
0		0				0		0	
0		0				0		0	
0.015753		0.013729				0		0.000175	
						0			
0	0	0	0	0	0	0	0	0	0
0	0.000419	0	0	0	0	0	0	0	0.000651

0				0	0			0	0
0				0	0			0	0
0				0	0			0	0
0				0	0			0	0
0			0	0	0			0	0
0			0	0	0			0	0
0			0	0	0			0	0
0	0	0	0	0	0	0	0	0	0

AHH21734 AHH21744 AHH21777 AHH21814 AHH21813 AHH21735 AHH21816 AHH21759 AHH21757
AHH21818 middle middle middle middle middle middle middle middle middle middle

0	0	0	0	0	0	0	0	0	0
0	0	0	0	0	0	0	0	0	0
0	0	0	0	0	0	0	0	0	0
0	0.000055	0	0	0	0	0	0	0	0
0	0	0	0	0	0	0	0	0	0
0	0	0	0	0	0	0	0	0	0.000066
0	0	0	0	0	0	0	0	0	0
0	0	0	0	0	0	0	0	0	0
0	0	0	0	0	0	0	0	0	0
0	0	0	0	0.000102	0	0	0	0	0
0	0	0	0	0	0	0	0	0	0
0	0	0	0	0	0	0	0	0	0
0	0	0	0	0	0	0	0	0	0
0	0	0.000495	0	0	0	0	0.000541	0.000238	0
0	0.000869	0.001397	0.000382	0.000596	0	0.000552	0.000624	0.000263	0
0	0	0	0	0	0	0	0	0	0
0.006194	0.006327	0.019044	0.004649	0.018235	0.010929	0.009069	0.012649	0.008575	0.011376
0	0	0	0	0	0	0.001064	0	0	0
0	0	0	0	0	0	0	0	0	0
0	0	0	0	0	0	0	0	0	0
0	0.000241	0.000375	0.000185	0	0	0.000241	0	0.000195	0
0	0	0.000069	0	0	0	0	0	0	0

0 0.000122 0.000144 0.000066 0 0.000203 0.000044
0.000139 0 0 0 0.000916 0.001086 0.000304 0.001135
0 0 0.000254 0.000214 0

0	0	0	0	0	0	0	0	0	0
0	0	0	0	0	0	0	0	0	0
0	0.000176	0	0.000219	0.000052	0	0	9.24E-05	0	0
0	0.001565	0.003758	0.00141	0.003164	0.001122	0.002484	0.001982	0.001198	0.000953
0	0	0	0	0	0	0	0	0	0
0	0	0	0	0	0	0.001151	0	0.000253	0
0	0	0	0	0	0	0	0	0	0
0	0	0	0	0	0	0	0	0	0
0	0	0	0	0	0	0	0.000128	0	0
0	0	0	0	0	0	0	0	0	0
0	0	0	0	0	0	0	0	0	0
0	0	0	0	0	0	0	0	0	0
0	0	0	0	0	0	0	0	0	0
	0	0	0			0	0		
	0	0	0			0	0		
	0	0	0			0	0		
	0	0	0.000077			0	0		

	0	0				0	0		
	0	0				0	0		
	0.000206	0.000251				0.000808	0.000285		

0				0	0			0	0
0				0	0			0	0
0				0	0			0	0
0				0	0			0	0
0			0	0	0			0	0
0			0	0	0			0	0
0			0	0	0			0	0
0	0	0	0	0	0	0	0	0	0

0								0	0
0								0	0
0								0	0
0								0	0
0								0	0
0									0
0									0
0									
	0.000036	0.000107	0.000116	0.000188	0.000051	0.000367	0.000391		
	0	0	0	0	0	0	0		
	0	0	0	0	0	0	0		
	0	0	0	0	0	0	0		
	0	0	0	0	0	0	0		
	0.000229	0.00068	0	0	0.000202	0.000441	0.000331	0.000151	
	0	0	0	0	0	0	0	0	
	0	0	0	0	0	0.000062	0	0	0
0	0	0	0	0	0	0	0	0	0

0.002738 0.000223 0.000179 0.000579 0.000145 0 0.000228 0.000455 0.002512 0 0 0.000824 0.000974

0.000309 0.000149 0.000274 0.00688 0.000556 0.002819 0

0	0	0	0	0	0	0	0	0	0
0	0	0	0.000362	0	0	0	0.000135	0	0
0	0	0	0	0	0	0	0	0.000226	0
0	0	0	0	0	0	0	0	0	0
0	0	0	0	0	0	0	0	0	0
0	0	0	0	0	0	0	0	0	0
0	0	0	0	0	0	0	0	0	0
0	0	0	0	0	0	0	0	0	0
0	0	0	0	0.000064	0	0	0	0	0
0	0	0	0	0	0	0.00007	0	0	0
0	0	0.000194	0	0	0	0	0.000381	0	0
0	0	0	0	0	0	0	0	0.000321	0
0	0	0	0	0.001443	0	0	0	0	0
0	0	0	0	0	0	0	0	0	0
0	0	0	0	0	0	0	0	0	0
0	0	0	0	0	0	0	0	0	0
0	0	0.000058	0	0	0	0	0	0	0
0	0	0	0	0	0	0	0	0	0
0									
0									
0									
0									
0									
0									
0									
0									

0	0	0	0		0	0	0	0	0
0	0	0	0	0	0	0	0	0	0
0	0	0	0	0	0	0	0	0	0
0	0	0	0	0.0004	0.020264	0.003665	0.004466	0.00094	0.020783
0	0	0	0	0	0	0	0	0	0
0	0	0	0	0	0	0	0	0	0
0	0	0	0	0	0	0	0	0	0
0	0	0	0	0	0	0	0	0	0
0	0	0	0	0	0	0	0	0	0
0	0	0	0	0	0	0	0	0	0
0	0.000022	0	0.00015	0	0	0	0	0	0
	0	0	0	0	0	0	0	0	0
	0	0	0	0	0	0.000327	0	0	0
	0.000046	0.00012	0	0.00002	0	0	0	0	0
	0	0	0	0	0.000348	0	0	0	0
	0	0	0	0	0	0	0	0	0
	0	0	0	0	0	0	0	0	0
	0	0	0	0	0	0	0	0	0
	0.002777	0.00552	0.004413	0.002982	0.000876	0.003032	0.001669	0.002236	0.000874
				0.000245					
0.005913	0.029629	0.061525	0.041736	0.032064	0.007733	0.030025	0.015647	0.019059	0.006609
0	0	0	0	0	0	0	0	0.000215	0
0.952606	0.940408	0.868482	0.89597	0.9219	0.941825	0.873425	0.924861	0.891779	0.944498
0	0	0	0	0	0	0	0	0	0
0	0	0	0	0	0	0	0	0	0
0	0	0	0	0	0	0	0	0	0
0	0	0	0	0	0	0	0	0	0
0	0	0	0	0	0	0	0	0	0
0	0	0	0	0	0	0	0	0	0
0	0	0	0	0	0	0	0	0	0
0	0	0	0	0	0	0	0	0	0
0	0	0	0	0	0	0	0	0	0
0	0.00105	0.008307	0.000353	0.000152	0.000124	0.000696	0.00016	0.000407	0.001821
0	0	0	0	0	0	0	0	0	0
0	0	0	0	0	0	0	0	0	0
0	0	0	0	0	0	0	0	0	0
0	0	0		0	0		0	0	0
0	0	0		0	0		0	0	0
0	0	0		0	0		0	0	0
0	0	0	0	0	0		0	0	0
0	0	0	0	0	0		0	0	0
0	0	0	0	0	0		0	0	0
0	0	0	0	0	0		0	0	0
0	0	0	0	0	0	0	0	0	0

0	0	0	0	0	0	0	0	0	0
0					0			0	0
0								0	0
0								0	0
0								0	0
0									0
0									0
0									
0	0	0	0	0	0	0	0	0	0
0	0	0	0	0.000089	0	0	0	0	0
0	0	0	0	0.000099	0.000122	0	0	0	0
0	0	0	0	0	0	0	0	0	0
0.000815	0.002436	0.006118	0.004019	0.002779	0.000917	0.002988	0.001374	0.001422	0.000488
0	0	0	0	0	0	0	0	0	0
0	0	0	0	0	0	0	0	0	0
0	0	0	0	0	0	0	0	0	0
0	0	0.000095	0.000691	0.000109	0.000127	0.000053	7.43E-05	0	0.000233
0	0	0	0.000069	0	0	0	0	0	0
0	0	0	0	0	0	0	0	0	0
0	0	0	0	0	0	0	0	0	0
0	0	0	0	0	0	0	0	0	0
0	0	0	0	0	0	0	0	0	0
0	0	0	0	0	0	0.000081	0	0	0
0	0	0	0	0	0	0	0	0	0
0	0	0	0	0	0	0	0	0	0
0	0	0	0	0	0	0	0	0	0
0	0	0	0	0	0	0	0	0	0
0	0	0	0	0	0	0.000099	0	0	0
0	0	0	0	0	0	0	0	0	0
0	0	0	0	0	0	0	0	0	0
			0			0			
			0			0.000152			
			0.000101			0			
						0			
						0			
						0			
						0			
0	0	0	0	0			0	0	0
0	0	0	0	0			0	0	0
0	0	0	0	0			0	0	0
0	0	0	0	0			0	0	0
0	0	0	0	0			0	0	0
0	0	0	0	0			0	0	0
0	0	0	0	0			0	0	0
0	0	0	0	0			0	0	0

0	0	0	0		0	0	0	0	0
	0	0.000144	0	0		0	0		
	0	0	0	0	0	0	0		
	0	0	0	0	0	0	0		
	0	0	0	0	0	0	0		
	0	0.00878	0.000046	0.000111	0	0	0	0	
	0	0	0	0	0	0.001198	0	0.000686	
	0	0	0	0	0	0	0	0	0
0	0	0	0	0	0	0	0	0	0
0	0	0	0	0	0	0	0	0	0
0	0	0	0	0	0	0	0	0	0
0	0	0	0	0.000022	0	0	0	0	0
0	0	0	0	0	0	0	0	0	0
0	0	0	0	0	0	0	0	0	0
0	0	0	0	0	0	0	0	0	0
0	0	0	0	0	0	0	0	0	0
0	0	0	0	0	0	0	0	0	0
0	0	0	0.000049	0	0	0	0	0	0
0	0	0	0	0	0	0	0	0	0
0	0	0	0	0	0	0	0	0	0
0	0	0	0	0	0	0.00021	0	0	0
0	0	0.000111	0	0.000079	0	0.00016	0	0	0
0	0	0	0	0.000677	0	0	0	0	0
0	0	0	0.000161	0	0	0	0	0	0
0	0	0	0	0	0	0	0	0	0
0	0	0	0	0	0	0	0	0	0
0	0.000169	0	0	0	0	0	0	0	0
0	0	0	0.000128	0	0	0	0	0	0
0	0	0	0	0	0	0	0	0	0
0	0	0	0	0	0	0	0	0	0
0	0	0	0	0	0	0	0	0	0
0	0	0	0	0	0	0	0	0	0
0	0.000351	0	0	0.000077	0.000173	0	0.0012	0	0
0	0	0		0	0		0	0	0
0	0	0		0	0		0	0	0
0	0	0		0	0		0	0	0
0	0	0	0	0	0		0	0	0
0	0	0	0	0	0		0	0	0
0	0	0	0	0	0		0	0	0
0	0	0	0	0	0		0	0	0
0	0	0	0	0	0	0	0	0	0

0	0	0	0	0	0	0	0	0	0
0					0			0	0
0								0	0
0								0	0
0								0	0
0									0
0									0
0									
0	0	0	0	0	0	0	0	0	0
0	0.000195	0.000287	0.000192	0	0	0.014485	0	0.004742	0
0	0	0	0	0	0	0	0	0	0
0	0.000326	0	0.000623	0.000307	0	0.000242	0	0.000611	0
0	0	0	0	0	0	0	0	0	0
0	0	0	0	0	0	0	0	0	0
					0	0			
					0	0			
					0	0			
					0	0			
					0	0			
					0	0			
					0	0			
					0.000365	0.00026			

0	0	0	0	0			0	0	0
0	0	0	0	0			0	0	0
0	0	0	0	0			0	0	0
0	0	0	0	0			0	0	0
0	0	0	0	0			0	0	0
0	0	0	0	0			0	0	0
0	0	0	0	0			0	0	0
0	0	0	0	0			0	0	0

0	0	0	0	0	0	0	0	0	0
0					0				
	0.000163	0.000554	0.000279	0.00184		0.000721	0.000229	0.046728	0.000401
0.019387	0	0	0.000167	0	0.005032	0	0.006678	0	0.005995
0	0	0	0	0.000416	0	0	0	0	0
0	0	0	0	0	0	0	0	0	0
0	0	0	0	0	0	0	0	0	0
0	0	0	0	0	0	0	0	0	0
0	0	0	0	0	0	0	0	0	0
0	0	0.000121	0	0	0	0	0	0	0
0	0	0	0	0	0	0	0	0	0
0	0	0	0	0	0	0.000129	0	0	0
0	0	0	0	0	0	0	0	0	0
0	0	0	0	0	0	0	0	0	0
0	0	0	0	0	0	0	0	0	0
0	0	0.000423	0.026701	0.000253	0	0.035603	0	0	0
0	0	0	0	0	0	0	0	0	0
0	0.000101	0	0.000289	0.000346	0	0	0	0	0
0	0	0	0.001829	0.001221	0	0	0	0.008689	0
0	0	0	0	0	0	0	0	0	0.00003
0	0.000086	0.000619	0.003685	0.000148	0.001519	0.00058	0.000413	0	0.001382
0	0	0	0	0	0	0	0	0	0
0	0	0	0	0	0	0	0	0	0
0	0	0	0	0	0	0	0	0	0
0	0	0	0	0	0	0	0	0	0
0	0	0	0	0	0	0	0	0	0
0	0	0	0	0	0	0	0	0	0
0	0	0	0	0	0	0	0	0	0
0	0	0	0	0	0	0	0	0	0
0	0	0	0	0	0	0	0	0	0
0	0.00002	0	0	0	0	0	0	0	0
0	0	0	0	0	0	0	0	0	0
0	0	0	0	0	0	0	0	0	0
0	0	0	0	0	0	0.000897	0	0	0
0	0	0	0	0	0	0	0	0	0
0	0	0.000289	0	0	0	0	0	0	0
0	0	0	0	0	0	0	0	0	0
0	0	0	0	0	0	0	0	0	0
0	0	0	0	0	0	0	0	0.000203	0
0	0	0	0	0	0	0	0	0	0
			0			0			
			0			0			
0	0	0		0	0		0	0	0
0	0	0		0	0		0	0	0
0	0	0		0	0		0	0	0
0	0	0	0	0	0		0	0	0
0	0	0	0	0	0		0	0	0
0	0	0	0	0	0		0	0	0
0	0	0	0	0	0		0	0	0
0	0	0	0	0	0	0	0	0	0

0	0	0	0	0	0	0	0	0	0
0					0			0	0
0								0	0
0								0	0
0								0	0
0									0
0									0
0									
			0			0			
						0			
						0			
						0			
						0			
	0	0	0	0		0	0		
	0	0	0	0	0	0	0		
	0	0	0	0	0	0	0		
	0.000079	0	0.00017	0	0	0	0		
	0	0	0	0	0	0.000381	0	0	
	0	0	0	0	0	0	0	0	
	0	0	0	0.000073	0	0.00027	0	0	0.000292
0	0	0	0	0	0	0	0	0	0
0	0	0	0	0	0	0	0	0	0
0	0	0	0	0	0	0	0	0	0
0	0	0	0	0	0	0	0	0	0
0	0	0	0	0	0	0	0	0	0
0	0	0	0	0	0	0	0	0	0
0	0	0	0	0	0	0	0	0	0
0	0	0	0	0	0	0	0	0	0
0	0	0	0	0	0	0	0	0	0
0	0	0	0	0	0	0	0	0	0
0	0	0	0	0	0	0	0	0	0
0	0	0	0	0	0	0	0	0	0
0.000863	0	0	0.000097	0	0.000662	0.000243	0.000781	0	0.000334
0	0	0	0	0	0	0	0	0	0
0	0	0	0	0	0	0	0	0	0
0	0	0	0	0	0	0	0	0	0
0	0	0	0	0	0	0	0	0	0
0	0	0	0	0	0	0	0	0	0
0	0	0	0	0	0	0	0	0	0
0	0	0	0	0	0	0	0	0	0
0.011484	0.010353	0.009452	0.009327	0.008265	0.007132	0.006277	0.006209	0.005081	0.003865
0	0	0	0	0	0	0	0	0	0
0	0	0	0	0	0	0	0	0	0
0	0	0	0	0	0	0	0	0	0
0	0	0	0	0	0	0	0	0	0
0	0	0	0.000096	0.000052	0	0	2.01E-05	0.000108	0
0	0		0	0				0	0
0	0		0	0				0	0
0	0		0	0				0	0
0	0		0	0				0	0
0	0		0	0	0			0	0
0	0		0	0	0			0	0
0	0	0	0	0	0		0	0	0
0	0	0	0	0	0	0	0	0	0

0	0	0	0	0	0	0	0	0	0
0	0	0	0	0	0	0	0	0	0
0	0	0	0	0	0	0	0	0	0
0	0	0	0	0	0	0	0	0	0
0	0	0	0	0	0	0.000338	0	0.000129	0
		0			0	0	0		
		0			0	0	0		
		0			0	0	0		
		0			0	0	0		
		0				0	0		
		0.00024				0	0.017277		
						0			
0	0	0	0	0	0	0	0	0	0
0	0	0	0	0	0	0	0	0	0

AHH21733 AHH21756 AHH21783 AHH21765 AHH21805 AHH21771 AHH21778 AHH21779 AHH21747
AHH21766 middle middle middle middle middle middle middle middle middle middle

0	0	0	0	0	0	0	0	0	0
0	0	0	0	0	0	0	0	0	0
0	0	0	0	0	0	0	0	0	0
0	0	0	0	0	0	0	0	0	0
0	0	0	0	0	0	0	0	0	0
0	0	0	0	0	0	0	0	0	0
0	0	0	0	0	0	0	0	0	0
0	0	0	0	0	0	0	0	0	0
0	0	0.002673	0	0	0	0	0	0	0
0.000138	0.000076	0	0	0	0	0	0	0	0
0	0	0	0	0	0	0	0	0	0
0	0	0	0	0	0	0	0	0	0
0	0	0	0	0	0	0	0	0	0
0.000491	0	0.001097	0	0	0	0	0	0	0.00086
0.001865	0.001087	0.000754	0.000676	0	0	0.000405	0	0	0.000491
0	0	0.001533	0	0	0	0	0	0	0
0.013004	0.003016	0.031713	0.002794	0.003535	0	0.009648	0.004662	0.00746	0.011528
0.001308	0	0.004512	0	0	0	0	0	0	0.000124
0	0	0	0	0	0	0	0	0	0
0	0	0	0	0	0	0	0	0	0
0.001621	0	0.000157	0	0	0	0.000688	0.00033	0	9.51E-05
0	0	0	0	0	0	0	0	0	0
0	0.000046	0	0	0.000148	0	0	0.000304	0	0.000266
8.21E-05	0	0.000486	0	0	0	0.001332	0.000195	0	0.000281
0	0	0.000047	0	0	0	0	0	0	0
0	0	0	0	0	0	0	0	0	0
0.000268	0	0.00066	0	0	0	0	0.00023	0	0.000052
0.003049	0.000994	0.004767	0.002858	0.000486	0	0.005319	0.005726	0.00087	0.003219
0	0	0	0	0	0	0	0	0	0
0.001638	0	0.001208	0	0	0	0	0	0	0
0	0	0	0	0	0	0	0	0	0
0	0	0	0	0	0	0	0	0	0
0	0	0	0	0	0	0	0	0	0
0	0	0	0	0	0	0	0	0	0
0	0	0.000386	0	0	0	0	0	0	0
0	0	0	0	0	0	0	0	0	0
0	0	0	0	0	0	0	0	0	0
0	0	0		0	0	0	0	0	0

0 0.000103 0 0 0 0 0 0 0 0 0 0 0 0.000042 0 0 0

			0						
			0						
			0						
			0	0					
			0	0	0				
			0	0	0				
0			0	0	0				
0	0	0	0	0	0	0	0	0	0

			0	0	0	0		0	
			0	0	0	0		0	
			0	0	0	0		0	
			0	0	0	0		0	
			0	0	0	0		0	
								0	
								0	
								0	
0.000281	0	0.000074			0.000974	0	0.000169	0	0
0	0	0				0	0	0	0
0.001232	0	0.001887				0	0	0	0
	0.000102	0.002975				0.000145	0.000039	0.000132	0.001362
0.000134	0.000027	0.00022					0.00018		0.000355
0	0	0					0		0
0	0	0					0		0
0	0	0					0		0
0	0	0					0		0
0.001017	0.001199	0.001103	0.001438	0	0	0	0		0.000938
0	0	0.00151	0	0	0	0	0		0
0	0.00011	0	0	0	0	0	0		0
0	0	0	0	0	0	0	0	0	0
0.000242	0.000165	0.000101	0	0	0.00015	0.000072	0.000233	0	0.001219
0.008436	0.000421	0.070083	0	0	0	0	0	0	0
0	0	0	0	0	0	0	0	0	0
0	0	0	0	0	0	0	0	0	0
0.000073	0	0.000488	0	0	0	0	0	0	0
0.000065	0	0	0	0	0	0	0	0	0.000191
0	0	0	0	0	0	0	0	0	0
0	0	0	0	0	0	0	0	0	0
0	0	0	0	0	0	0	0	0	0
0	0	0	0	0	0	0	0	0	0
0	0.000282	0	0	0	0	0	0	0	0
0	0	0	0	0	0	0	0	0	0
0.000276	0	0.000456	0	0	0	0.000403	0.000345	0	0
0	0	0.000069	0	0	0	0	0	0	0.000409
0	0	0	0	0	0	0	0	0	0
0	0	0.000068	0	0	0	0	0	0	0
0	0	0	0	0	0	0	0	0	0
0	0	0	0	0	0	0	0	0	0
0	0	0	0	0	0	0	0	0	0
0	0	0.000216	0	0	0	0	0	0	0
0	0	0	0	0	0	0	0	0	0
0	0	0	0	0	0	0	0	0	0
0.008407	0	0.006733	0	0.003881	0.002975	0.000239	0	0.018503	0
0	0	0	0	0	0	0	0	0	0
0.000287	0	0.00766	0	0	0	0	0	0	0
0	0	0	0	0	0	0	0	0	0
0	0	0	0	0	0	0	0	0	0
0.000101	0	0.000081	0	0	0	0.000083	0	0	0

	0		0	0	0	0	0	0	0
0	0	0	0	0	0	0	0	0	0
0	0	0	0	0	0	0	0	0	0
0	0	0	0	0	0	0	0	0	0
0.000852	0	0.001326	0	0	0	0	0	0	0
0	0.000055	0	0	0	0	0	0	0	0
0	0	0	0	0	0	0	0	0	0
0.000461	0	0	0	0	0	0	0	0	0
0	0	0	0	0	0	0	0	0	0
0	0	0.000021	0	0	0	0	0	0	0
0.006879	0.001953	0.003973	0.008962	0.000557	0.001313	0.005006	0.00707	0.001054	0.004905
0.000632		0							
0.053479	0.023135	0.034124	0.081581	0.005337	0.009799	0.050003	0.05693	0.009324	0.051815
0	0	0	0	0	0	0	0	0	0
0.788608	0.94436	0.631364	0.870539	0.961353	0.969841	0.901957	0.904492	0.954906	0.808282
0	0	0	0	0	0	0	0	0	0
0	0	0	0	0	0	0	0	0	0
0	0	0	0	0	0	0	0.000102	0	0
0	0	0	0	0	0	0	0	0	0
0	0	0	0	0	0	0	0	0	0
0	0	0.000044	0	0	0	0	0	0	0
0.0001	0	0.000091	0	0	0	0	0	0	0
0	0	0	0	0	0	0	0	0	0
0	0	0	0	0	0	0	0	0	0
0.000421	0.000848	0.000304	0.000487	0.000739	0.000413	0.000193	0.000713	0	0.000171
0	0	0	0	0	0	0	0	0	0
0	0	0	0	0	0	0	0	0	0
0	0	0	0	0	0	0.00031	0	0	0
0	0	0	0	0	0	0	0	0	0
0	0	0	0	0	0	0	0	0	0
0	0	0	0	0	0	0.000141	0	0	0
0.000055	0	0.005682	0	0	0	0	0	0	0
0.005119	0.001791	0.005973	0.007609	0.000621	0.000941	0.004496	0.005177	0.001049	0.003852
0	0	0	0	0	0	0	0	0	0
0	0	0	0	0	0	0	0	0	0
0	0	0	0	0	0	0	0	0	0
0	0.000088	0.00003	0	0.00016	0	0	0.000208	0	0
0	0	0	0	0	0	0	0	0	0
0	0	0	0	0	0	0	0	0	0
0	0	0	0	0	0	0	0	0	0
0	0	0	0	0	0	0	0	0	0
0	0	0	0	0	0	0	0	0	0
0	0	0	0	0	0	0	0	0	0
			0			0	0		0
			0			0	0		0
			0			0	0		0
			0	0		0	0		0
			0	0	0	0	0		0

0 0 0 0 0 0 0 0 0 0 0 0 0

0	0	0	0	0	0	0	0	0	0

			0	0	0	0		0	
			0	0	0	0		0	
			0	0	0	0		0	
			0	0	0	0		0	
			0	0	0	0		0	
								0	
								0	
								0	
0	0	0.001486	0	0	0	0	0	0	0
0	0	0	0	0	0	0	0	0	0
0	0	0	0	0	0	0	0	0	0
0	0	0.000041	0	0	0	0	0	0	0
0	0	0	0	0	0	0	0	0	0
0	0	0	0	0	0	0	0	0	0
0	0	0	0	0	0	0	0	0	0
0	0	0		0	0			0	
0	0	0		0	0			0	
0	0	0.000129		0.000289	0			0	
0	0	0			0			0	
0	0	0						0	
0	0	0						0	
	0.000192	0						0	

	0		0	0	0	0	0	0	0
			0	0	0	0		0	
			0	0	0	0		0	
			0	0	0	0		0	
			0	0	0	0		0	
								0	
								0	
								0	
0		0							
0	0	0					0		0
0	0	0.000139					0		0
0	0	0.000379					0		0
0	0	0.000259					0		0
0	0	0.000104	0	0.000129	0.000367	0.000093	0		0
0.002558	0	0.022815	0	0	0	0	0		0
0	0	0	0	0	0	0	0		0
0	0	0	0	0	0	0	0	0	0.000113
0	0	0	0	0	0	0	0.000125	0	0
0	0	0	0	0	0	0	0	0	0
0	0	0	0	0	0	0	0	0	0
0	0	0	0	0	0	0	0	0	0
0	0	0	0	0	0	0	0	0	0
0	0	0	0	0	0	0	0	0	0
0.000299	0	0	0	0	0	0	0	0	0
0	0	0	0	0	0	0	0	0	0
0	0	0	0	0	0	0	0	0	0
0	0	0	0	0	0	0.000085	0	0	0
0	0	0	0	0	0	0	0	0	0.000132
0.000644	0	0.026958	0	0	0.000822	0	0	0	0
0.000047	0	0	0	0	0	0	0	0	0
0	0	0.000327	0	0	0	0	0.000115	0.000578	0.000355
0	0	0	0	0.000247	0	0	0	0	0
0	0	0	0	0	0	0	0	0	0
	0		0			0	0		0
	0		0			0	0		0
	0		0			0	0		0
	0		0	0		0	0		0
	0		0	0		0	0		0
	0		0	0		0	0		0
0	0		0	0		0	0		0
0	0		0	0		0	0		0

	0		0	0	0	0	0	0	0
			0	0	0	0		0	
			0	0	0	0		0	
			0	0	0	0		0	
			0	0	0	0		0	
								0	
								0	
								0	
0	0	0	0	0	0	0	0	0	0
0	0	0.000126	0.000337	0	0	0	0	0	0.000314
8.31E-05	0	0	0	0	0	0	0	0	0
0	0	0.000154	0	0	0	0	0	0	0
0	0	0	0	0	0	0	0	0	0
0	0	0	0	0	0	0	0	0	0
0	0	0	0	0	0	0	0	0	0
0	0	0	0	0	0	0	0	0.00063	8.31E-05
0	0	0	0	0	0	0	0	0	0
0.037401	0	0.047779	0	0	0	0	0	0	0
0.000076	0	0.000923	0	0	0	0	0	0	0
0	0.000501	0	0.002617	0.002851	0	0.000263	0.000586	0	0.000219
0	0	0	0	0	0	0	0	0	0
0	0	0	0	0	0	0	0	0	0
0		0		0	0			0	
0		0		0	0			0	
0		0		0	0			0	
0		0			0			0	
0		0			0			0	
0		0.000109			0			0	
		0			0			0	
		0.000409			0.001918			0.000401	
0.000137		0.001293							
0.000257	0.001153	0.001735		0.000438		0.000274	0.000748		0.064419
0.000343	0	0.000532		0.001881	0.003161	0.000418	0	0.002649	0
	0	0	0			0	0		0
	0	0	0			0	0		0
	0	0	0			0	0		0
	0	0	0			0	0		0
	0	0	0			0	0		0
	0	0	0			0	0		0
0	0	0	0	0	0	0	0		0
0	0	0	0	0	0	0	0	0	0

	0		0	0	0	0	0	0	0
			0		0			0	
			0						
			0						
			0						
0	0	0		0	0	0	0	0	0.000191
0.000139	0	0		0	0	0	0	0	0
0	0	0	0.000174	0	0	0	0	0	0
0	0	0.000027	0	0	0	0	0	0	0
0	0	0	0	0	0	0	0	0	0
0	0	0.000071	0	0	0	0	0	0	0
0	0	0	0	0	0	0	0	0	0
0	0	0	0	0	0	0	0	0	0
0	0	0	0	0	0	0	0	0	0
0	0	0	0	0	0	0	0	0	0
0	0	0	0	0	0	0	0	0	0
0.036969	0	0.026517	0.008631	0	0	0.005126	0	0	0.021964
0	0	0	0	0	0	0	0	0	0
0.00026	0.000077	0.001129	0	0	0	0.000073	0.000516	0	0
0.000219	0.012641	0.000773	0	0	0	0.009964	0	0	0.009015
0	0	0	0	0	0	0	0	0	0
0.005591	0.001741	0.008769	0	0	0	0.001181	0.00891	0	0.010937
0	0	0.000021	0	0	0	0	0	0	0
0	0	0	0	0	0	0	0	0	0
0	0	0	0	0	0	0	0	0	0
0	0	0	0	0	0	0	0	0	0
0.000065	0	0	0.009176	0	0	0	0	0	0
0	0	0	0	0	0	0	0	0	0
0	0	0	0	0	0	0	0	0	0
0	0	0	0	0	0	0	0	0	0
0	0	0	0	0	0	0	0	0	0
0.000041	0	0.000089	0	0	0	0	0	0	0
0	0	0	0	0	0	0	0	0	0
0	0	0	0	0	0	0	0	0	0
0.003455	0	0.013175	0	0	0	0	0	0	0
0	0	0	0	0	0	0	0	0	0
0	0	0.001418	0	0	0	0	0	0	0
	0		0			0	0		0
	0		0			0	0		0
	0		0			0	0		0
	0		0	0		0	0		0
	0		0	0	0	0	0		0
	0		0	0	0	0	0		0
0	0		0	0	0	0	0		0
0	0	0	0	0	0	0	0	0	0

	0		0	0	0	0	0	0	0
			0	0	0	0		0	
			0	0	0	0		0	
			0	0	0	0		0	
			0	0	0	0		0	
								0	
								0	
								0	
0	0	0	0	0	0	0	0	0	0
0	0	0	0	0	0	0	0	0	0
0	0	0.000056	0	0	0	0	0	0	0
0	0	0	0	0	0	0.000086	5.81E-05	0	0
0		0		0	0			0	
0		0.00002		0	0			0	
0		0		0	0			0	
0		0			0			0	
0		0						0	
0.000104		0						0	
		0						0	
0		0							
0	0	0					0		0
0	0	0					0		0
0	0	0					0		0
0	0	0					0		0
0	0	0	0	0	0	0	0		0

0 0 0.008164 0 0 0 0 0 0 0 0 0

0 0 0 0 0 0.000188

0	0	0	0	0	0	0	0	0	0
0	0	0	0	0	0	0	0	0	0
0	0	0	0	0	0	0	0	0	0
0	0	0	0	0	0	0	0	0	0
0	0	0	0	0	0	0	0	0	0
0	0	0	0	0	0	0	0	0	0
0	0.000683	0	0	0	0	0	0	0	0
0	0	0	0	0	0	0	0	0	0
0	0	0.001579	0	0	0	0	0	0	0
0	0	0	0	0	0	0	0	0	0
	0	0	0			0	0		0
	0	0	0			0	0		0
	0	0	0			0	0		0
	0	0	0			0	0		0
	0	0	0			0	0		0
	0	0	0			0	0		0
0	0	0	0	0	0	0	0		0
0	0	0	0	0	0	0	0	0	0

	0		0	0	0	0	0	0	0
			0		0			0	
			0						
			0						
			0						
0	0	0	0	0	0	0	0	0	0
0.000652	0	0.000165	0	0	0	0	0	0	0
0	0	0.000483	0	0	0.004052	0	0	0.0007	0
0	0	0	0	0	0	0	0	0	0
0	0	0	0	0	0	0	0	0	0
0	0	0.000024	0	0	0	0	0	0	0
0	0.00011	0	0	0	0	0	0	0	0
0	0	0	0	0	0	0	0	0	0
0	0	0	0	0	0	0	0	0	0
0	0	0	0	0	0	0	0	0	0
0.003481	0.003044	0.002902	0.002121	0.001985	0.001895	0.00186	0.001836	0.001744	0.00158
0	0	0	0	0	0	0	0	0	0
0	0	0.000289	0	0	0	0	0	0	0
0.005666	0	0	0	0	0	0	0	0	0
0	0	0	0	0	0	0	0	0	0
0.000122	0	0	0	0	0	0.000093	0	0	7.31E-05
0	0	0	0	0	0	0	0	0	0
0	0	0	0	0	0	0	0	0	0
0	0	0	0	0	0	0	0	0	0
0	0	0	0	0	0	0	0	0	0
0.000764	0	0.00198	0	0	0	0	0	0	0
0				0	0			0	
0				0	0			0	
0				0	0			0	
0				0	0			0	
0				0	0			0	
0				0.015361	0.001379			0	
								0	

	0		0			0	0		0
	0		0			0	0		0
	0		0			0	0		0
	0		0	0		0	0		0
	0		0	0	0	0	0		0
	0		0	0	0	0	0		0
0	0		0	0	0	0	0		0
0	0	0	0	0	0	0	0	0	0

0	0	0	0	0	0	0	0	0	0
0	0	0	0	0	0	0	0	0	0

0 0 0 0 0 0 0 0 0 0 0 0 0

0 0

	0		0	0	0		0	0
	0		0	0	0		0	0

0 0 0 0 0 0 0 0 0 0

			0	0				0
			0	0				0

AHH21804 AHH21754 AHH21789 AHH21741 AHH21782 AHH21794 AHH21730 AHH21767
AHH21746 middle middle minmiddle minmiddle minmiddle minmiddle minmiddle minmiddle middle

0	0	0	0	0	0	0	0	0
0	0	0	0	0	0	0	0	0
0	0	0	0	0	0	0	0	0
0	0	0	0	0	0	0	0	0
0	0	0	0	0	0	0	0	0
0	0	0	0	0	0.001782	0	0	0.015028
0	0	0	0	0	0	0	0	0
0	0	0	0	0	0	0	0	0
0	0	0	0	0	0	0	0	0
0.000196	0	0	0	0	0	0	0.000097	0
0	0	0	0	0	0	0	0	0
0	0	0	0	0	0	0	0	0
0	0	0	0	0	0	0	0	0
0	0	0	0	0	0	0.000107	0.000099	0
0.000776	0.000143	0.001717	0.002114	0	0.002279	0.001284	0.002952	0.000228
0	0	0	0	0	0	0	0	0
0.010958	0.014492	0.011955	0.001567	0.018768	0.017947	0.030067	0.020865	0.028704
0	0	0	0	0	0	0	0	0
0	0.000711	0	0	0	0	0	0	0
0	0	0	0	0	0	0	0	0
0.000263	0.000427	0	0	0	0	0.000339	0.00021	0
0	0	0	0	0	0	0	0	0

0 0 0 0 0 0.000798 0 0.000587 0.002112 0 0
0.000608 0 0 0.000449 0.000456 0 0

0	0	0	0	0	0	0	0	0.000593
0	0	0	0	0	0	0	0	0
0	0	0	0	0	0	0.000064	0.000632	0
0.001415	0.001689	0.001276	0.000673	0	0.000705	0.001115	0.001508	0
0	0	0	0	0	0	0	0	0
0.000203	0	0	0	0	0	0.000649	0	0
0	0	0	0	0	0	0	0	0
0	0	0	0	0	0	0	0	0
0	0	0	0	0	0	0.000952	0	0
0	0	0	0	0	0	0	0	0
0	0	0	0	0	0	0	0	0
0	0	0	0	0	0	0	0.00067	0
0	0	0	0	0	0	0	0	0
0		0				0	0	
0		0				0	0	
0		0				0	0	

0		0.000582				0	0	
0						0	0	
0						0.000098	0	
0.000958						0.000075	0.003152	
						0.000032		
0						0		
0		0.000099				0		
0		0				0		
0		0				0		
0	0.000149	0				0.00041	0.000114	
0	0	0			0	0	0	
0	0	0.000181			0	0	0	
0	0	0	0	0	0	0	0	0
0	0.00274	0.010578	0.007735	0.016233	0.017676	0.011485	0.004934	0.003931
0.001862	0.000447	0	0	0	0.001369	0.000581	0	0
0	0	0	0	0	0	0	0	0
0	0	0	0	0	0	0	0	0
0	0	0	0	0	0	0	0	0
0	0	0	0	0	0	0	0	0
0	0	0	0	0	0	0	0	0
0	0	0	0	0	0	0	0	0
0	0	0	0	0	0	0	0	0
0	0	0	0	0	0	0	0	0
0	0	0	0	0	0	0	0	0
0	0	0	0	0	0	0	0	0
0	0	0	0	0	0	0	0	0
0	0.02025	0	0	0	0	0	0	0
0	0	0	0	0	0	0	0	0
0	0	0	0	0	0	0	0	0
0	0	0	0	0	0	0	0	0
0	0	0	0	0	0	0	0	0
0	0	0	0	0	0	0	0	0
0	0	0	0	0	0	0	0	0
0	0	0	0	0	0	0	0.000783	0
0	0	0	0	0	0	0	0	0
0.000632	0	0	0	0	0.004573	0	0	0.005964
0	0	0	0	0	0	0	0	0
0	0	0	0	0	0	0	0	0
0	0	0	0	0	0	0	0	0
0	0	0	0	0	0	0	0	0
0	0	0	0	0	0	0.000333	0	0
	0		0	0	0			0
	0		0	0	0			0
	0		0	0	0			0
	0		0	0	0			0
	0	0	0	0	0			0
	0	0	0	0	0			0

0 0 0 0 0 0 0 0 0 0 0 0 0

0 0

0	0	0	0		0	0	0	0	0	0		0	0	0
													0	0

	0		0	0	0		0	0
	0		0	0	0		0	0

				0	0	0	0	0		0	0	0	0	0

			0	0				0
			0	0				0
0	0	0	0	0	0	0	0	0.004485
0.000091	0	0	0	0	0	0	0	0
0	0	0	0	0	0	0	0	0
0	0	0	0	0	0	0	0	0
0	0	0	0	0	0	0	0	0
0	0	0	0	0	0	0	0.000085	0
0	0	0	0	0	0	0.000134	0	0
0	0	0	0	0	0	0	0	0
0	0	0	0	0	0	0	0	0
0.002269	0.002475	0.001632	0.001447	0.000972	0.000458	0.002416	0.002986	0.000443

0	0	0	0	0	0	0	0	0
0.021893	0.025928	0.013698	0.018054	0.004484	0.004943	0.018626	0.02668	0.004567
0	0	0	0	0	0	0	0	0
0.880006	0.490025	0.154669	0.336935	0.383079	0.379527	0.496324	0.609065	0.641309
0	0	0	0	0	0	0	0	0
0	0	0	0	0	0	0	0	0
0	0	0	0	0	0	0	0	0
0	0	0	0	0	0	0	0	0
0	0	0	0	0	0	0	0	0
0	0	0	0	0	0	0	0	0
0	0	0	0	0	0	0	0	0
0	0	0	0	0	0	0	0	0
0	0	0	0	0	0	0	0	0
0.000263	0	0.002484	0	0.003741	0.010803	0.00038	0.000029	0.000472
0	0	0	0	0	0	0	0	0
0	0	0	0	0	0	0	0	0
0	0	0	0	0	0	0	0	0
0	0	0	0	0	0	0	0	0
0	0	0	0	0	0	0	0	0
0	0.000117	0	0	0	0	0	0	0
0	0	0	0	0	0	0	0	0

			0		0		0	0		0	0	0
			0		0		0	0		0	0	0
			0		0		0	0		0	0	0
			0		0		0	0		0	0	0
			0		0		0	0		0	0	0
			0		0		0	0		0	0	0
0	0	0	0	0	0	0	0	0	0	0	0	0
									0	0	0	0

0 0 0 0 0 0 0 0 0 0 0 0 0 0

0 0 0

	0	0	0	0	0	0	0	0
	0	0	0	0	0	0	0	0
	0	0	0	0	0	0	0	0
			0	0	0			0
			0	0				0
			0	0				0
0.002004	0.00253	0.001628	0.001265	0.001138	0.000466	0.002104		0.000514
	0.002524							
							0	0
0	0	0	0	0	0	0		0
0	0	0	0	0	0	0	0	0
0.000055							0	0
0	0	0	0	0	0	0	0	0
0.001447	0	0	0	0	0	0	0	0
0	0	0	0	0	0	0	0	0
0	0	0	0	0	0	0	0	0
0	0	0	0	0	0	0	0	0
0	0	0	0	0	0	0		0
0	0	0	0	0	0	0		0
0	0	0	0	0	0	0	0	0
0	0	0	0	0	0	0	0	0
0	0	0	0	0	0	0	0	0
0	0	0	0	0	0	0	0	0
0	0	0	0	0	0	0	0	0
0	0	0	0	0	0	0	0	0
0	0	0	0	0	0	0	0	
0	0	0	0	0	0	0	0	
0								
0								
0								
0								
0								
0								
0								
0								
0								
	0	0	0	0	0	0	0	0
	0	0	0	0	0	0	0	0
	0	0	0	0	0	0	0	0
	0	0	0	0	0	0	0	0
	0	0	0	0	0	0	0	0
	0	0	0	0	0	0	0	0
	0	0	0	0	0	0	0	0
	0	0	0	0	0	0	0	0

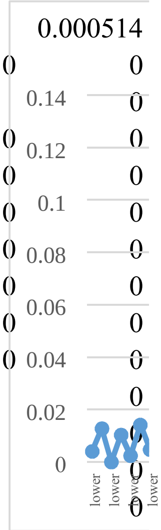

0	0	0	0	0	0	0	0	0 0
	0	0	0	0				
	0	0				0		
	0	0				0		
	0	0				0		
		0						
		0						
		0						
0								
0								

0	0	0.000194				0.000065	0	
0	0	0			0	0.000122	0	
0	0	0			0	0	0	
0	0	0	0	0	0	0	0	0
0	0	0	0	0	0	0	0	0
0	0	0	0	0	0	0	0	0
0	0	0	0	0	0	0	0	0
0	0	0	0	0	0	0	0	0
0	0	0	0	0	0	0.00007	0	0
0	0	0	0	0	0	0	0	0
0	0	0	0	0	0	0	0	0
0	0	0	0	0	0	0	0	0
0	0	0	0	0	0	0	0	0
0	0	0	0	0	0	0	0	0
0	0	0	0	0	0	0	0	0
0	0	0	0	0	0	0	0	0
0.000436	0	0	0	0	0	0	0	0
0	0	0	0	0	0	0.00041	0	0
0.000352	0	0	0	0	0	0	0	0
0	0	0	0	0	0	0	0	0
0	0	0	0	0	0	0	0	0
0	0	0	0	0	0	0	0	0
0	0	0	0	0	0	0	0.000048	0
0	0	0	0	0	0	0	0	0
0	0	0	0	0	0	0	0	0
0	0	0	0	0	0	0	0	0
0	0	0	0	0	0	0	0	0
	0	0	0	0	0	0	0	0
	0	0	0	0	0	0	0	0
	0	0	0	0	0	0	0	0
	0	0	0	0	0	0	0	0
	0	0	0	0	0	0	0	0
	0	0	0	0	0	0	0	0

0	0	0	0	0	0	0	0 0	0	0	0	0
								0	0	0	0

0 0 0 0 0 0 0 0 0 0 0 0 0 0
0 0 0

	0	0	0	0	0	0	0	0
	0	0	0	0	0	0	0	0
	0	0	0	0	0	0	0	0
			0	0	0			0
			0	0				0
			0	0				0
0.000167	0	0.002328	0.006515	0.005677	0.008081	0.005704	0.001274	0.002036
0	0	0	0	0	0	0	0	0
0.005916	0	0	0	0	0	0	0	0
0	0	0	0	0	0	0	0	0
0	0.000244	0.000185	0	0	0.00047	0.000076	0.000333	0
0	0	0	0	0	0	0.000026	0	0
0	0	0	0	0	0	0	0	0
0								
0								
0								
0								
0								
0								
0								
0.000342								
0	0.38383				0.000266		0.000168	
0	0			0.003829	0		0.000102	0.008654
0	0			0	0		0	0
0	0			0	0		0	0
0	0	0		0	0	0	0.000061	0
0	0	0		0	0	0	0	0
0	0	0		0	0	0	0	0
0	0	0	0	0	0	0	0	0
0	0	0	0	0	0	0	0	0
0	0	0	0	0	0	0	0	0
0	0	0	0	0	0	0	0	0
0	0	0	0	0	0	0	0	0
0	0	0	0	0	0	0	0	0
0.035083	0.00056	0	0	0	0	0.000017	0.000655	0
	0	0	0	0	0	0	0	0
	0	0	0	0	0	0	0	0
	0	0	0	0	0	0	0	0
	0	0	0	0	0	0	0	0
	0	0	0	0	0	0	0	0
	0	0	0	0	0	0	0	0
	0	0	0	0	0	0	0	0
	0	0	0	0	0	0	0	0

0 0 0 0 0 0 0 0 0 0
0 0 0 0
0 0 0
0 0 0
0 0 0
0
0
0

0	0	0	0	0	0	0	0	0
0	0.000276	0	0	0	0	0.000055	0.000512	0
0	0.040904	0	0	0	0	0	0	0
0	0	0	0	0	0	0	0	0
0.024977	0.004504	0	0.00033	0	0	0.001353	0.004778	0.003815
0	0	0	0	0	0	0	0	0
0	0	0	0	0	0	0	0	0
0	0	0	0	0	0	0	0	0
0	0	0	0	0	0	0	0	0
0	0.005389	0	0	0	0	0.010416	0	0
0	0	0	0	0	0	0	0	0
0	0	0	0	0	0	0	0	0
0	0	0	0	0	0	0	0	0
0	0	0	0	0	0	0.000104	0	0
0	0	0	0	0	0	0	0	0
0	0	0	0	0	0	0	0	0
0	0	0	0	0	0	0	0	0
0.000198	0	0	0	0	0	0	0	0
0	0	0	0	0	0	0	0	0
0	0	0	0	0	0	0	0	0
0	0	0	0	0	0	0	0	0
0	0	0	0	0	0	0	0	0
0	0	0	0	0	0	0	0	0
0	0	0	0	0	0	0	0.000046	0
0								
0								
0								
0								
0								
	0	0	0	0	0	0	0	0
	0	0	0	0	0	0	0	0
	0	0	0	0	0	0	0	0
	0	0	0	0	0	0	0	0
	0	0	0	0	0	0	0	0
	0	0	0	0	0	0	0	0

0 0 0 0 0 0 0 0 0 0 0 0 0
0 0 0 0

0 0 0 0 0 0 0 0 0 0 0 0 0 0

0 0 0

0	0	0	0	0	0	0	0
0	0	0	0	0	0	0	0
0	0	0	0	0	0	0	0
		0	0	0			0
		0	0				0
		0	0				0

0

0

0	0	0	0	0	0	0	0
0	0	0	0	0	0	0	0
0	0	0	0	0	0	0	0
0	0	0	0	0	0	0	0
0	0	0	0	0	0	0	0
0	0	0	0	0	0	0	0
0	0	0	0	0	0	0	0
0	0	0	0	0	0	0	0

0 0 0 0 0 0 0 0 0 0 0 0 0

0 0 0 0

	0	0	0	0	0	0	0	0
	0	0	0	0	0	0	0	0
	0	0	0	0	0	0	0	0
			0	0	0			0
			0	0				0
			0	0				0
0.003974								
0								
0								
0								
0	0.000218	0				0	0	
0.00029	0	0			0.000231	0	0	
0	0	0			0	0	0	
0	0	0	0	0	0	0	0	0
0	0	0	0	0	0	0	0	0
0	0	0	0	0	0	0	0	0
0	0	0	0	0	0	0	0	0
0	0	0	0	0	0	0	0	0
0	0	0	0	0	0	0	0	0
0	0	0	0	0	0	0	0	0
0	0	0	0	0	0	0	0	0
0	0	0	0	0	0	0	0	0
0	0	0	0	0	0	0	0	0
0	0	0	0	0	0	0	0	0
0	0	0	0	0	0	0	0	0
0.000266	0.000102	0	0	0.000461	0.000156	0	0	0.0001
0	0	0	0	0	0	0	0	0
0	0	0	0	0	0	0	0	0
0	0	0	0	0	0	0	0	0
0	0	0	0	0	0	0	0	0
0	0	0	0	0	0	0	0	0
0	0	0	0	0	0	0	0	0
0	0	0	0	0	0	0	0	0
0.001572	0.000815	0.796186	0.623365	0.557772	0.544422	0.413549	0.313993	0.269539
0	0	0	0	0	0	0	0	0.001018
0	0	0	0	0	0	0	0	0
0	0	0	0	0	0	0	0	0
0	0	0	0	0	0	0	0	0
0.000163	0	0	0	0	0	0	0	0
0	0	0	0	0	0	0	0	0
0	0	0	0	0	0	0	0	0
0	0	0	0	0	0	0	0	0
0.000585	0	0	0	0	0	0	0	0
0		0	0			0	0	
0		0	0			0	0	
0		0	0			0	0	
0		0	0			0	0	
0	0	0	0			0	0	
0	0	0	0			0	0	
0	0	0	0	0	0	0	0	0
0	0	0	0	0	0	0	0	0

0		0	0	0	0	0	0	0	0
		0			0	0			0
		0			0	0			0
		0			0	0			0
		0.001034			0.000271	0			0
					0	0			0
					0.003575	0.002598			0.006488
0		0	0	0	0	0	0	0	0
0.000441		0	0	0	0	0	0	0	0

0			0						
0			0						
0			0						
0			0						
0			0				0		
0			0				0		
0			0		0		0		
0	0	0	0	0	0	0	0	0	0

AHH21758 AHH21781 AHH21732 AHH21793 AHH21806 AHH21817 AHH21753 AHH21796 AHH21807
AHH21761 middle middle middle middle middle middle middle lower mini lower mini lower mini

0	0	0	0	0	0	0	0	0	0
0	0	0	0	0	0	0	0	0	0
0	0	0	0	0	0	0	0	0	0
0	0	0	0	0	0	0	0	0	0
0	0	0	0	0	0	0	0	0	0
0	0	0	0	0	0	0	0	0	0
0	0	0	0	0	0	0	0	0	0
0	0	0	0	0	0	0	0	0	0
0	0	0	0	0	0	0	0	0	0
0	0	0	0	0	0	0	0.000235	0	0
0	0	0	0	0	0	0	0	0	0
0	0	0	0	0	0	0	0.00076	0	0
0	0	0	0	0	0	0	0.000819	0.000352	0
0.000751	0	0	0	0	0	0.000242	0.000278	0.00474	0.00061
0	0	0.000778	0	0	0.000144	0.001637	0.004888	0.006453	0.005704
0	0	0	0	0	0	0	0	0	0
0.024514	0.04601	0.022348	0.009601	0.015655	0.02553	0.01352	0	0	0
0	0	0	0	0	0	0	0.000775	0.00099	0.000366
0	0	0	0	0	0	0	0	0	0
0	0	0	0	0	0	0	0	0	0
0	0	0.00077	0	0.001308	0.000186	0.000256	0.00043	0.000901	0.002434
0	0	0	0	0	0	0	0	0	0

0.000564 0 0.000074 0.000593 0.004616 0 0 0.012107 0
0.003986 0 0.000129 0.000441 0.000036 0 0 0.000384
0.000433 0.001005 0.007765

0	0	0	0	0	0	0	0	0	0
0	0	0	0	0	0	0	0	0	0

0 0.00028 0 0 0.000312 0.000118 0.000215 0.001481 0.001884 0.00226 0 0.000695 0.001616 0.00108
0.000895 0.001 0.0022 0 0 0

0	0	0	0	0	0	0	0	0	0
0	0	0	0	0	0	0	0	0	0
0	0	0	0	0	0	0	0	0	0
0	0	0	0	0	0	0	0	0	0
0	0	0.000702	0	0	0.001827	0	0	0	0.000225
0	0	0	0	0	0	0	0	0	0
0	0	0	0	0	0	0	0	0.000239	0
0	0	0	0	0.000186	0	0	0.00025	0	0.000465
0	0	0	0	0	0	0	0	0.000536	0
	0	0		0	0	0	0	0	0
	0	0		0	0	0	0	0	0
	0	0		0	0	0	0	0	0
	0	0		0	0	0	0.000928	0.006939	0.018354

	0	0		0	0	0		0	0
	0	0.000043		0	0.000526	0		0	0.000306
	0.000276	0.000996		0.000319		0.001091		0.005041	0.000805

0			0						
0			0						
0			0						
0			0						
0			0				0		
0			0				0		
0			0		0		0		
0	0	0	0	0	0	0	0	0	0

0	0		0	0	0			0	
0	0		0	0	0			0	
0								0	
0									
0									
0									
0									
0									
		0.00012				0.000412	0.000254		0.000265
		0				0	0.000331		0
	0	0	0	0	0	0	0		0
	0	0	0	0	0	0	0	0.000973	0
	0	0	0	0	0	0	0.004076	0	0
	0.001338	0.002418	0	0	0.001384	0	0	0.0009	0.001081
	0	0	0	0	0	0	0	0	0
	0	0	0	0	0	0	0	0	0.000143
0	0	0.000212	0	0	0	0	0	0	0
0.004279	0.004446	0.007407	0.007911	0.004013	0.001239	0.004524	0.009076	0.00123	0.01114
0	0.000109	0.000184	0.000541	0	0	0	0	0	0
0	0	0	0	0	0	0	0	0	0.000145
0	0	0	0	0	0	0	0	0	0
0	0	0	0	0	0	0	0	0.000574	0
0	0	0	0	0	0	0	0	0	0
0	0	0	0	0	0	0	0	0.00034	0
0	0	0	0	0	0	0	0	0	0
0	0	0	0	0	0	0	0	0	0
0	0	0	0	0	0	0	0	0	0
0	0	0	0	0	0	0	0	0	0
0	0	0	0	0	0	0	0	0	0
0	0	0	0	0	0	0	0.016907	0.036751	0.107867
0	0	0	0	0.000573	0	0	0	0	0.000253
0	0	0	0	0	0	0	0	0.003132	0.002029
0	0	0.000458	0	0	0	0	0	0	0
0	0	0	0	0	0	0	0	0	0
0	0	0	0	0	0	0	0	0	0
0	0	0	0	0	0	0	0	0	0
0	0	0	0	0	0	0	0	0	0
0	0	0	0	0	0	0	0	0	0
0	0	0	0	0	0	0	0	0	0
0.01367	0.008056	0.000242	0.004084	0.002994	0.019042	0	0	0	0
0	0	0	0	0	0	0	0	0	0
0	0	0	0	0	0	0	0	0	0
0	0	0	0	0	0	0	0	0	0
0	0	0	0	0	0	0	0.000119	0.000452	0
0	0	0.000046	0	0	0	0	0	0	0.000385

		0		0.000311			0.000591	0.000702	
0.00474	0.005675	0.02895	0.009263	0.007845	0.007406	0.044838	0.155495	0.277406	0.285679
0	0	0	0	0	0	0	0	0	0
0.671688	0.688483	0.72326	0.782965	0.740006	0.773869	0.784064	0.002041	0.01262	0.001042
0	0	0	0	0	0	0	0	0	0
0	0	0	0	0	0	0	0	0	0
0	0	0	0	0	0	0	0	0	0
0	0		0		0	0			0
0	0	0	0	0	0	0	0	0	0
0	0	0	0	0	0	0	0	0	0
0	0	0	0	0	0	0	0	0	0
0	0	0	0	0	0	0	0	0	0
0	0	0	0	0	0	0	0.000537	0	0
0	0	0	0	0	0	0	0	0	0
0.006421	0	0.000562	0.000471	0.035733	0.00028	0.000764	0.146604	0.003167	0.033188
0	0	0	0	0	0	0	0	0	0.000144
0	0	0	0	0	0	0	0.000216	0	0
0	0	0	0	0	0	0	0	0	0
0	0	0	0	0	0	0	0	0	0
0	0	0	0	0	0	0	0	0	0
0	0	0	0	0	0	0	0.001079	0	0
0	0	0	0	0	0	0	0	0	0
0.000528	0	0	0.000172	0	0	0	0	0	0
0	0	0	0	0	0	0	0	0	0
0	0	0.000101	0	0	0	0	0	0	0.000312
0	0	0	0	0	0	0	0.000376	0	0
0	0	0.000047	0	0	0	0	0.000214	0	0
0	0	0	0	0	0	0	0	0	0
0	0	0	0	0	0	0	0	0	0.000563
0	0	0	0	0	0	0	0	0	0.000026
0	0	0	0	0	0	0	0	0	0
0.000571	0.000566	0.002691	0.000898	0.000853	0.000913	0.004825	0.017801	0.023865	0.022506

0			0						
0	0		0			0			
0	0		0			0			
0	0		0			0			
0	0		0			0	0		
0	0		0			0	0		
0	0		0		0	0	0		
0	0	0	0	0	0	0	0	0	0

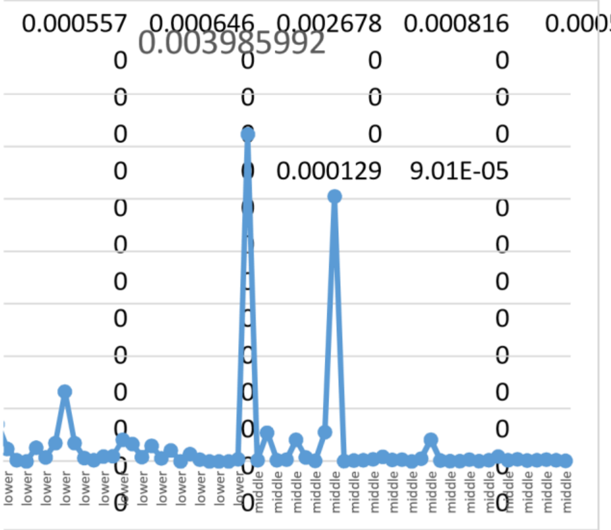

0	0		0	0	0	0		0	0
0	0		0	0	0			0	
0								0	
0									
0									
0									
0									
0									
0.000557	0.000646	0.002678	0.000816	0.00057	0.000459	0.004088	0.011194	0.02255	0.021381
0	0	0	0						
0	0	0	0						
0	0	0	0						
0	0	0.000129	9.01E-05						
0	0		0						
0	0		0						
0	0		0						
0	0		0						
0	0		0						
0	0		0						
0	0		0						
0	[illegible]		0						
0	0		0						
0	0		0						
0	0		0						
0	0		0						
0	0		0						
	0								
				0	0	0	0	0	0
				0	0	0	0	0	0
				0	0	0	0	0	0
				0	0	0	0.000135	0	0
		0		0	0	0	0	0	0
		0		0	0	0	0	0	0
		0		0	0	0	0	0	0
		0		0	0	0	0	0	0
		0		0	0	0	0	0	0
		0		0	0	0	0	0.001542	0.001062
		0		0	0	0	0	0	0.001991
		0		0	0	0	0	0	0
		0		0	0	0	0	0	0
		0		0	0	0	0	0	0.000222
		0		0	0	0	0	0	0.000306
		0		0	0	0	0	0	0
0	0					0			
0	0					0			
0	0					0			
0	0					0			
0	0					0			
0	0					0			
0	0					0			

0	0		0		0	0			0
		0		0	0	0	0	0	0
		0		0	0	0	0	0	0
		0		0	0		0	0	0
		0		0	0		0.00076	0	0
		0		0	0		0	0	0
		0		0	0			0	0
		0		0	0			0	0
		0		0				0	0
		0					0.000584		
		0				0	0		0
	0	0	0	0	0	0	0		0
	0	0	0	0	0	0	0	0	0
	0	0	0	0	0	0	0	0	0
	0	0.000141	0	0	0	0	0.012953	0.024017	0.028065
	0	0	0	0	0	0	0	0	0.00021
	0	0	0	0	0	0	0	0	0
0	0	0	0	0	0	0	0.000193	0	0
0	0	0	0	0	0	0	0	0	0
0	0	0	0	0	0	0	0.000298	0	0
0	0	0	0	0	0	0	0	0	0
0	0	0	0	0	0	0	0	0	0
0	0	0	0	0	0	0	0	0	0
0	0	0	0	0	0	0	0	0	0
0	0	0	0	0	0	0	0	0	0
0	0	0	0	0	0	0	0	0	0
0	0	0	0	0	0	0	0	0	0
0	0	0	0	0	0	0	0	0	0
0	0	0	0	0	0	0	0	0	0
0	0	0	0	0	0	0	0	0	0
0	0	0	0	0	0	0	0.000242	0	0.000435
0	0	0	0	0	0	0	0	0	0.000101
0	0	0	0	0	0	0	0.000397	0	0
0	0	0	0	0	0	0	0	0	0
0	0	0	0	0	0	0	0	0	0
0	0	0	0	0	0	0	0	0	0
0	0	0	0	0	0	0	0	0	0
0	0	0	0	0	0	0	0	0	0.000319
0			0						
0	0		0			0			
0	0		0			0			
0	0		0			0			
0	0		0			0	0		
0	0		0			0	0		
0	0		0		0	0	0		
0	0	0	0	0	0	0	0	0	0

0	0		0	0	0	0		0	0
0	0		0	0	0			0	
0								0	
0									
0									
0									
0									
0									
0	0	0	0	0	0	0	0	0	0
0	0	0	0	0	0	0	0	0	0
0	0	0	0	0	0	0	0	0	0
0.001156	0.003905	0.006716	0.001298	0.003919	0.004736	0.001501	0.002764	0.003686	0.003204
					0				
					0				
					0				
					0				
			0	0	0				
		0	0	0	0				
	0	0	0	0	0				
		0	6.91E-05	0	0				
		0	0	0	0				
		0	0	0	0				
		0	0	0	0				
		0	0	0	0				
		0	0	0	0				
		0.000138	0	0	0				
0	0	0	0	0		0	0	0	0
0	0	0.000168	0	0		0	0	0	0
0	0	0	0	0		0	0	0	0
0.001005	0	0.000234	0.000356	0.001139		0.000818	0.001271	0.002214	0.001015
0	0	0.000207				0.000435	0.000426	0	0
0	0					0	0	0	0

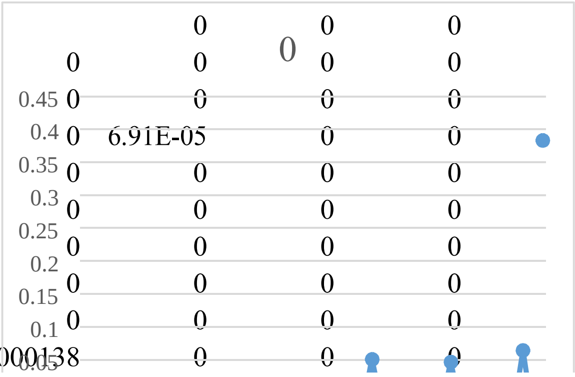

00 0 0.001162 0 0 0 0

							0	0	0
							0	0	0
							0	0	0
							0	0	0
							0	0	0
							0.027542	0.061901	0.033356
0	0					0			
0	0					0			
0	0					0			
0	0					0			
0	0					0			
0	0					0			
0	0					0			

0	0					0		0	0
0	0							0	
								0	
0	0	0	0	0	0	0	0	0	0
0	0	0	0	0	0	0	0	0	0
0	0	0	0	0	0	0	0	0	0
0	0	0	0	0	0	0	0	0	0.00031
0	0	0	0	0	0	0	0	0	0
0	0	0	0	0	0	0	0	0	0
0	0	0	0	0	0	0	0	0	0
0	0	0	0	0	0	0	0	0	0
0	0	0	0	0	0	0	0	0	0
0	0	0.000936	0	0	0	0	0	0	0
0	0	0.001404	0	0	0.00869	0.003166	0	0	0
0	0	0	0	0	0	0	0	0	0
0.002187	0	0.002632	0.00038	0.000923	0	0	0.000697	0	0.005355
0	0	0	0	0	0	0.002704	0.020177	0	0.002275
0	0	0	0	0	0	0	0	0	0
0.004275	0.000299	0.000211	0	0.005995	0.016834	0.001914	0.000154	0	0
0	0	0	0	0	0	0	0	0	0
0	0	0	0	0	0	0	0	0	0
0	0	0	0	0	0	0	0	0	0
0	0	0	0	0	0	0	0	0	0
0	0	0.00942	0	0	0	0	0	0	0
0	0	0	0	0	0	0	0	0	0
0	0	0	0	0	0	0	0	0	0
0	0	0	0	0	0	0	0	0	0
0	0	0	0	0	0	0	0	0	0
0	0	0	0	0	0	0	0	0.000747	0.000592
0	0	0	0	0	0	0	0	0	0
0	0	0	0	0	0	0	0	0	0
0	0	0	0	0	0	0	0	0	0
0	0	0	0	0	0	0	0.000189	0	0
0	0	0	0	0	0	0	0	0.011444	0
0	0	0	0	0	0	0	0	0	0
0	0	0	0	0	0	0	0	0	0
0	0	0	0	0	0	0	0.001063	0.005038	0.001956
0	0	0	0	0	0	0	0	0	0
	0	0		0	0	0	0	0.001068	0
		0		0	0		0	0	0
		0		0	0		0	0	0
0			0						
0	0		0			0			
0	0		0			0			
0	0		0			0			
0	0		0			0	0		
0	0		0			0	0		
0	0		0		0	0	0		
0	0	0	0	0	0	0	0	0	0

0 0 0 0 0 0
0 0 0 0
0 0
0
0
0
0
0
0 0 0 0 0 0
0 0 0 0 0
0 0 0 0 0
0 0 0 0

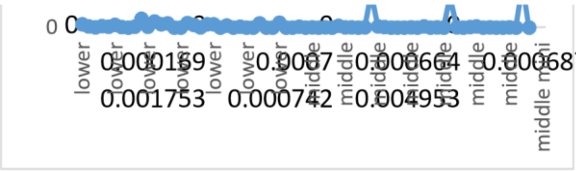

0

0 0.005715 0.002407
0.001601 0.007417 0 0 0 0 0 0 0 0 0 0
0 0 0 0.000638

0 0 0
0 0 0
0 0 0
0 0 0
0 0 0
0 0 0
0 0 0

		0	0		0		0		
		0	0		0	0	0		0
	0	0	0	0	0	0	0.00013		0.000164
	0	0	0	0	0	0	0	0.000303	0
	0	0	0	0	0	0	0	0	0
	0	0	0	0	0	0	0	0	0
	0	0.000223	0	0	0	0	0	0	0.002332
	0	0	0	0	0	0	0	0	0
0	0	0	0	0	0	0	0	0	0
0	0	0	0	0	0	0	0	0	0
0	0	0	0	0	0	0	0	0	0
0	0	0	0	0	0	0	0	0	0
0	0	0	0	0	0	0	0	0	0
0	0	0	0	0	0	0	0	0	0
0	0	0	0	0	0	0	0	0	0
0	0	0	0	0	0	0	0	0	0
0	0	0	0	0	0	0	0	0	0
0	0	0	0	0	0	0	0	0	0
0	0	0.00008	0	0	0	0	0	0.001522	0
0	0	0	0	0	0	0	0	0	0
0.000347	0	0.000061	0	0.000214	0	0	0.123271	0.203058	0.202187
0	0	0	0	0	0	0	0	0	0
0	0	0	0	0	0	0	0	0	0
0	0	0	0	0	0	0	0	0	0
0	0	0	0	0	0	0	0.000446	0.002325	0
0	0	0	0	0	0	0	0	0	0
0	0	0	0	0	0	0	0	0	0
0	0	0	0	0	0	0	0	0.000177	0
0.261146	0.23167	0.179822	0.16554	0.161642	0.130197	0.125714	0.3823	0.266054	0.176143
0	0	0	0	0	0	0	0.001492	0	0
0	0	0	0	0	0	0	0	0	0
0	0	0	0	0	0	0	0	0	0
0	0	0	0	0	0	0	0	0	0
0	0	0	0	0	0	0	0	0	0
0	0	0	0	0	0	0	0	0	0
0	0	0	0	0	0	0	0	0	0
0	0	0	0	0	0	0	0	0	0
0	0	0	0	0	0	0	0	0	0
0	0	0	0	0	0	0	0	0	0
0	0	0	0	0	0	0	0.026502	0	0
		0	0	0	0		0	0	0.003629
		0	0	0	0		0	0	0
		0.000332	0	0	0		0	0	0

0	0			0		0		0	0
0	0			0				0	
0								0	
0									
0									
0									
0									
0									
		0	0	0	0		0	0	0
		0	0.011913	0.008534	0		0	0	0
		0	0	0	0		0	0	0
		0	0	0	0		0	0	0
0	0	0	0	0	0	0	0	0	0.000292
0	0	0	0	0	0	0	0	0	0
0	0					0			
0	0					0			
0	0					0			
0	0					0			
0	0					0			
0	0					0			
0	0					0			

AHH21798
lower mini
0
0
0
0
0
0.00042
6 0
0
0
0
0
0
0
0.00096
2
0.01029
2 0
0.00014
0.001711
0
0
0
0
0.00032
2
0.00808
5 0
0
0.00171
7 0
0
0
0
0
0
0.00084
3 0
0.00046
3 0
0
0
0
0.00358
7 0
0.00036
3
0.002503

0

0

0.000387
0.00094
4 0
0.00200
7 0
0.00040
4 0
0.0124 0
0
0
0
0.00127
9 0
0
0
0
0.00050
1 0
0.09497
7
0.00281
5 0
0
0
0
0
0
0
0
0
0
0
0
0
0.00525
9 0
0.00097
1 0
0 0
.0025
48 0
0
0
0
0.029083
0.32722
0
0.00554
6 0
0

0

0
0
0
0
0
0
0
0
0
0.01087
2 0
0
0
0
0
0.00135
5 0
0.02469
7 0
0
0
0
0
0
0
0
0
0
0.00198
6 0
0
0
0
0
0
0
0
0
0
0.00051
1 0
0
0
0
0
0.01701
3 0
0
0

0

0
0.00029
9 0
0
0
0.00050
3 0
0
0
0
0
0
0
0
0.00173
5 0
0
0
0
0
0
0
0
0.00183
3 0
0
0
0.00194
3 0
0
0
0
0
0
0
0
0
0.013187
0.006372
0
0.00104
8 0
0
0
0
0
0
0
0

0

0
0
0
0
0
0
0
0.00182
2 0
0
0
0
0
0
0
0
0
0
0
0
0
0
0
0
0.01081
7 0
0
0.00101
8 0
0
0
0
0
0
0
0
0
0
0
0
0
0.0008
5 0
0
0
0
0
0
0
0

0

0
0.00110
5 0
0
0.21066
8 0
0
0
0
0
0
0
0.17158
3 0
0
0
0
0.00046
7 0
0
0
0
0
0.00055
9 0
0
0
0
0
0
0

0

0
0
