# Supplementary material for: Microbiome in the hair follicle of androgenetic alopecia patients
Source: PLoS One. 2019 May 3;14(5):e0216330. doi: 10.1371/journal.pone.0216330 (PMC6499469; doi:10.1371/journal.pone.0216330)
Supplement: S2 Table — Predominant Burkholderia species in middle and lower piece of vertex and occipital hair from patient and healthy controls. (PDF) [file pone.0216330.s004.pdf]

**Middle**	control occipital	control vertex	patient occipital	patient vertex
B. Cepecia	1	6	1	3
B. Contaminans	4	4	6	7
B. Cenocepacia	1	0	2	1
B. Kururiensis	0	0	1	1
B. wp sp26	0	0	1	1

**Lower**	control occipital	control vertex	patient occipital	patient vertex
B. Cepecia	3	2	8	2
B. Contaminans	7	4	9	13
Unclassified	0	0	0	1

S2 Table. Predominant *Burkholderia* species in hair samples. Predominant *Burkholderia* species in middle and lower piece of vertex and occipital hair from patient and healthy controls
